# Internally inlaid SaCas9 base editors enable window specific base editing

**DOI:** 10.7150/thno.70869

**Published:** 2022-06-06

**Authors:** Lurong Jiang, Jie Long, Yang Yang, Lifang Zhou, Jing Su, Fengming Qin, Wenling Tang, Rui Tao, Qiang Chen, Shaohua Yao

**Affiliations:** Laboratory of Biotherapy, National Key Laboratory of Biotherapy, Cancer Center, West China Hospital, Sichuan university, Renmin Nanlu 17, Chengdu 610041, Sichuan, China.

**Keywords:** CRISPR/Cas9, base editing, editing window, off-target, thalassemia

## Abstract

**Rationale:** Base editors composed of catalytic defective Cas9 and cytosine or adenosine deaminase are powerful tools to convert bases in a genome. However, the fixed and narrow editing window of current base editors has impeded their utility. To increase the scope and diversify the editing patterns is quite necessary.

**Methods and Results:** We designed a subset of base editors derived from SaCas9 in which deaminase was inlaid into various locations of the SaCas9 protein. The resulting base editors were characterized with multiple genomic sites and were found to have distinct editing features to the N-terminal SaCas9 CBE (Sa-CBE-N). Among them, Sa-CBE-693, in which a cytosine deaminase was inserted between amino acids 693 and 694, showed an increased editing efficiency and a significantly expanded editing window ranging from bases 2-18. This feature enhanced the editing efficiency of *BCL11A* enhancer that contains multiple consensus bases in a 15-bp fragment. Another variant, Sa-CBE-125, displayed backward-shifted editing window, which we showed was particularly powerful in editing cytosines that were accompanied with unintended bystander cytosines at their 5' side. Additionally, these editors showed reduced Cas9 independent DNA off-target editing compared with Sa-CBE-N.

**Conclusion:** Our inlaid base editors improved the targeting scope and diversified the editing pattern.

## Introduction

Clustered regularly interspaced short palindromic repeat/CRISPR-associated protein 9(CRISPR/Cas9)-derived base editors enable precise and efficient conversion of one base pair to another (C/G→T/A, or A/T→G/C) in targeted genomic DNA, and seldom cause double-strand breaks (DSBs) 1-4. Base editors are composed of a catalytically impaired Cas9 protein and a cytidine (CBE, 1, 5) or adenosine deaminase(ABE, 2) that is active on single-strand DNA substrates 1, 2, 5. In addition, Cas9 protein can be coupled with both deaminases to form dual base editors (CABE) that simultaneously convert both C and A 6-9. CBEs can also be modified to facilitate C to G conversions by coupling with UNG or other base excision repair factors 10, 11. Cas9 binds to its target DNA through single guide RNA (sgRNA) to form a protein/RNA/DNA ternary “R-loop” complex 12, 13. The non-target DNA strand (NTS) of the sgRNA that is complementary to the target strand is partially exposed outside the complex, thereby providing a feasible substrate for the deaminase to act upon.

In the original base editors, deaminase was coupled to the Cas9 complex by direct fusion to the N-terminus of Cas9 nickase that lacked catalytic activity to break the NTS 1, 5, 14. This design enabled the deaminase to convert bases within a small window of the NTS, called the editing window. For example, one of the most popular Streptococcus pyogenes Cas9 (SpCas9) -derived cytosine base editors, the BE3 variant, usually catalyzed the conversion of bases at positions 4-8 (counting the 5′-NGG-3′ protospacer adjacent motif (PAM) as being at positions 21-23) 1, 3.

To increase the targeting scope of base editors, several new ways to couple deaminases with Cas9 nickase have been designed. We and others have positioned the deaminase into internal sites of the Cas9 protein by circularly permutating or directly inlaying 15, 16. We also have placed the deaminase at various locations in the Cas9-sgRNA complex by installing RNA affinity tags to different stems of the sgRNA, which resulted in the regional recruitment of the deaminase that was fused with the RNA-tag binding protein 17. These redesigned architectures gave the base editors specific editing windows. Although these designs have been characterized extensively in SpCas9-derived base editors, they have been less well investigated in the minimal Cas9 ortholog from Staphylococcus aureus (SaCas9), another popularly used Cas9 that has similar activity to SpCas9 in both indel formation and base editing 18, 19. Importantly, SaCas9 has a relative larger editing window than SpCas9 in the same architecture of base editors 20, such as BE3, in which the cytosine deaminase was fused to Cas9 nickase through an X-ten linker 1. The editing window of classical N-terminal linked SaCas9 CBE variants (Sa-CBE-N) usually ranged from bases 3-11 (counting 5′-NNGRRT-3′ as 22-27) 19, indicating a much greater exposure of the NTS in SaCas9/sgRNA/DNA complex than that in SpCas9 complex 21. This feature endows SaCas9-derived base editors much more room for improving the editing window compared with SpCas9-derived base editors.

Here, we took advantage of the crystal structure of SaCas9/sgRNA/DNA complex to design a series of base editors in which cytosine or adenosine deaminase was inlaid into various locations within SaCas9; therefore, the deaminase was in different locations relative to the NTS. We characterized the performances of the resulting base editors and found that they had distinct editing scopes and showed lower off-target editing than Sa-CBE-N. These inlaid Sa-CBEs or Sa-ABEs, together with previously reported base editors engineered from SpCas9, greatly improve the targeting scope and provide additional choices for optimizing the editing outcomes.

## Results

### Design and characterization of SaCas9 cytosine base editors with internally inlaid deaminase

**Figure 1:**
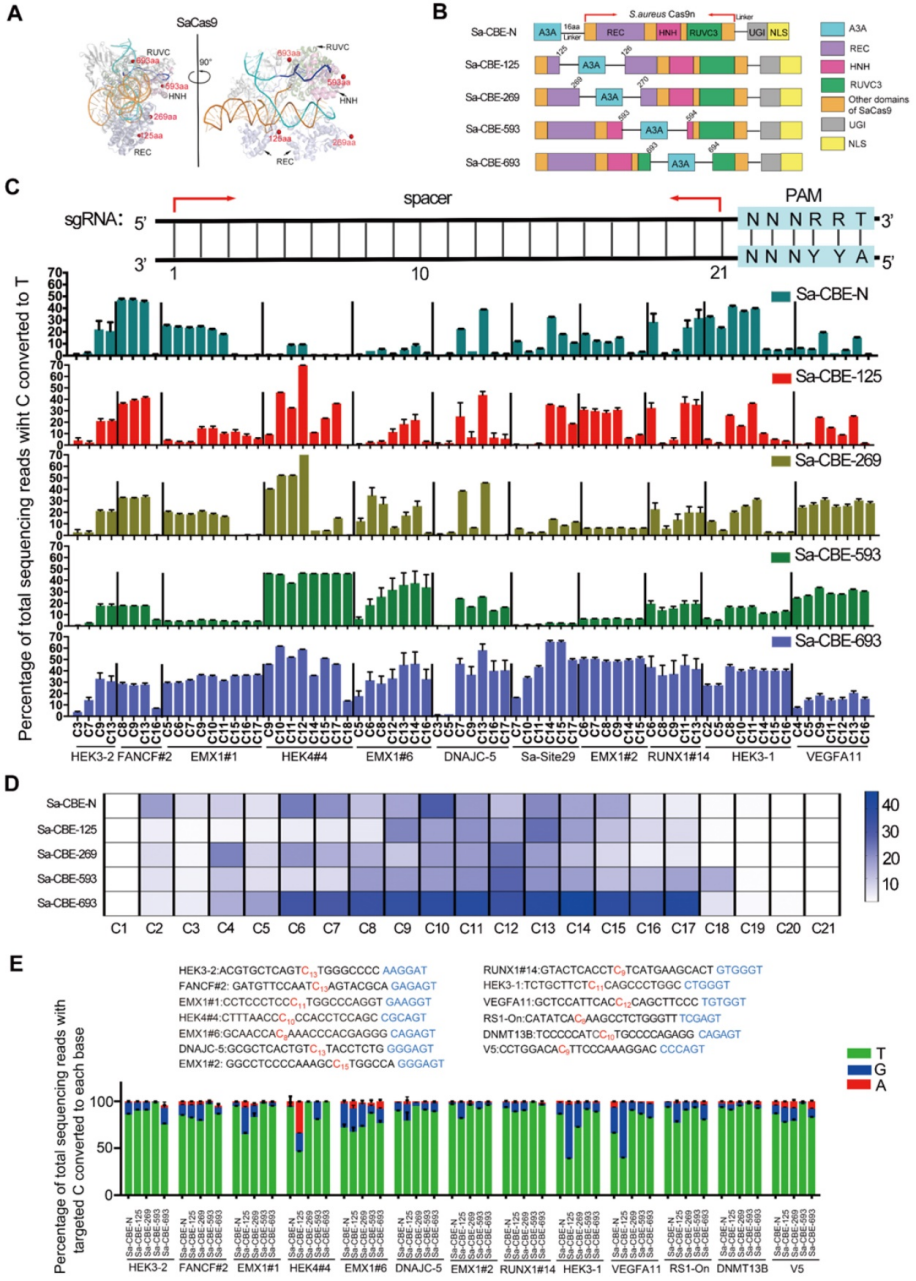
** Inlaying APOBEC3A into the SaCas9 domain diversified the editing windows. A.** Cartoon representations of the structure SaCas9/RNA/DNA complex (PDB 5axw). The amino acids E125, D269, S593 and R693 of SaCas9 are shown as red spheres. NTS corresponding to the sgRNA spacer was extracted from SpCas9 complex (PDB 5y36) that is structurally aligned with SaCas9 complex. **B.** Cartoon representations showing the architectures of Sa-CBE-N and inlaid Sa-CBEs (Sa-CBE-125, Sa-CBE-269, Sa-CBE-593 and Sa-CBE-693). A3A, human APOBEC3A; 16aa, the 16aa X-ten linker; UGI, uracil glycosylase inhibitor; NLS, nuclear localization signal. **C.** Comparison of C-to-T editing efficiency produced by Sa-CBE-N and inlaid Sa-CBEs at 11 endogenous human genomic loci. The PAM sequences (NNNRRT, 22-27) were highlighted in cyan (upper panel). Base editing efficiencies were analyzed by HTS. Values and error bars reflect the mean ± SD of 3 independent experiments. **D.** Heat map showing the average editing efficiency of SaCas9 derived CBEs at each position across 23 sites. **E.** The product distribution among edited DNA sequencing reads (reads in which the target C is converted) is shown for Sa-CBE-N and inlaid Sa-CBEs. The position that has C to R conversion is indicated in red. Values and error bars reflect the mean ± SD of 3 independent experiments. Editing efficiencies were measured by High throughput sequencing (HTS).

Because the current 3-dimensional structures of the SaCas9 complex lacked the information of the full-length NTS, we compared the structures of SaCas9 (PDB:5AXW) 21 and SpCas9 harboring the NTS (PDB:5Y36) 22 to simulate the position of the NTS in the SaCas9 complex. As shown in Figure S1, the general structures and protein folding of the Cas9 proteins in these complexes were conserved, and, importantly, the relative positions and orientations of the protein, sgRNA, and each DNA strand in the two complexes were almost the same. Therefore, we were able to use the structure of the SpCas9 complex, to simulate the structure of the SaCas9 complex with NTS included (Figure 1A).

Under the guidance of the structural information, we designed four inlaid Sa-CBEs in which the human apolipoprotein‐B mRNA‐editing catalytic polypeptide‐like 3 protein APOBEC3A was inlaid between amino acids (aa) 125 and 126, 269 and 270, 593 and 594, and 693 and 694 of the SaCas9 protein, respectively (Figure 1A-1B). These positions were chosen because 1. they are in the unstructured loops located on the surface of SaCas9 complex, so that the insertions are unlikely to interrupt the folding of SaCas9 protein and 2. they located in different directions relative to the NTS, so as to endow inlaid base editors with different editing windows. We used SaCas9-KKH variant to construct the inlaid base editors because this variant has broader PAM compatibility 23. The resulting inlaid base editors were named as Sa-CBE-125, Sa-CBE-269, Sa-CBE-593, and Sa-CBE-693 according to the inlaid positions. To evaluate their editing activities, we co-transfected each base editor together with a set of sgRNAs targeting 23 endogenous sites into human HEK293 cells (Table S1). All these target sites harbored multiple cytosines in their spacers. As depicted in Figure 1C and Figure S2A, the four base editors produced considerable C to T conversions across the 23 target sites. Among them, Sa-CBE-693 was most efficient, and its editing window ranging from bases C2-C18 and peaking at C6-C17, was wider than that of Sa-CBE-N (C2-C15). Sa-CBE-593 also had a wider editing window (ranging from C2 to C18, peaking at C12), but its efficiency was much lower than that of Sa-CBE-693.The low efficiency of Sa-CBE-593 may stem from steric hindrance of aa593 that is located opposite the NTS or from other factors such as disruption of native protein folding. Sa-CBE-269 had a similar window to Sa-CBE-N. And Sa-CBE-125 had a backward-shifted editing window (ranging from C9 to C17, peaking at C13,) that was slightly closer to the PAM than Sa-CBE-N editing window (Figure 1D). Therefore, these inlaid Sa-CBEs had diverse editing features, providing an opportunity for optimizing editing strategy for target sites in which the targeted cytosines located close to the PAM and accompanied with by-standers at their 5' side. These observations also confirmed the notion that positioning deaminase to different sites resulted in different editing windows.

It is well known that cytosine base editing resulted in impure C to R conversions besides C to T (R = A or G). To gain an overall insight into the product purity of those inlaid Sa-CBEs, we analyzed their performance on the target sites that have considerable C to R conversions (Figure 1E). The analysis revealed that inlaid Sa-CBEs exhibited different rates of C to R conversions from Sa-CBE-N. Among the inlaid Sa-CBEs, Sa-CBE-125 produced the most robust C to R conversions, the level of which varied dramatically from site to site (~8.82 % to 61.1%, Figure 1E). The high variation of efficiency could be possibly resulted from different local sequence contexts of these target Cs, as suggested by a previous study that investigate the determinants of base editing outcomes from large-scale library analysis 24. Overall, the average level of C to R conversions by Sa-CBE-125 was about 2.17 times more than that by Sa-CBE-N.

Because some inlaid editors, such as Sa-CBE-693, achieved a high level of editing at multiple cytosines, we next determined if they also edited the cytosines outside of the sgRNA spacer. The analysis did identify minimal levels of cytosine conversions in each flanking region of the sgRNA spacer (for most cases, less than 1% Figure S2B). However, compared to Sa-CBE-N, Sa-CBE-693 and other internal editors did not obviously increase the level of outside editing on all of target sites except for site HEK4#4. On this site, Sa-CBE-693 produced much higher level of C to T conversion at the position 8 bp downstream the spacer as compared to Sa-CBE-N (Sa-CBE-693: Sa-CBE-N = 9.16%: 0.41%). In addition, we also examined the levels of indels in the editing products and found that they seemed to be positively correlated with the levels of on-target editing, consistent with a model in which CBE mediated indels stem from the repair of apurinic site (AP site) generated by the removal of urine. And Sa-CBE-693 was found to induce highest level of indels in most target sites (8 out of 11, ranging from 1.53% to 10.3% Figure S2C). Because A3A prefers TC and CC motifs, we next analyzed if domain inlaying altered its motif preference. The analysis revealed that Sa-CBE-125 and Sa-CBE-269 has similar motif preference to N-terminally linked Sa-CBE that strongly prefer TC and CC motifs. However, Sa-CBE-593 and Sa-CBE-693 improved the editing efficiencies of GC and AC motifs while preserving the preference to TC and CC motifs (Figure S3).

### Design and characterization of SaCas9 adenine base editors with internally inlaid deaminase

**Figure 2:**
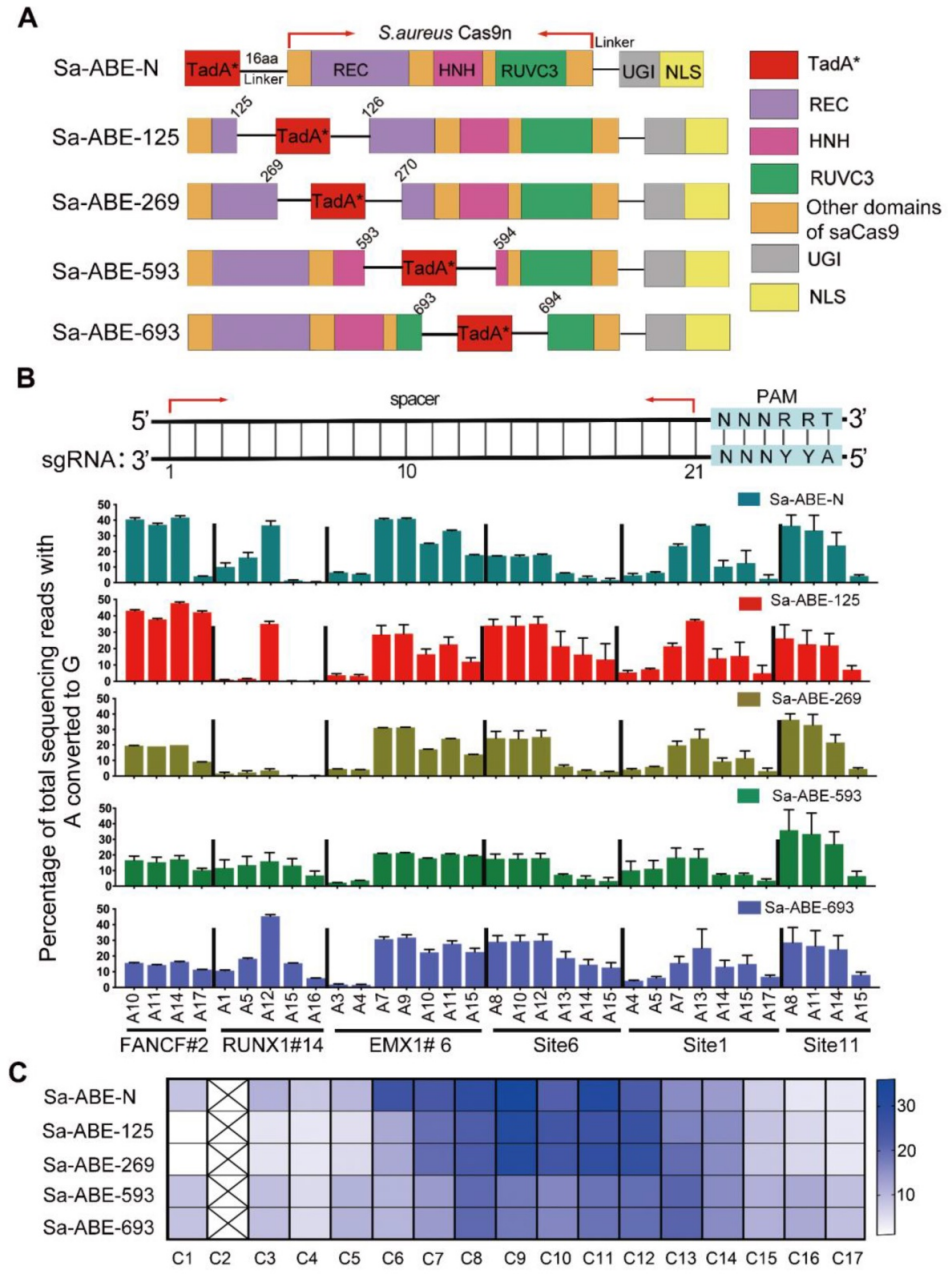
** Efficiency of adenine editing with inlaid SaCas9 adenine base editors. A.** Cartoon representations showing the architectures of Sa-ABE-N and inlaid Sa-ABEs. TadA*, evolved TadA-8e; 16aa, the 16aa X-ten linker; NLS, nuclear localization signal. **B.** Comparison of the A→G editing frequencies of Sa-ABE-N and by inlaid Sa-ABEs at 6 endogenous human genomic loci. The PAM sequences were highlighted in blue. The target As of protospacer at each target site were shown in black. Data were generated from three independent experiments and represented as mean ± SD. Editing efficiencies were measured by HTS.** C.** Heat map showing the average editing efficiency of SaCas9 derived ABEs at each position across 21 sites.

Inspired by the success of the inlaid Sa-CBEs, we constructed four Sa-ABEs by inlaying a recently engineered adenine deaminase, TadA-8e, into the corresponding positions of each Sa-CBE, and named them Sa-ABE-125, Sa-ABE-269, Sa-ABE-593, and Sa-ABE-693 according to the inlaid positions (Figure 2A). We tested the performance of these Sa-ABEs on 21 endogenous target sites, each of which harbored multiple adenines within the putative editing window (Table S2). As shown in Figure 2B and Figure S4A, the four base editors produced robust A→G editing across all the target sites, and the efficiency was comparable to that of N-terminal linked SaCas9 ABE variants (Sa-ABE-N). However, surprisingly, the editing windows of these inlaid Sa-ABEs were similar to that of Sa-ABE-N, unlike the editing windows of the inlaid Sa-CBEs, which were quite distinct (Figure 2C). In addition, we analyzed the product purity of those ABEs and found very rare conversions of A to C or T and only minimal levels of indels (0.12% - 3.97%, Figure S4B, C) in the edited products, which was consistent with the reported features of ABE 2, 23.

### Sequence independent DNA off-target editing of Sa-CBEs/ABEs

**Figure 3:**
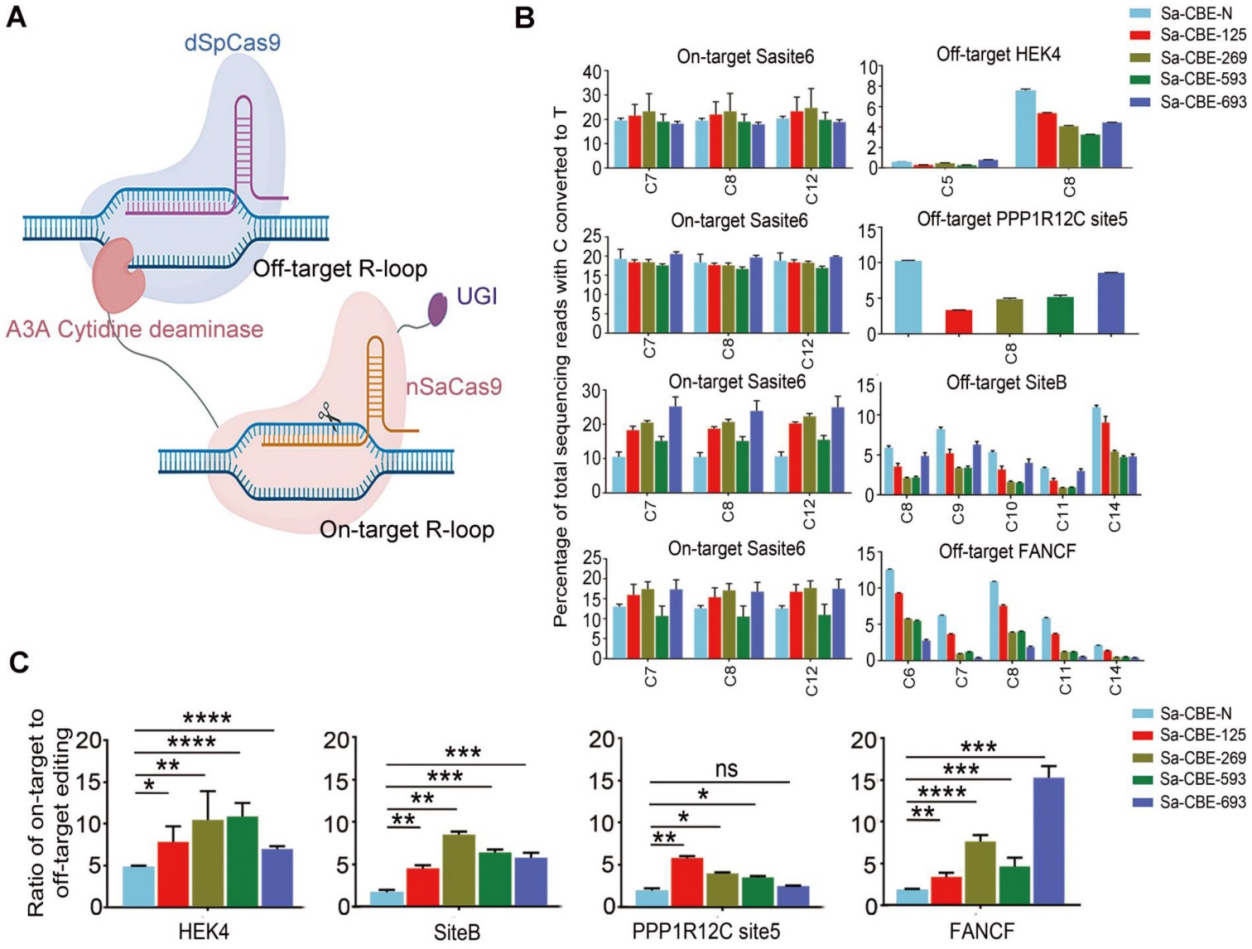
** Cas9 independent DNA off-target editing of inlaid Sa-CBEs. A.** Schematic diagram showing the mechanism of artificial R-loop assays. Off-target R-Loop consisted of dead SpCas9 and corresponding sgRNAs. On-target R-Loop consisted of SaCas9 derived CBEs and corresponding sgRNAs. Adapted from “CRISPR/Cas9 System”, by Biorender.com (2022). Retrieved from https://app.biorender.com/biorender-templates. **B.** Comparison of the DNA off-target editing induced by Sa-CBE-N and inlaid Sa-CBEs at four off-targets. Plasmids encoding paired R-loops were co-transfected into HEK293T cells and on- and off-target editing efficiencies were determined by HTS analysis of the target region. All Data were generated from three independent experiments and represented as mean ± SD. **C.** Quantification of the ratios of on-target efficiencies (averaged from each editable Cs) relative to off-target efficiencies (averaged from each editable Cs). Values and error bars reflect the mean ± SD of 3 independent experiments. Asterisks indicate statistically significant differences in editing efficiencies observed between Sa-CBE-N and inlaid Sa-CBEs at each site. (P ≥ 0.05, *P < 0.05, **P < 0.01, ***P < 0.001, ****P < 0.0001 by two-tailed Student's t test). Editing efficiencies were measured by HTS.

Off-target editing, especially sequence independent DNA off-target editing of base editors, produce genome-wide single-nucleotide variations 25, 26. Artificial R-loop assays have frequently been used to evaluate such off-target editing, and the results were very consistent with those from genome-wide deep sequencing 27, 28. To evaluate the sequence independent off-target editing of Sa-CBEs, we performed an artificial R-loop assay by using catalytically inactive SpCas9 to target four well-characterized off-target R-loop 27, HEK4, SiteB, PPP1R12C site5 and FANCF (Figure 3A and Table S3). We found that that all inlaid Sa-CBEs displayed reduced sequence independent off-target editing as compared to Sa-CBE-N across all artificial R-loops, while their on-target activities were comparable to that of Sa-CBE-N (Figure 3B). An analysis of the ratios of on- to off-target editing also supported the notion that internally inlaying reduced off-target effect, although the level of which varied with the loop and the type of editor (Figure 3C). We also detected the off-target effects of inlaid ABEs and N-terminal ABE by using R-Loop assay. As shown in Figure S5, we observed only mild levels of off-target effect across all ABEs tested (less than 0.5%).

### Simultaneous conversion of cytosine and adenosine with Sa-CABE

**Figure 4:**
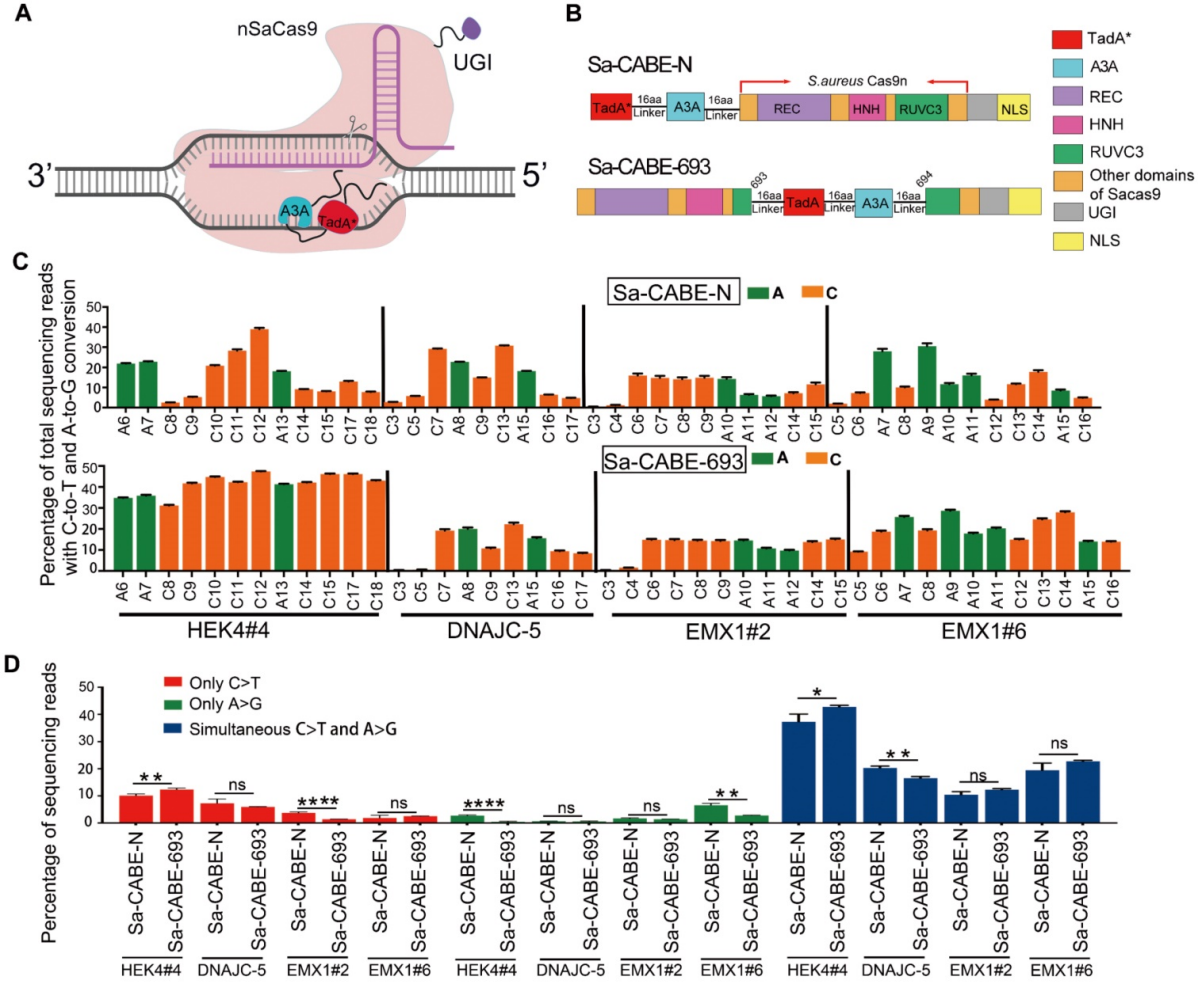
** Sa-CABE-693 induces simultaneous A→G and C→T base edits in human HEK293T cells. A.** Schematic diagram of Sa-CABE-693 architecture. Adapted from “CRISPR/Cas9 System”, by Biorender.com (2022). Retrieved from https://app.biorender.com/biorender-templates. **B.** Cartoon representations showing the architectures of Sa-CABE-N and Sa-CABE-693. A3A, human APOBEC3A; TadA*, evolved TadA-8e; 16aa, the 16aa X-ten linker; NLS, nuclear localization signal. The content represented by different color rectangles is displayed on the right side. **C.** Comparison of the C-to-T and A-to-G base-editing frequencies of Sa-CABE-693 and Sa-CABE-N at 4 endogenous human genomic loci. **D.** The product distribution among edited DNA sequencing reads in 4 endogenous human genomic loci. Values and error bars reflect the mean ± SD of 3 independent experiments. Asterisks indicate statistically significant differences in editing efficiencies observed between Sa-CABE-N and Sa-CABE-693 at each site. (P ≥ 0.05, *P < 0.05, **P < 0.01, ***P < 0.001, ****P < 0.0001 by two-tailed Student's t test). Editing efficiencies were measured by HTS.

Previous studies showed that simultaneously coupling cytosine and adenine deaminases to Cas9 protein (CABE) allowed the simultaneous conversion of both bases in a given target site 6-9, making it a powerful tool for disrupting regulatory elements and scalable mutation screenings. Because Sa-CBE-693 had an enlarged editing window and produced the most efficient and reliable conversions across all target sites, we focused on position 693 to design a CABE for both cytosine and adenine base editing. As depicted in Figure 4A and 4B, a fusion peptide of TadA-8e and APOBEC3A was tandemly inserted SaCas9 in-between amino acids 693 and 694 through 16aa X-ten linkers to form Sa-CABE-693. The same fusion peptide was fused to the N-terminus of SaCas9 to form Sa-CABE-N.

To test if Sa-CABE-N and Sa-CABE-693 could simultaneously convert both cytosine and adenine, the bases were co-transfected with sgRNAs that targeted four different endogenous sites, each of which harbored multiple Cs and As in the putative editing window. As shown in Figure 4C both editors induced detectable simultaneous conversions. Compared to Sa-CABE-N, Sa-CABE-693 produced higher levels of simultaneous A and C conversions in 3 out 4 target sites (Figure 4D and Figure S6- Figure S13). Furthermore, the editing window of Sa-CABE-693 was wider than that of Sa-CABE-N. On 2 of the target sites (HEK4#4, and EMX1#6), Sa-CABE-693 achieved robust simultaneous cytosine and adenine conversions within a 12-nt window, ranging from 5th to 16th base (Figure 4C). An analysis of the product purity revealed that the ratios of C to R conversions produced by Sa-CABE-693 (range: 4.14%-21.94%, mean: 11.98%) were lower or comparable to those produced by Sa-CABE-N (range: 4.29%-31.57%, mean: 16.06%) (Figure S14A). The frequencies of unintended indels produced by Sa-CABE-693 (range: 1.06%-8.14%, mean: 3.08%) were comparable to those produced by Sa-CABE-N (range: 0.87%-6.45%, mean: 3.23%) (Figure S14B). We also compared the editing properties of Sa-CABE-693 to Sa-CBE-693 and Sa-ABE-693. The comparison revealed that Sa-CABE-693 was slightly higher than Sa-ABE-693 but lower than Sa-CBE-693 in terms of editing efficiency. The levels of C to R conversion produced by Sa-CABE-693 were much lower than those produced by Sa-CBE-693 and the frequencies of unintended indels produced by Sa-CABE-693 were also lower than those produced by Sa-CBE-693. In consistent with previous observations, Sa-CABE-693 showed very rare indels (Figure S15A) and untended base transversions (Figure S15B).

### Applications of Inlaid SaCas9 base editor in disease-relevant targets

**Figure 5:**
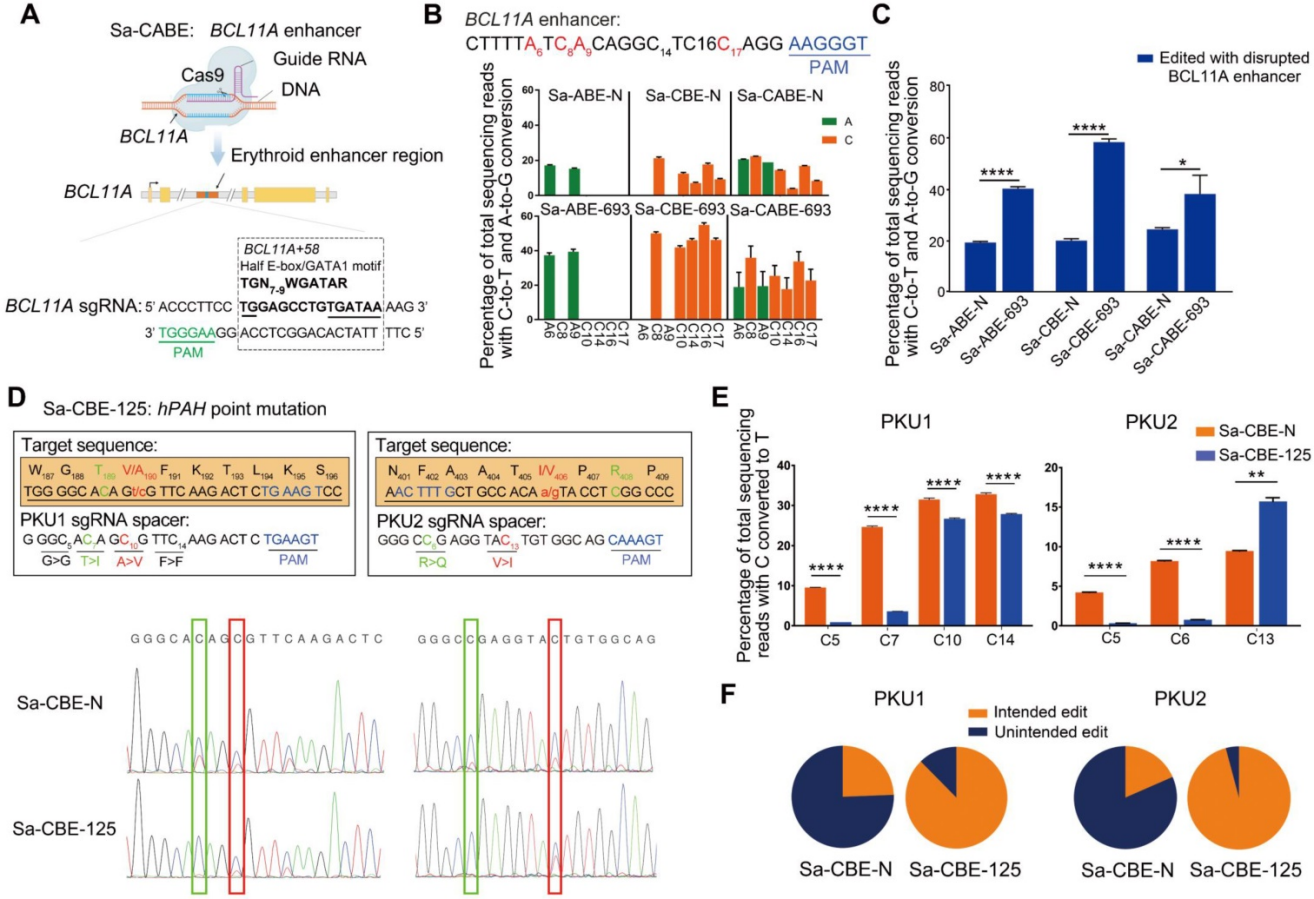
** Applications of Inlaid SaCas9 base editor in disease-relevant targets. A.** Cartoon representations showing the design of disrupting *BCL11A* enhancer with Sa-CABEs. Adapted from “CRISPR/Cas9 System”, by Biorender.com (2022). Retrieved from https://app.biorender.com/biorender-templates. The enhancer containing multiple consensus bases within a 15 bp fragment (TGN7-9WGATAR, where W = A or T and R = G or A) was highlighted in red. **B.** Base editing of the *BCL11A* enhancer by Sa-BE-693 editors or N-terminal editors in HEK293T cells. Editable As and Cs within *BCL11A* enhancer were shown in green and orange respectively, with a subscripted number denoting their relative position to PAM (counting NGG PAM as + 21 to + 23). All Data were from three independent experiments and represented as mean ± SD. **C.** The ratio of disrupted *BCL11A* enhancer in cells edited by N-terminal and 693 base editors. Values and error bars indicate mean ± SD of three independent experiments. (P ≥ 0.05, *P < 0.05, **P < 0.01, ***P < 0.001, ****P < 0.0001 by two-tailed Student's t test). **D.** Correction of two PKU related mutations with Sa-CBE-N and Sa-CBE-125. Upper panel shows the design of base editing strategy against A190V and I406V mutations, with sequences of each sgRNA underlined, and PAM sequences highlighted in blue. The bases responsible for the mutations are indicated in red with a subscripted number corresponding to its position within the protospacer. Intended and unintended conversions are shown in red and green respectively. Lower panel show the Sanger sequencing chromatograph of the alleles edited by indicated base editors. Editing efficiencies were measured by EditR 44. **E.** Quantifications of the editing efficiency of PKU mutations with HTS showed the efficiency of each editable Cs. Values and error bars indicate mean ± SD of three independent experiments. (P ≥ 0.05, *P < 0.05, **P < 0.01, ***P < 0.001, ****P < 0.0001 by two-tailed Student's t test). **F.** Quantification of the ratio of intended and unintended edits in PAH mutant sites. Values and error bars reflect the mean ± SD of three independent experiments. Editing efficiencies were measured by HTS.

The wide editing window of 693 derived base editors was extremely useful for targets that contained multiple bases to be edited. For example, a therapeutic enhancer in thalassemia, the half-E box/GATA1 binding site located in the *BCL11A* locus, is responsible for the erythrocyte expression of *BCL11A*, a key repressor of infant γ-globin expression 29. Accumulating evidence has shown that disruption of the *BCL11A* enhancer leads to activation of γ-globin expression in erythrocytes, thereby rescuing β-globin deficiency-related phenotypes 30, 31. The *BCL11A* enhancer contains multiple consensus bases in a 15-17-bp region (5′-TGN7-9WGATAR-3′, where W = A or T and R = G or A) 32. To test the ability of 693 derived base editors to disrupt the *BCL11A* enhancer, we designed a sgRNA as shown in Figure 5A. Transfection of this sgRNA together with the Sa-CBE-N, Sa-ABE-N, and Sa-CABE-N, resulted in obvious disruption of the consensus bases. The inlaid Sa-BE-693 editors, especially Sa-CBE-693 and Sa-CABE-693 significantly enhanced both editing efficiency and scope as compared to their N-terminal counterparts. (Figure 5B and Figure S16- Figure S21). The rates of disrupted *BCL11A* enhancer (at least one consensus base was converted) in Sa-BE-693 editors edited cells were about 2.14 times higher than that in N-terminal editors edited cells (Sa-ABE-693: Sa-ABE-N = 40.30%: 19.33%; Sa-CBE-693: Sa-CBE-N = 58.37%: 20.09%; Sa-CABE-693: Sa-CABE-N = 38.20%: 24.53%) (Figure 5C). Consistent with previous observation, compared to ABEs, the editors containing cytosine deaminase (CBEs and CABEs) produced higher levels of indels, with 693 variants being even higher (Sa-CBE-693: 5.02%, Sa-CABE-693: 4.93%) (Figure S22A). Considerable levels of C to R conversions were also noticed in CBEs or CABEs edited products, with Sa-CABE-N being the highest (12.79%) (Figure S22B).

The backward-shifted editing window of Sa-CBE-125 suggested that it could be used to improve the editing outcomes of target sites in which the targeted cytosines are located close to the PAM and accompanied with undesired by-standers at their 5' side. To prove this concept, we selected two human patient derived Phenylalanine hydroxylase (PAH) gene mutations, which lead to the loss-of-function of PAH enzyme that responsible for phenylalanine metabolism, resulting in hyperphenylalaninemia related syndrome (Figure 5D). As shown in Figure 5D, the two targeted Cs were mutated to Ts, resulting in amino acid substitutions in PAH protein (A190V for PKU target 1, and I406V for PKU target 2). Although Sa-CBE-N could convert both targeted Cs into Ts, it also converted unintended bystanders that located at 5' side of the targeted Cs, which changed the codons. As a comparison, Sa-CBE-125 seldom edited those bystanders (Figure 5D-5F). A detailed analysis of individual edited allele revealed that Sa-CBE-125 achieved 3.05 and 8.88 times higher pure intended conversion on PKU1 and PKU2 respectively as compared to Sa-CBE-N (22.89% VS 7.51% for PKU1 and 15.36% VS 1.73% for PKU2) (Figure 5F).

## Discussion

Classical base editors in which deaminases are fused to the N-terminus of Cas9 proteins, such as the BE3 and BE4 variants 33, catalyze the conversion of bases within a relatively fixed window upstream of the PAM. The limitation of base editing window is one of the main bottlenecks that hinder the application range of base editors, especially when the targeted base does not have a matched PAM. A possible way to improve the targeting scope is to position the deaminase at different locations in the Cas9 complex, so as to change the distance between the deaminase and the NTS and alter their relative positions. Such designs have been well characterized in SpCas9-derived base editors 15, 16, but are less well investigated in SaCas9, another frequently used Cas9 DNA editing system that is compatible for all-in-one AAV delivery because of its small size. In this study, we selected SaCas9 for deaminase inlaying. In the guidance of three-dimensional structure of an SaCas9-RNP complex, we designed a set of inlaid base editors and found that these novel base editors performed robust base editing and had editing windows that were different from those of N-terminal base editor. Therefore, these inlaid editors expanded the base editing tool box and provided a possible way of optimizing editing strategies for complicated targets. In addition, these results further demonstrated the notion that inlaying deaminases into Cas9 protein could fine-tune the base editing features. It is interesting to test if this notion could also be applied to other smaller Cas proteins, such as CjCas9 and Cas12f vectors 34, 35.

The editing features of these editors could be applied to design other SaCas9-derived editing tools by domain insertion. Previous studies on SpCas9 showed that Cas9 proteins tolerated a relatively wide range of insertions, without disrupting their binding and cleavage activities 36. Like SpCas9, SaCas9 has a bilobed architecture with an REC lobe (aa41-425) and a NUC lobe (aa1-40 and aa435-1053). The REC lobe contains an REC domain and a bridge helix that connects the REC domain with the N-terminal RUVC1 domain. The NUC lobe contains RUVC1-3, HNH, WED, and PI domains 21. All the domains in the NUC lobe, except WED, neighbor and face the NTS, which makes them good candidates for inlaying deaminases or other functional domains that are designed to target the NTS, such as cytosine methyltransferase and reverse transcriptase.

The internal inlaying of deaminases might limit their movement and restrict their interaction with RNAs or incidentally occurring single-strand DNAs, thereby attenuating Cas9-independent off-target editing. Indeed, we observed obviously reduced off-target editing of both molecules in two of our inlaid Sa-CBEs. Consistent with this observation, three recent reports also demonstrated that inlaid SaCas9 and SpCas9 base editors showed decreased Cas9-independent RNA or DNA off-target editing 37-39. In our R-loop assay, we noted that the editing efficiency for each cytosine in the artificial R-loop varied significantly, possibly because of the different accessibilities between the editable cytosines and the deaminase. It is interesting to examine if further limiting the movement of the deaminase by shortening the linkers between Cas9 and the internally inlaid deaminase could decrease such accessibility, thus reducing Cas9-independent off-target editing.

Interestingly, we found one inlaid CBE, Sa-CBE-125, inclined to yield a higher rate of C-to-G conversion. This type of conversion is demonstrated to be dependent on the activity of UNG that removes urine to generate an AP site. Although the subsequent process remains poorly understood 10, 40, 41, a recent report showed that the trans-lesion synthesis mechanisms take an important part in this process, since inhibition of trans-lesion factors reduced the rates of C to G conversion 42. The same report also found that Cas9 variants with reduced binding strength to the target DNA improved C -to-G conversion at certain target loci, which was possibly due to a reduced competition between Cas9 protein and repair machinery for access to the target locus edited by these variants 42. Therefore, a possible explanation of a higher rate of C-to-G in Sa-CBE-125 edited sites would be that the insertion of deaminase reduced the binding strength of SaCas9 to DNA, facilitating the entrance of repair machinery.

Among our inlaid Sa-CBEs, Sa-CBE-693 had a significantly enlarged editing window, ranging from bases 2-18, making it the widest editor reported so far. Such base editors can be extraordinary useful when wide editing ranges are required. In addition to destroying key bases within the targeted promoter or enhancer, these editors could be rewired to design large-scale saturation mutation screenings. For example, simultaneously inlaying both cytosine and adenine deaminases (Sa-CABE-693) enabled both C and A conversions, and when coupled with an sgRNA library covering a DNA fragment of interest, it enabled the efficient conversion of C→T and A→G within that fragment, thereby achieving targeted saturation mutation screenings. The wide editing window of Sa-CBE-693 also provides the possibility of editing targeted bases that are extremely close to the PAM. However, it is noteworthy that this property could also result in the lack of control of editing as multiple sites may be edited simultaneously, therefore a careful examination of the editing outcomes of each allele is required to interpret the screening properly. In addition, considering that Sa-CBE-693 produced more indels than Sa-CBE-N, when the screen requires high product purity, we recommend to add additional copies of uracil glycosylase inhibitor (UGI) to Sa-CBE-693 to extensively inhibit UNG to reduce the formation of indels as demonstrated by previous studies 33, 43.

In summary, we designed a series of inlaid SaCas9 base editors that has different editing features from N-terminal linked ones. Among these inlaid editors, Sa-CBE-125, displayed backward-shifted editing window, which was particularly powerful in editing cytosines that were accompanied with untended bystander cytosines at their 5' side. Sa-CBE-693 showed an increased editing efficiency and a significantly expanded editing window ranging from bases 2-18. Importantly, these inlaid CBEs showed reduced Cas9 independent DNA off-target editing compared with Sa-CBE-N. Therefore, these novel base editors expanded the editing scope and diversified the editing pattern, facilitating the optimization of the editing of complicated targets.

## Materials and Methods

### Design and construction of plasmid

Cytosine deaminase Apobec3A Y130F (A3A-Y130F) and TadA-8e were used in this study to construct corresponding SaCas9(KKH) derived CBEs, ABEs and CABEs. The structure of the SaCas9/sgRNA/DNA complex (PDB: 5axw) was analyzed with PyMOL program. A3A was inlaid in different positions relative to NTS, namely the 125^th^, 269^th^, 593^rd^ and 693^rd^ amino acids positions of SaCas9. The plasmids of inlaid Sa-CBEs and Sa-ABEs, Sa-CBE-N, Sa-ABE-N, Sa-CABE-N and Sa-CABE-693 were obtained by seamless cloning method (ClonExpress II one-step cloning kit. Vazyme Biotech Co. Ltd.), amino acids sequences are listed in Supplementary Sequences 1. The sgRNA expression vectors were generated by inserting each spacer sequence into Bbs1 digested empty plasmids. Oligo nucleotides used to generate sgRNA expression vectors are listed in documents: Table S1-S3. All plasmids were verified by Sanger sequencing.

### Cell culture

HEK293T cells were cultured in Dulbecco's modified Eagles's medium (Thermo Fisher Scientific), supplemented with 10% (v/v) fetal bovine serum (life technologies), 1% penicillin/streptomycin (Boster Biological Technology Co.Ltd) at 37 °C with 5% CO2.

### Plasmids Transfection

HEK293T cells were seeded on 24-well plates (BIOFIL). TranseasyTM (Forgene) was used to for plasmids transfection according to the manufacture's guidance. Briefly, HEK293T cells were seeded on 24-well plates at a concentration of ~1 x 10^5^ cells per well in 0.5 mL of complete growth medium. Transfections were performed when cell density reaching approximately 70%-80% confluent. A total amount of 1 μg DNA plasmids were transfected into each well. 72 h post the transfection, genomic DNA was extracted by adding 30 µL of freshly prepared lysis buffer. The mixture was incubated at 55 °C for 10 min and then inactivated at 95 °C for 10 min. The resulting genomic DNA was amplified by PCR and then analyzed by Sanger sequencing or High-throughput sequencing.

### Sanger sequencing and EditR analysis

Genomic DNAs extracted from transfected cells were used to amplify target regions with Phanta Max Super-Fidelity polymerase (Vazyme Biotech Co. Ltd). The amplicons were purified with gel-extract or PCR purification kit according to the manufacturer's instructions. Purified amplicons were subjected to Sanger sequencing and the resulting chromatographs were quantified by EditR software (baseditr. com), according to the author's description 44. Primers used to amplify flanking region of each on- or off-target site are listed in Table S4.

### High-throughput DNA sequencing and data analysis

Genomic DNA regions of interest were amplified with Phanta Max Super-Fidelity polymerase (Vazyme Biotech Co. Ltd) with primers harboring individual barcodes to distinguish different samples. Primers used to amplify flanking region of each on- or off-target site are listed in Table S5-S9. Amplicon Samples were sequenced commercially using Illumina HiSeq platform (Shanghai Personalbio Technology Co., Ltd.). Frequencies of base conversions and indels were quantitated with CRISPResso2 45.

### Statistical analysis

GraphPad Prism software (version 8.4.0) was used for all data analysis. All statistical comparison adjustment was performed using two tailed Student's t-test in SPSS software (version 21.0.0.0).

## Supplementary Material

Supplementary figures and tables.

## Abbreviations

CRISPR/Cas9: Clustered regularly interspaced short palindromic repeat/ CRISPR-associated protein 9
DSBs: double-stranded DNA breaks
CBE: Cytosine base editor
ABE: adenine base editor
CABE: cytosine and adenine base editor
sgRNA: single guide RNA
NTS: non-target DNA strand
PAM: protospacer adjacent motif
SpCas9: Streptococcus pyogenes Cas9
SaCas9: Staphylococcus aureus Cas9
AP site: apurinic site
PAH: Phenylalanine hydroxylase
A3A-Y130F: Apobec3A Y130F
UGI: uracil glycosylase inhibitor
NLS: nuclear localization signal
HTS: High throughput sequencing
