# Supplementary material for: Internally inlaid SaCas9 base editors enable window specific base editing
Source: Theranostics. 2022 Jun 6;12(10):4767–78. doi: 10.7150/thno.70869 (PMC9254239; doi:10.7150/thno.70869)
Supplement: Supplementary file 1 — Supplementary figures and tables. [file thnov12p4767s1.pdf]

Supplementary Materials for

Internally inlaid SaCas9 base editors enable window specific base editing

Supplementary Text

Figure S1 to S22

Tables S1 to S9

Supplementary sequences1

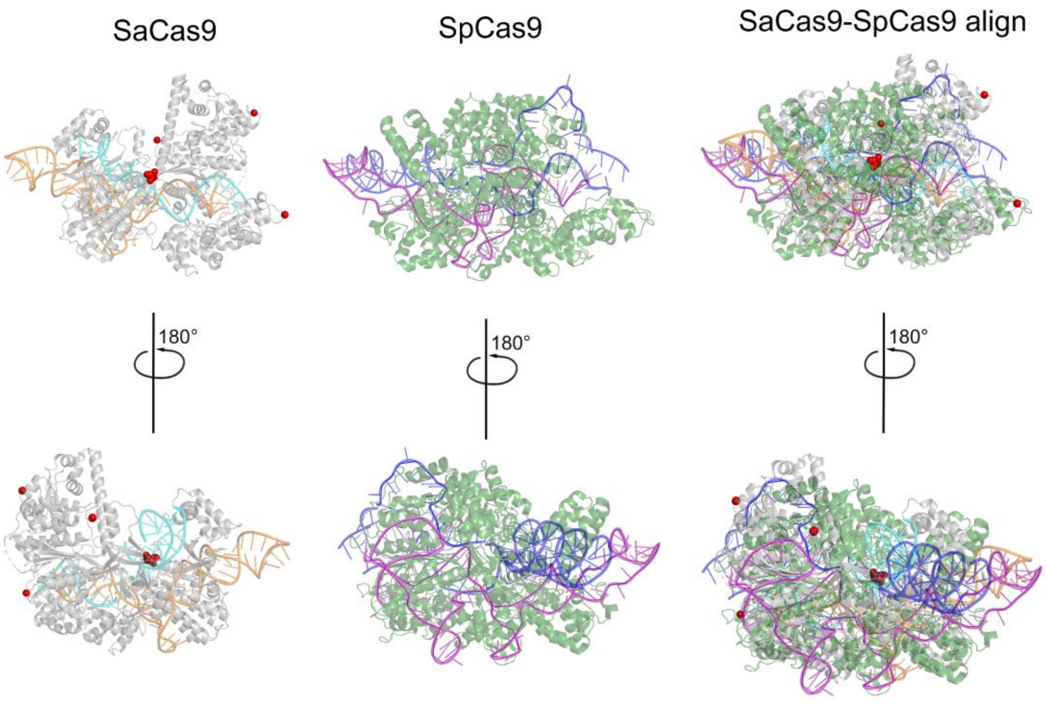

Figure S1. 3-dimensional structures of the SaCas9 complex and SpCas9 complex. Cartoon representations of the structure SaCas9/RNA/DNA complex (PDB 5axw) (left), SpCas9/RNA/DNA complex (PDB 5y36) (middle) and alignment of SaCas9/SpCas9 complex (right). The amino acids E125, D269, S593 and R693 of SaCas9 are shown as red spheres.

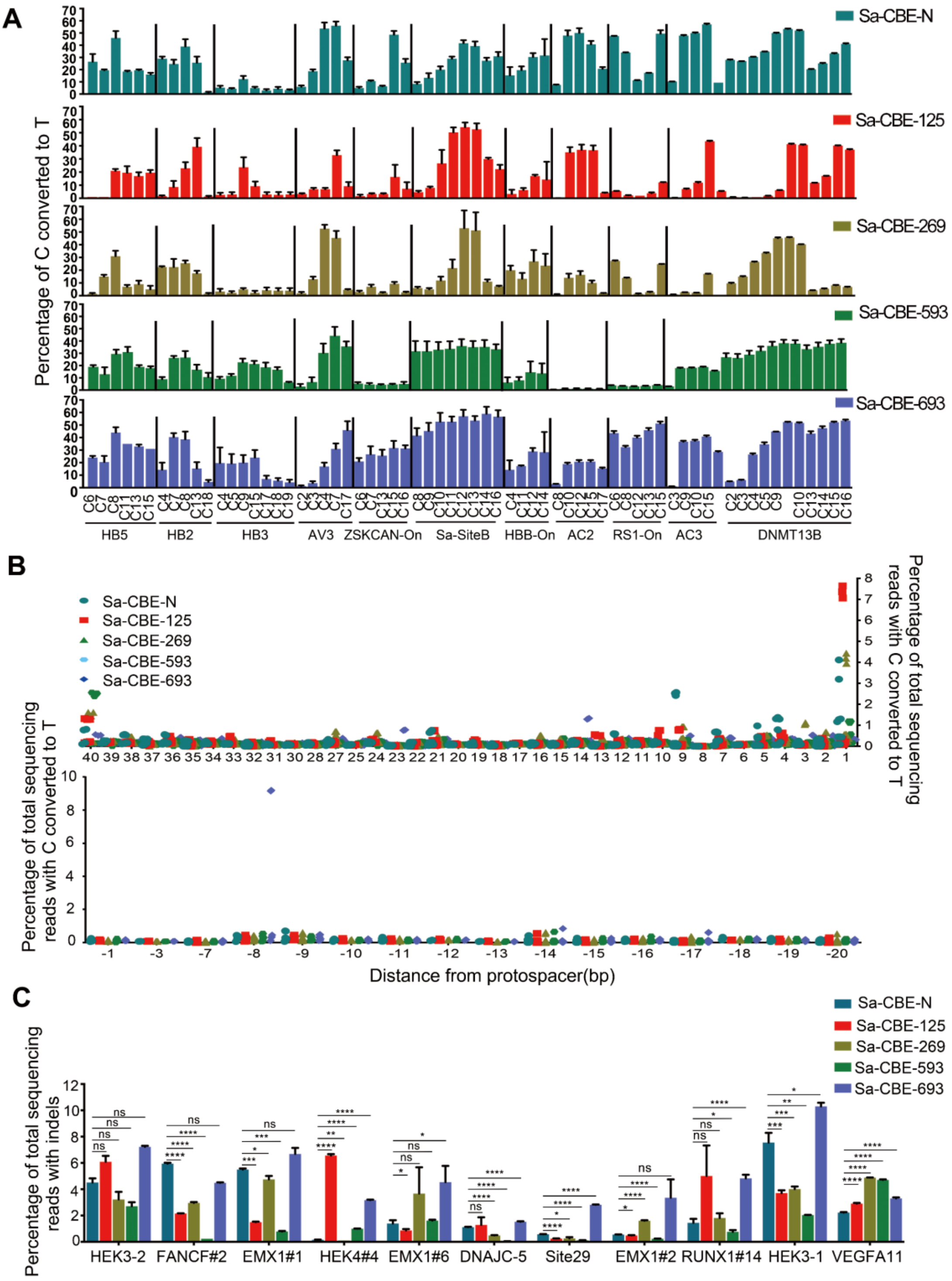

Figure S2. Additional data sets of Sa-CBE-N and inlaid-Sa-CBE editors editing outside of window and indel formation. A. Comparison of C-to-T editing efficiency produced by Sa-CBE-N and inlaid Sa-CBEs at additional 12 endogenous human genomic loci. Editing efficiencies were measured by Sanger sequencing and EditR

analysis. **B.** Cellular non-target C to T conversion percentages editing outside of window by Sa-CBE-N and inlaid-Sa-CBE editors are plotted individually against their positions relative to a protospacer for all 178 cytosines at 11 endogenous sites. The side of the protospacer distal to the PAM is designated with positive numbers, while the side that includes the PAM is designated with negative numbers. **C.** Indels frequencies following treatment of HEK293T cells with Sa-CBE-N and inlaid Sa-CBE editors at all 11 genomic loci. Indels frequencies were calculated as described in the Methods following treatment of HEK293T cells with Sa-CBE-N and inlaid Sa-CBE editors at all 11 genomic loci. Each experiment was repeated three times, data are represented as mean ± SD. Asterisks indicate statistically significant differences in editing efficiencies observed between Sa-CBE-N and inlaid Sa-CBEs at each site. ($P \geq 0.05$, *$P < 0.05$, **$P < 0.01$, ***$P<0.001$, ****$P<0.0001$ by two-tailed Student's t test). Editing efficiencies were measured by HTS.

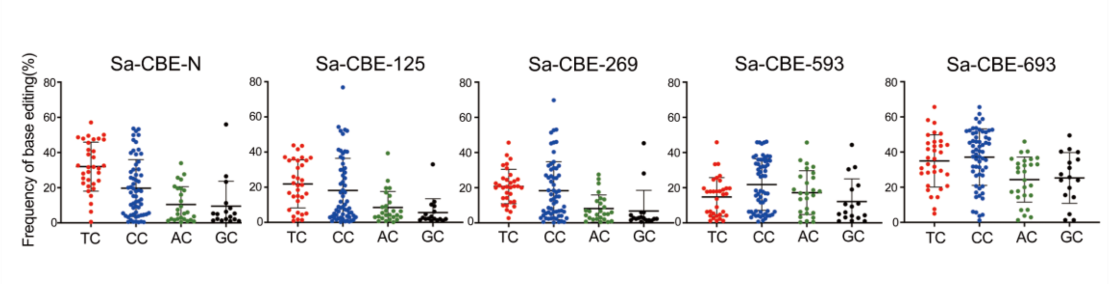

Figure S3. Comparison of base editing activity and sequence preference.

Frequencies of base editing of indicated motifs. Each dot represents the frequency of a specific. All Data were generated from three independent experiments and represented as mean ± SD.

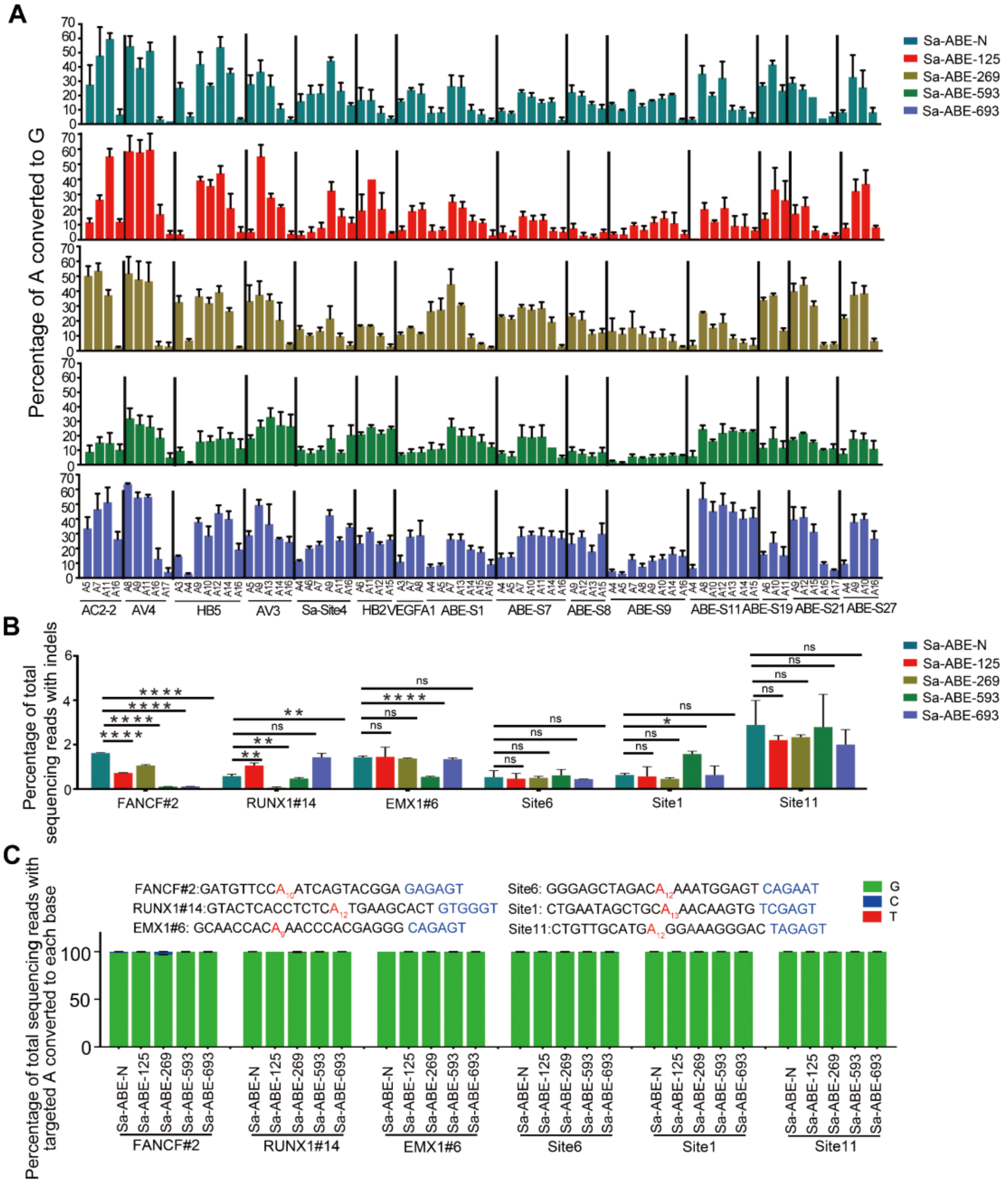

Figure S4. Indels frequencies and product purity following treatment of HEK293T cells with Sa-ABE-N and inlaid Sa-ABE editors at 6 genomic loci. A. Comparison of A-to-G editing efficiency produced by Sa-ABE-N and inlaid Sa-ABEs at additional 15 endogenous human genomic loci. Editing efficiencies were measured by Sanger sequencing and EditR analysis. **B.** Comparison of indels frequency produced by Sa-ABE-N and internally inlaid Sa-ABEs at 6 endogenous human genomic loci. Each experiment was repeated three times, data are represented as mean ± SD. Asterisks indicate statistically significant differences in editing efficiencies

observed between Sa-ABE-N and inlaid Sa-ABEs at each site. ($P \geq 0.05$, *$P < 0.05$, **$P < 0.01$, ***$P < 0.001$, ****$P < 0.0001$ by two-tailed Student’s t test). Editing efficiencies were measured by HTS. **C.** The product distribution among edited DNA sequencing reads (reads in which the target A is converted) is shown for Sa-ABE-N and inlaid Sa-ABEs. The position that has A to Y conversion is indicated in red. Each experiment was repeated three times, data are represented as mean ± SD.

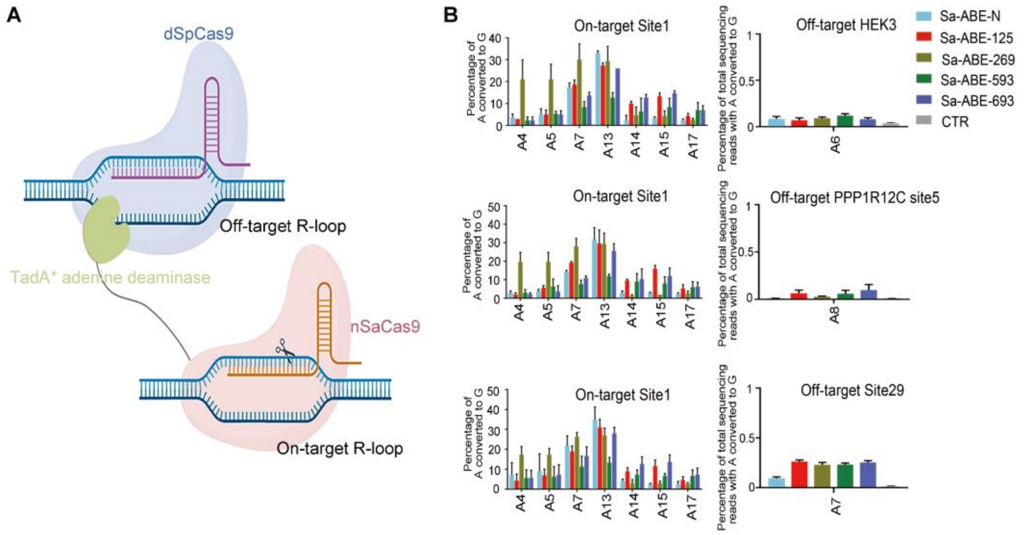

Figure S5. Cas9 independent DNA off-target editing of inlaid Sa-ABEs. A. Schematic diagram showing the mechanism of artificial R-loop assays. Off-target R-Loop consisted of dead SpCas9 and corresponding sgRNAs. On-target R-Loop consisted of Sa-Cas9 derived ABEs and corresponding sgRNAs. **B.** Comparison of the DNA off-target editing produced by Sa-ABE-N and inlaid Sa-ABEs at three endogenous loci. Plasmids encoding paired R-loops were co-transfected into HEK293T cells and on-target editing efficiencies were determined by Sanger sequencing and off-target analysis efficiencies were determined by HTS. All Data were generated from three independent experiments and represented as mean ± SD.

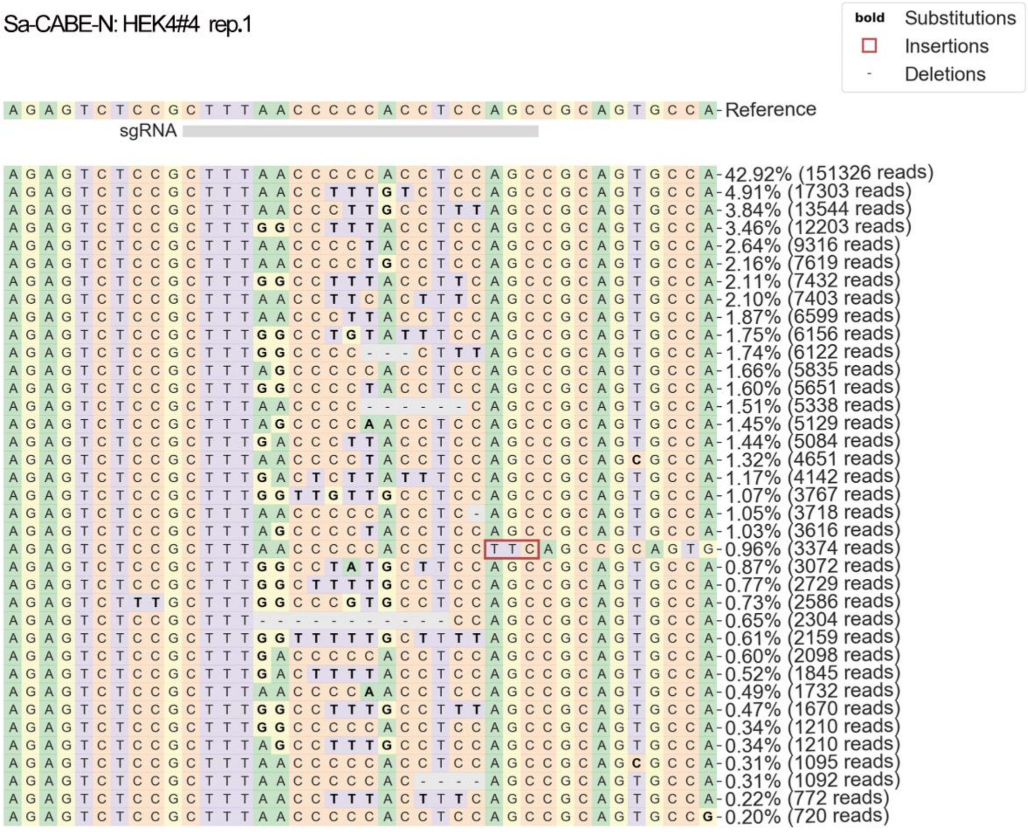

Figure S6. Allele frequencies following treatment with Sa-CABE-N at HEK4#4 genomic loci. Target site HEK4#4 is marked with a gray line. The percentile of unique sequencing read that represents a representative experiment from three independent experiments is listed on the right.

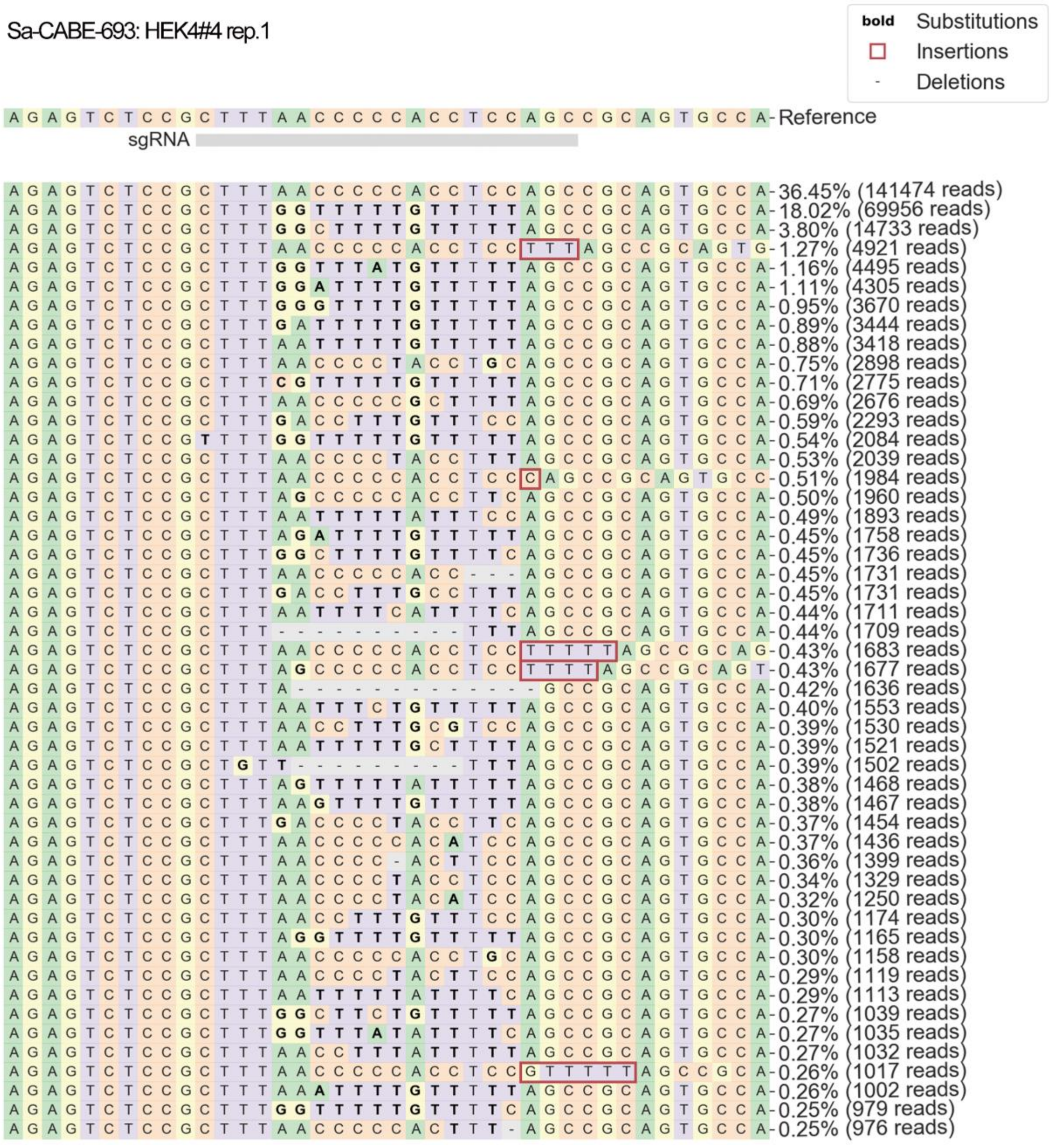

Figure S7.

Allele frequencies following treatment with Sa-CABE-693 at HEK4#4 genomic loci.

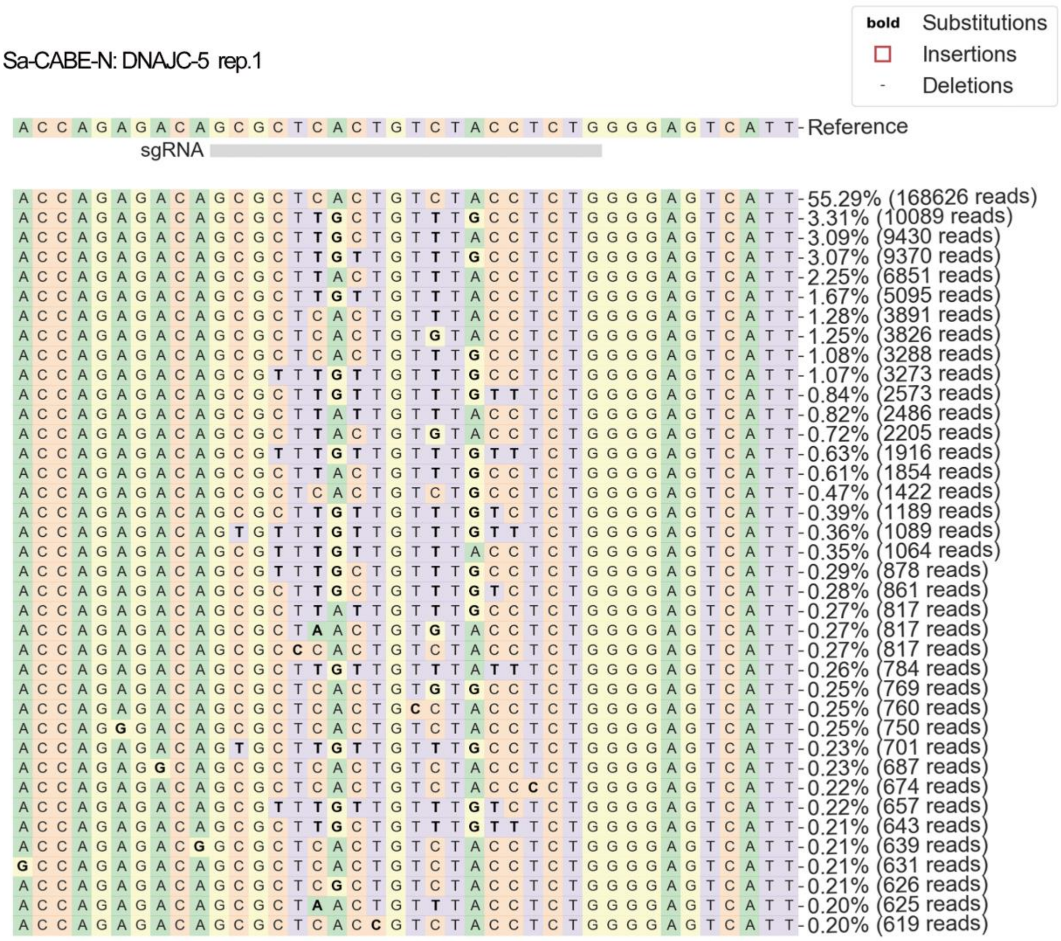

Figure S8.

Allele frequencies following treatment with Sa-CABE-N at DNAJC-5 genomic loci.

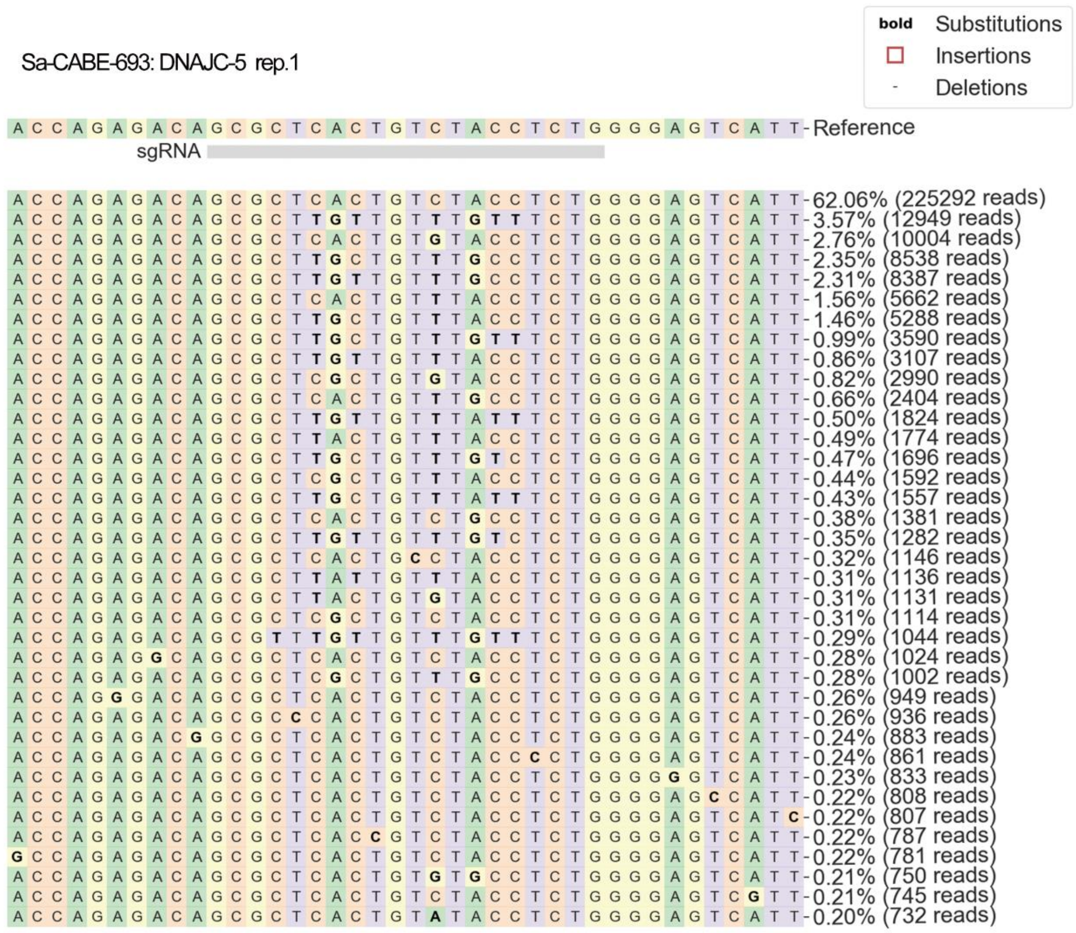

Figure S9

Allele frequencies following treatment with Sa-CABE-693 at DNAJC-5 genomic loci.

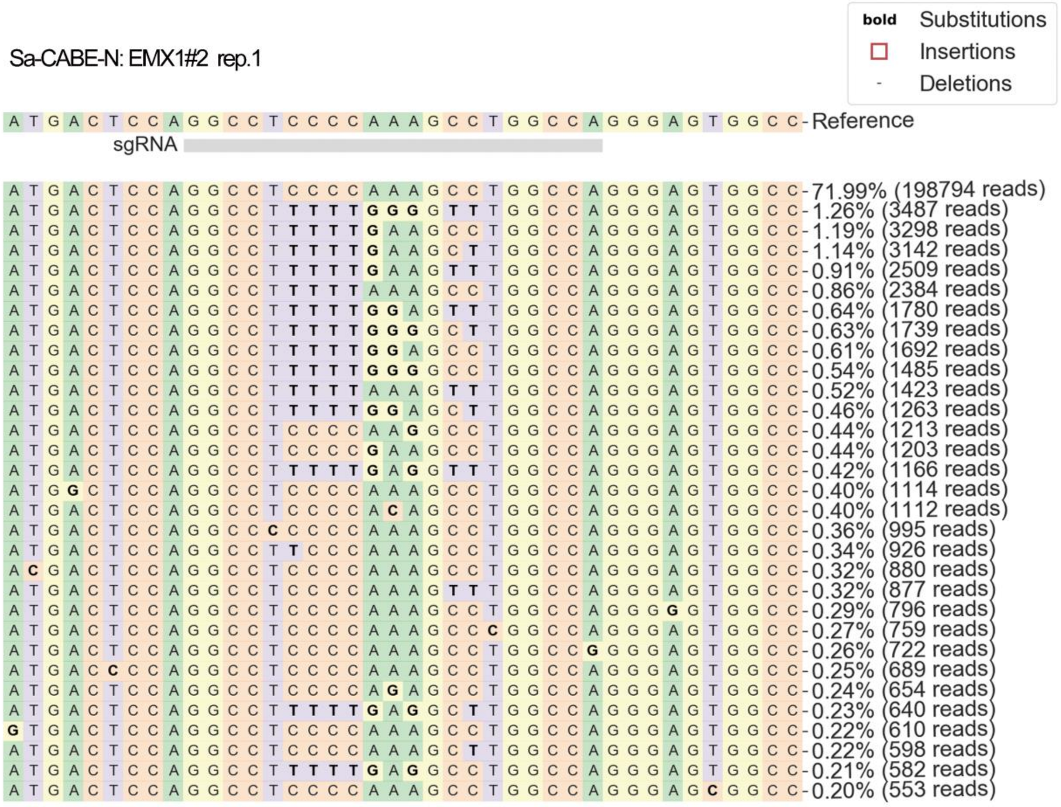

Figure S10.

Allele frequencies following treatment with Sa-CABE-N at EMX1#2 genomic loci.

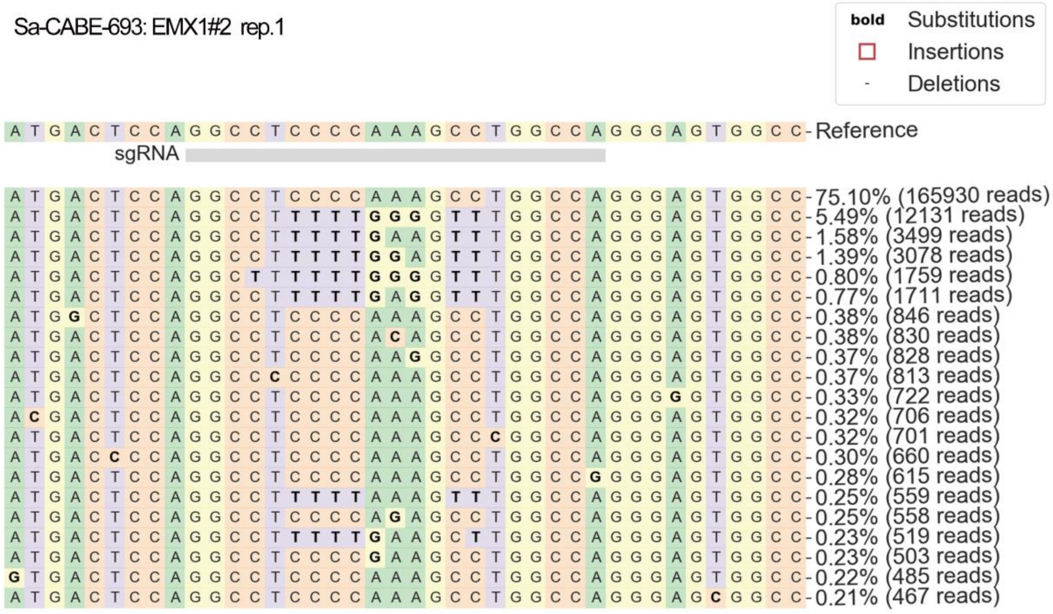

Figure S11.

Allele frequencies following treatment with Sa-CABE-693 at EMX1#2 genomic loci.

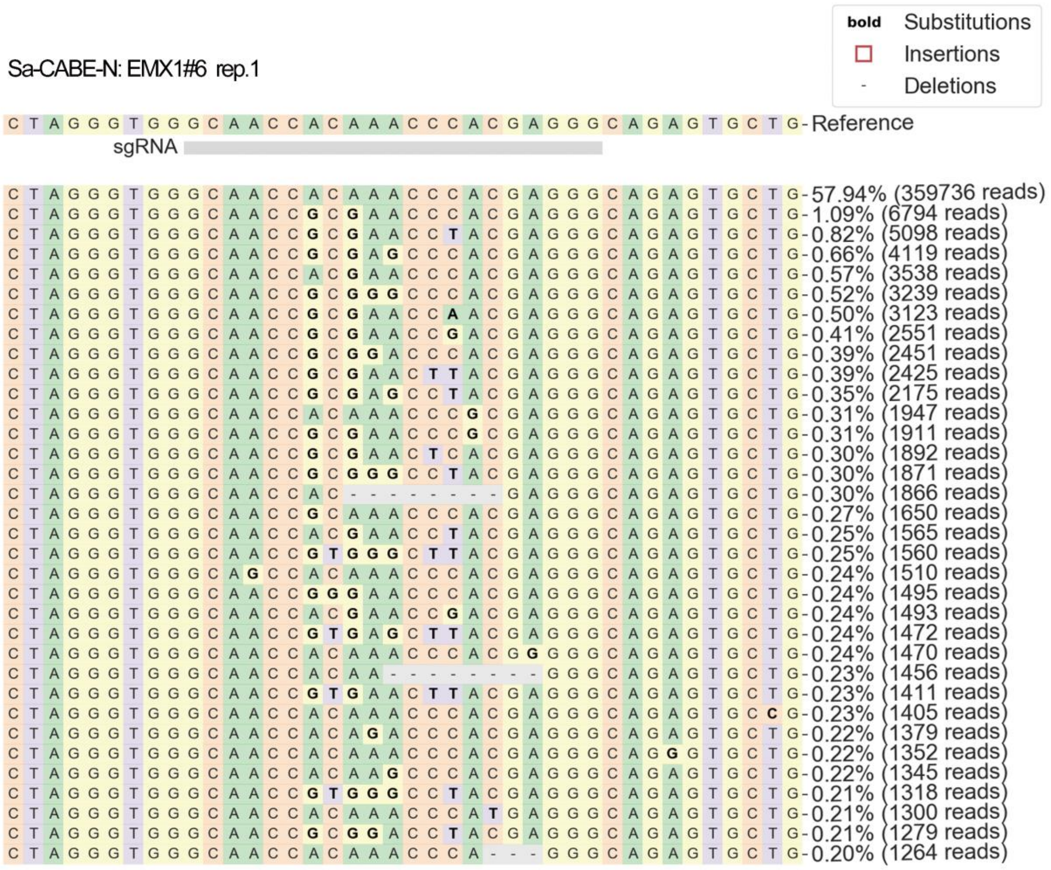

Figure S12.

Allele frequencies following treatment with Sa-CABE-N at EMX1#6 genomic loci.

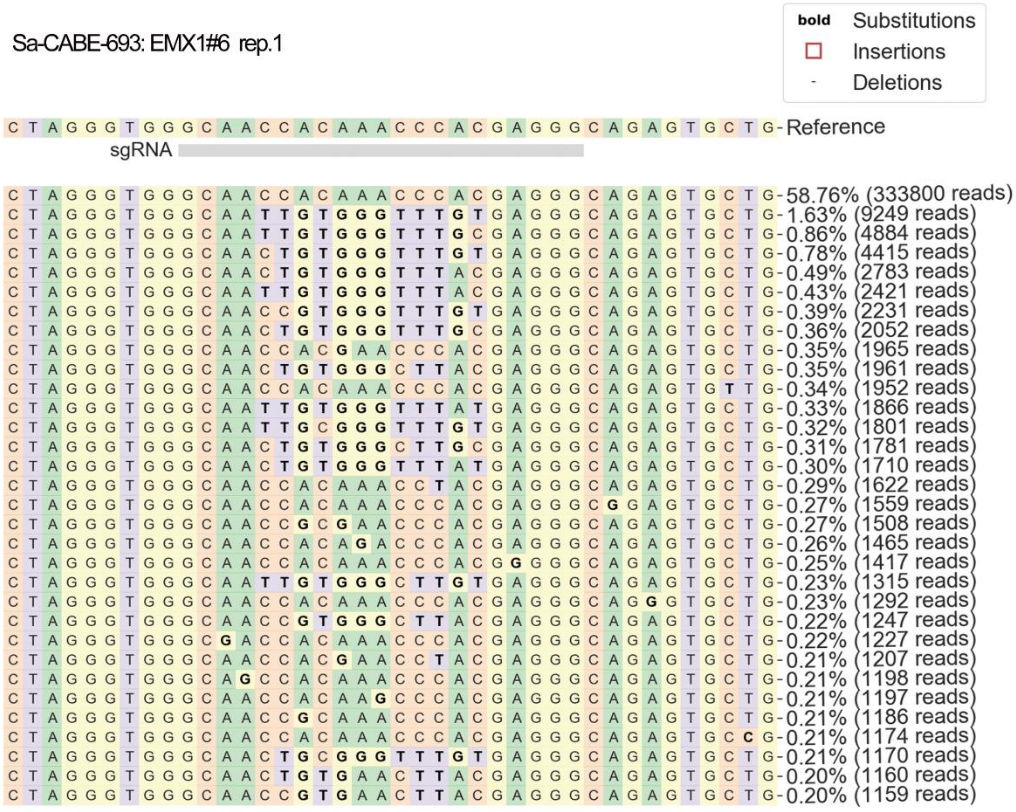

Figure S13.

Allele frequencies following treatment with Sa-CABE-693 at EMX1#6 genomic loci.

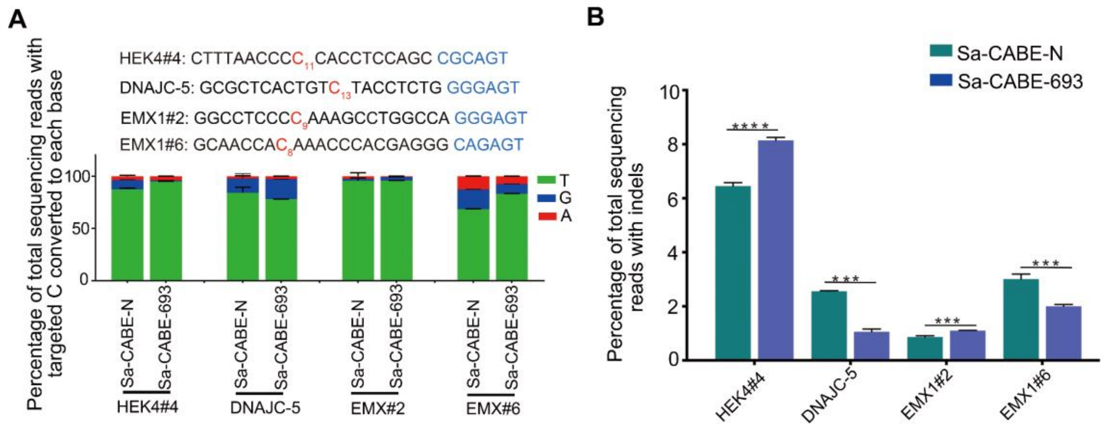

Figure S14.

Indels frequencies and product purity following treatment of HEK293T cells with Sa-CABE-N and Sa-CABE-693 at all 4 genomic loci. A. The product distribution among edited DNA sequencing reads (reads in which the target C is converted) is shown for Sa-CABE-N and Sa-CABE-693. The position that has C to R conversion is indicated in red. Each experiment was repeated three times, data are represented as mean ± SD. **B.** Comparison of indels frequency produced by Sa-CABE-N and Sa-CABE-693 at 4 endogenous human genomic loci. Each experiment was repeated three times, data are represented as mean ± SD. Asterisks indicate statistically significant differences in editing efficiencies observed between Sa-CABE-N and Sa-CABE-693 at each site. ($P \geq 0.05$, *$P < 0.05$, **$P < 0.01$, ***$P < 0.001$, ****$P < 0.0001$ by two-tailed Student's t test). Editing efficiencies were measured by HTS.

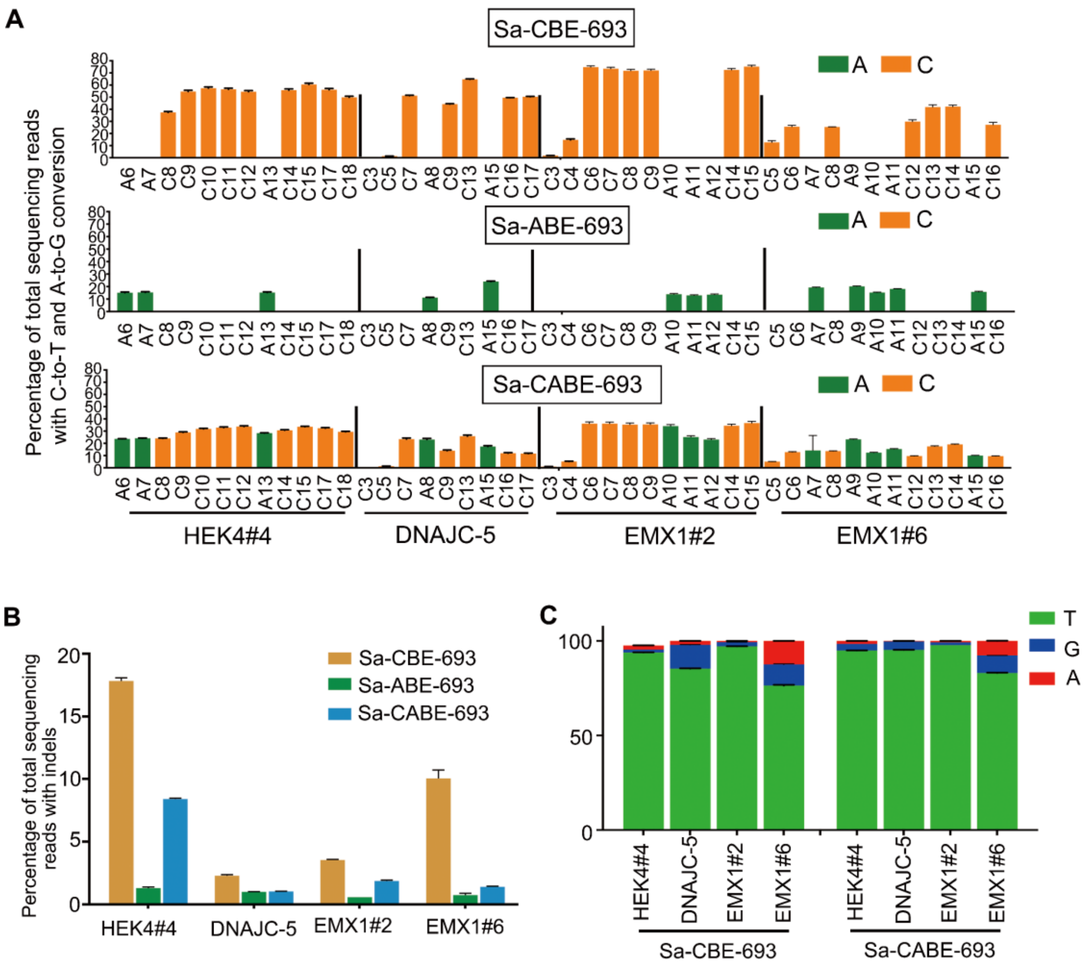

Figure S15. Comparison of base editing by Sa-CBE-693, Sa-ABE-693 and Sa-CABE-693.

A. Comparison of the C-to-T and A-to-G base-editing frequencies produced by Sa-CBE-693, Sa-ABE-693 and Sa-CABE-693 at 4 endogenous human genomic loci. **B.** Comparison of indels frequency produced by indicated editors. **C.** The product distribution among edited DNA sequencing reads (reads in which the target C is converted) is shown for Sa-CBE-693 and Sa-CABE-693. The position that has C to R conversion is indicated in red. Values and error bars reflect the mean ± SD of 3 independent experiments. Editing efficiencies were measured by HTS.

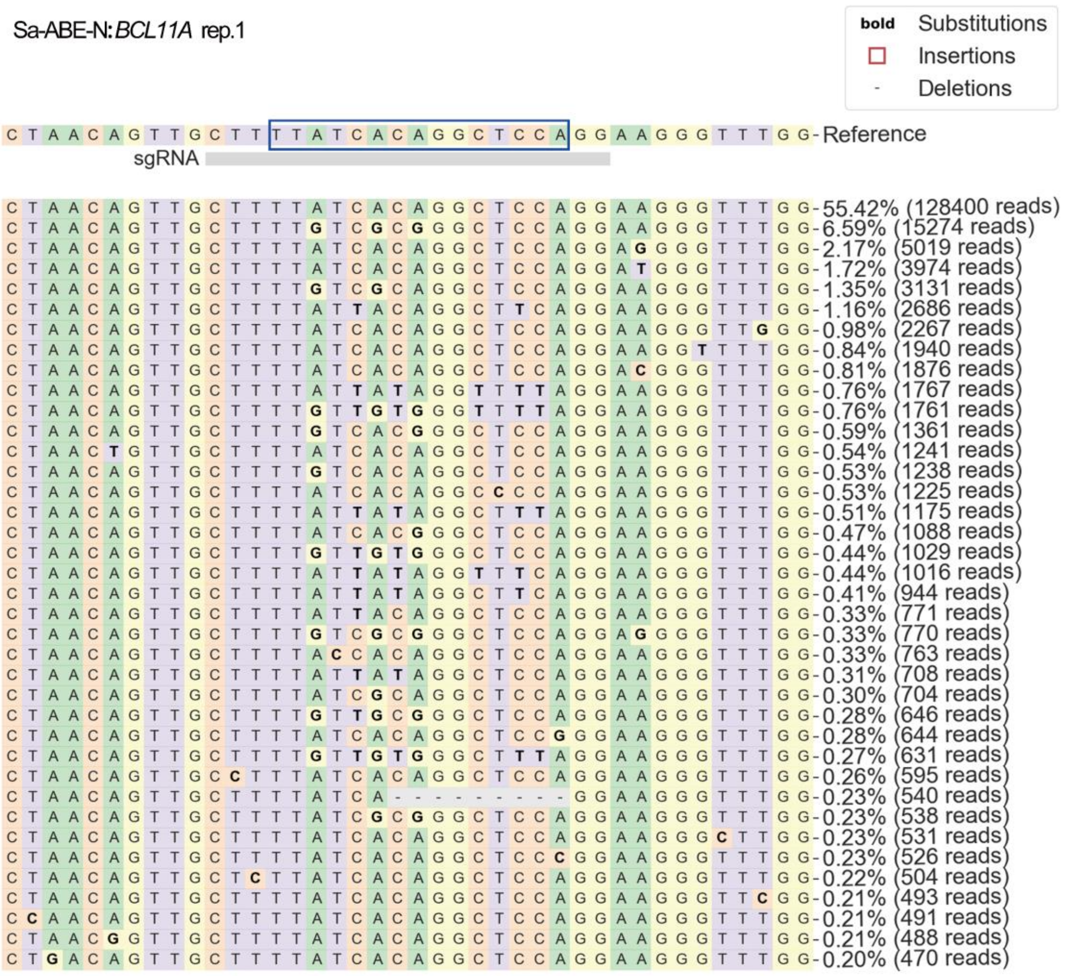

Figure S16.

Allele frequencies following treatment with Sa-ABE-N at a *BCL11A* enhancer. Target site is marked with a gray line. Consensus bases within a 15 bp fragment (TGN7-9WGATAR, where W=A or T and R=G or A) was framed with blue box. The percentile of unique sequencing read that represents a representative experiment from three independent experiments is listed on the right.

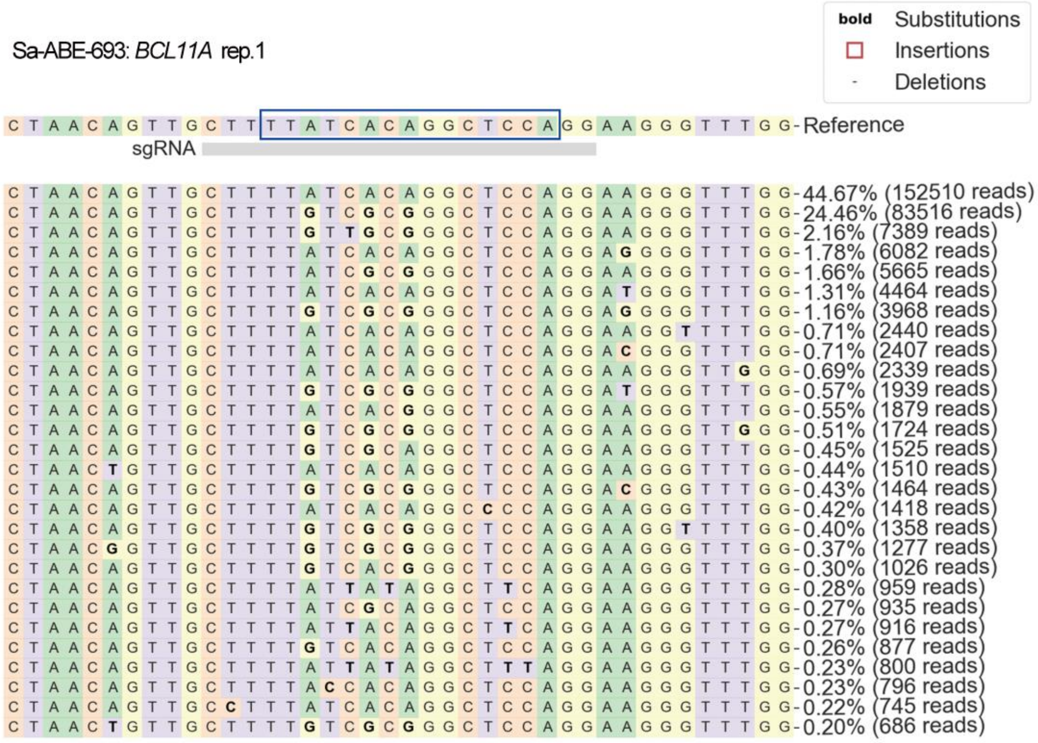

Figure S17.

Allele frequencies following treatment with Sa-ABE-693 at *BCL11A* enhancer.

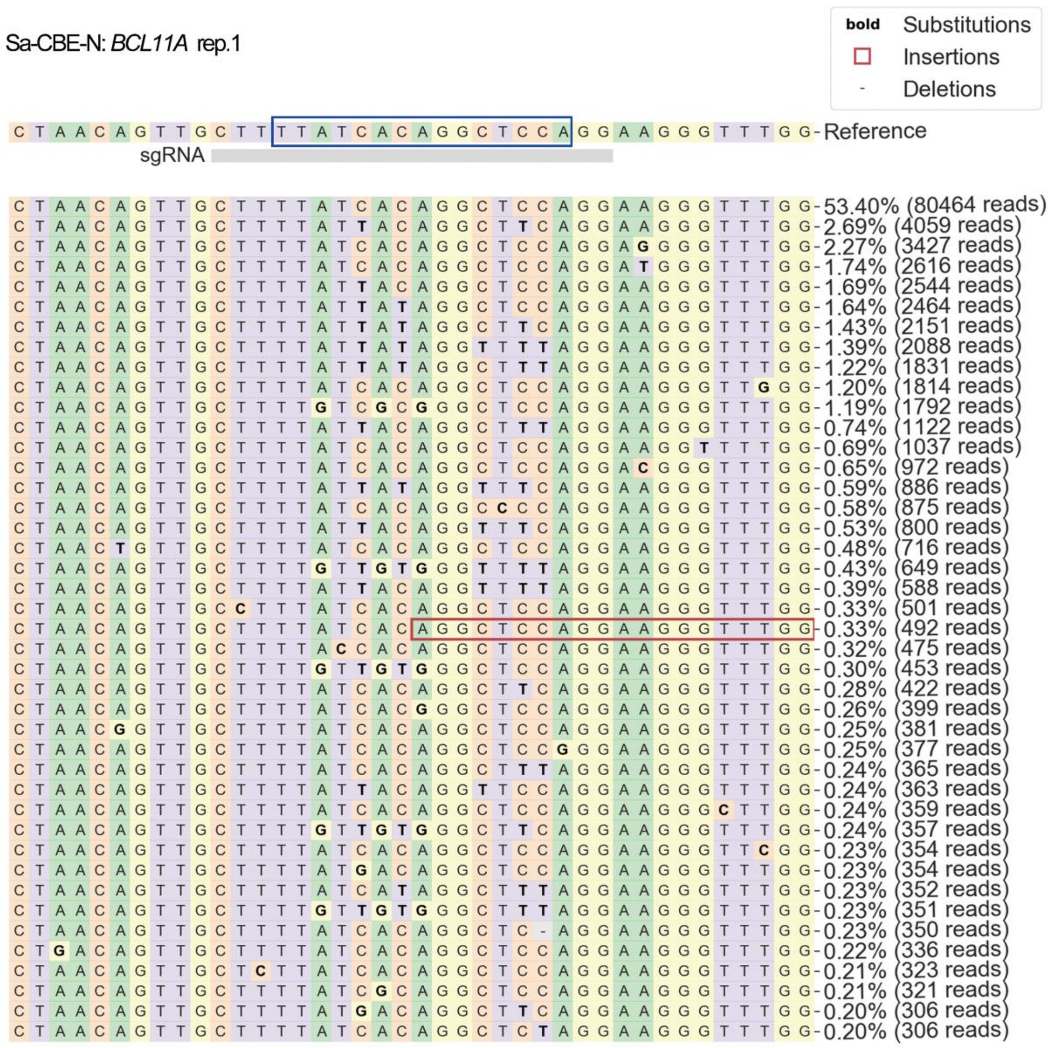

Figure S18.

Allele frequencies following treatment with Sa-CBE-N at *BCL11A* enhancer.

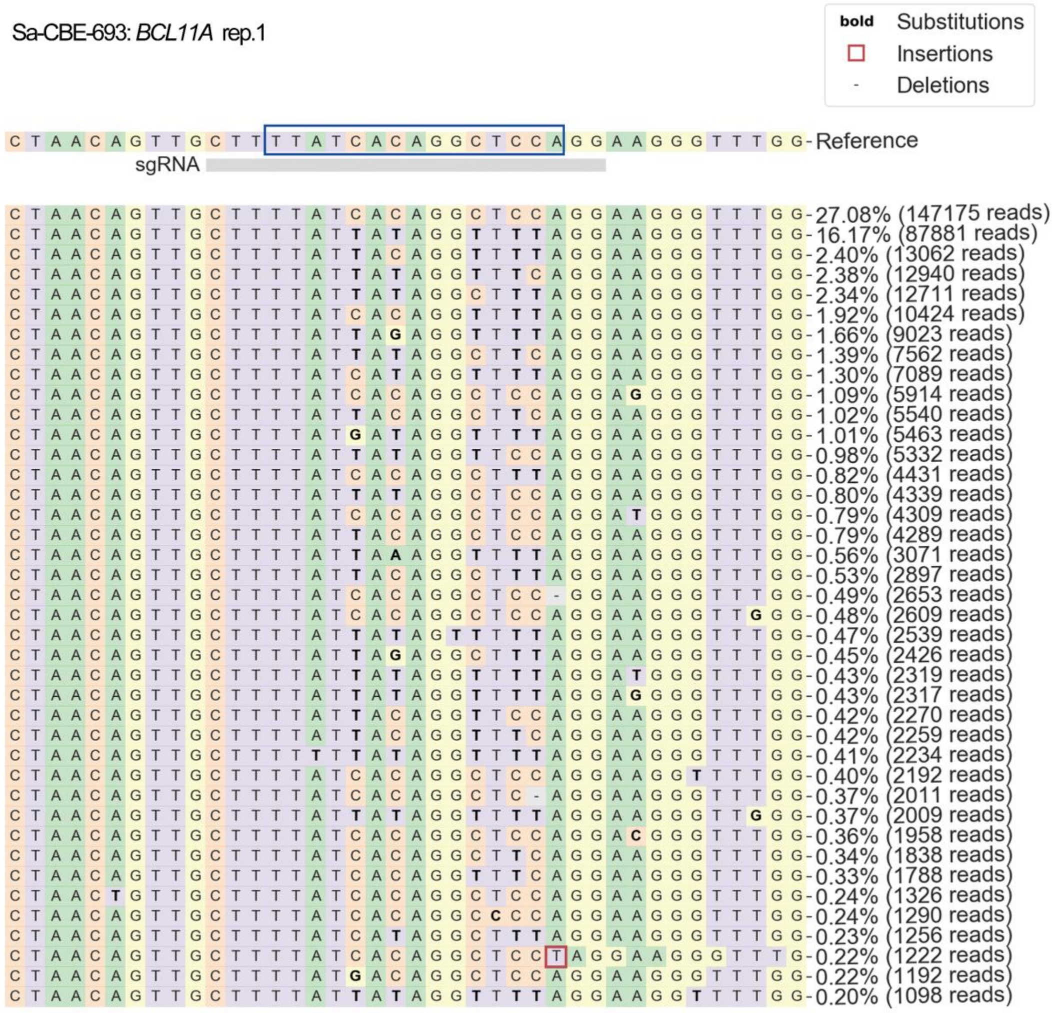

Figure S19.

Allele frequencies following treatment with Sa-CBE-693 at *BCL11A* enhancer.

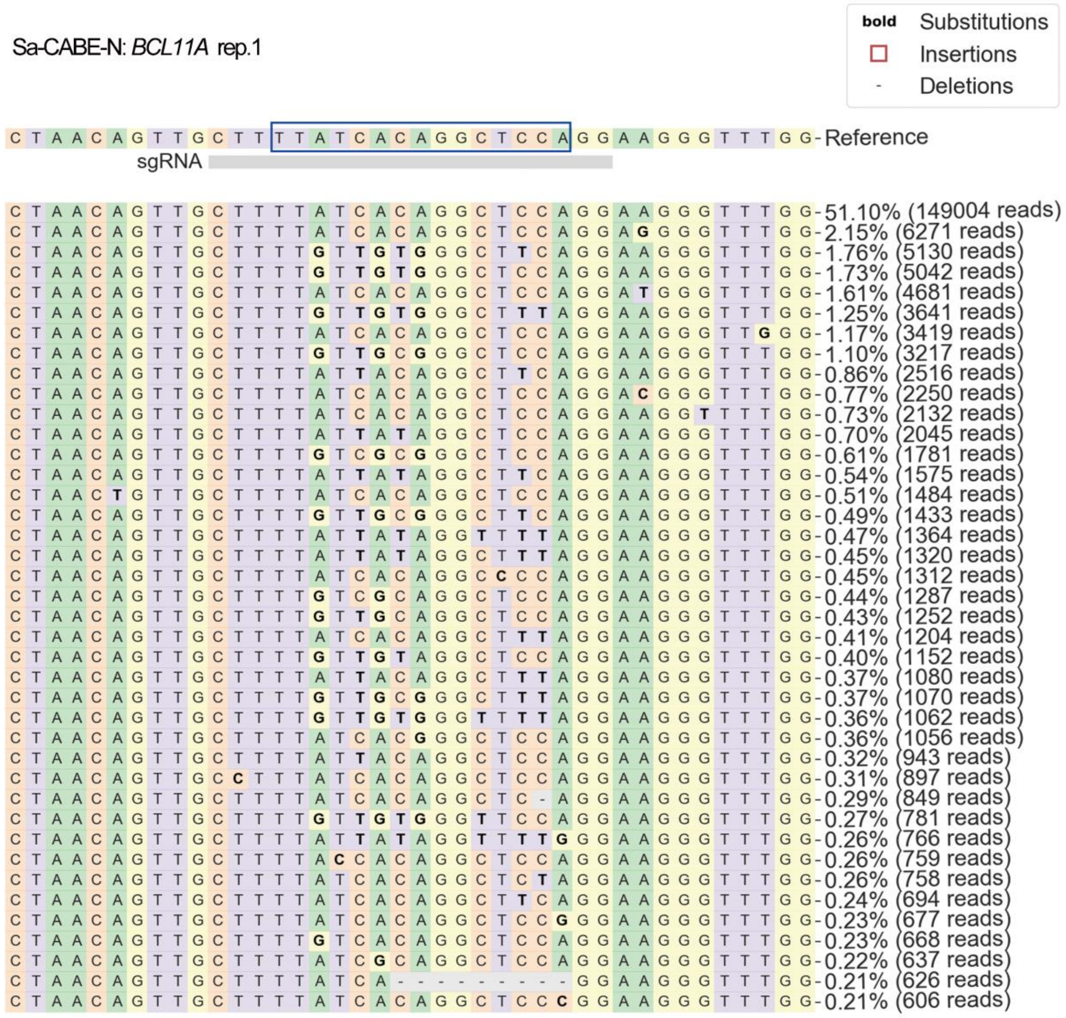

Figure S20.

Allele frequencies following treatment with Sa-CABE-N at *BCL11A* enhancer.

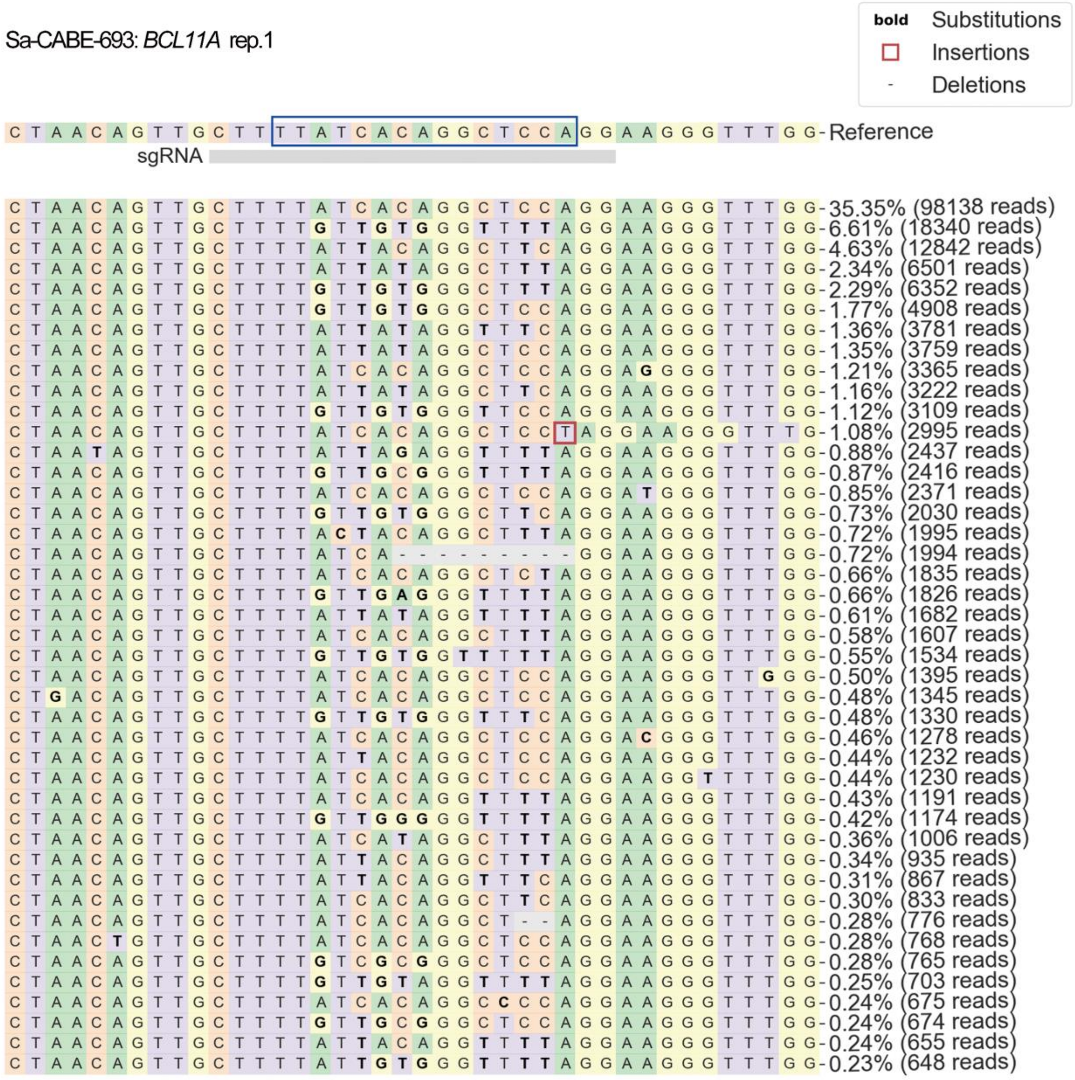

Figure S21.

Allele frequencies following treatment with Sa-CABE-693 at *BCL11A* enhancer.

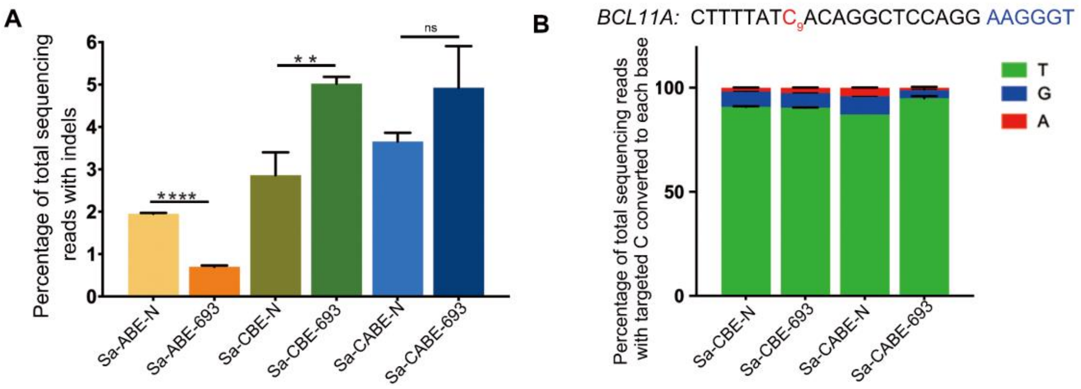

Figure S22.

Indels frequencies and product purity following treatment of HEK293T cells with Sa-BEs-N and inlaid Sa-BEs-693 at *BCL11A* editing site. A. Indel frequencies following treatment of HEK293T cells with Sa-ABE-N, Sa-ABE-693, Sa-CBE-N, Sa-CBE-693, Sa-CABE-N and Sa-CABE-693. Each experiment was repeated three times, data are represented as mean ± SD. Asterisks indicate statistically significant differences in editing efficiencies observed among Sa-ABE-N, Sa-ABE-693, Sa-CBE-N, Sa-CBE-693, Sa-CABE-N and Sa-CABE-693 ($P \geq 0.05$, *$P < 0.05$, **$P < 0.01$, ***$P < 0.001$, ****$P < 0.0001$ by two-tailed Student's t test). Editing efficiencies were measured by HTS. **B.** The product distribution among edited DNA sequencing reads (reads in which the target C is converted) is shown for Sa-CBE-N, Sa-CBE-693, Sa-CABE-N and Sa-CABE-693. C9 that has C to R conversion is labelled in red. Each experiment was repeated three times, data are represented as mean ± SD.

Table S1.

SgRNAs and Oligos sequence used for CBE editing window

SgRNA	Target sequence	Oligo F	Oligo R	Reference
EMX1#1	CCTCCCTCCCTGGCCCAGGT	CACCGCCTCCCTCCCTGGCCCAGGT	AAACACCTGGGCCAGGGAGGGAGGC	[1]
EMX1#2	GGCCTCCCCAAAGCCTGGCCA	CACCGGCCTCCCCAAAGCCTGGCCA	AAACTGGCCAGGCTTTGGGGAGGCC	[1]
HEK4#4	CTTTAACCCCCACCTCCAGC	CACCGCTTTAACCCCCACCTCCAGC	AAACGCTGGAGGTGGGGGTTAAAGC	[2]
DNMJC5-5	GCGCTCACTGTCTACCTCTG	CACCGCGCTCACTGTCTACCTCTG	AAACCAGAGGTAGACAGTGAGCGC	This study
Sa-Site29	GGGCCCTGCCTTCCTCACCT	CACCGGGCCCTGCCTTCCTCACCT	AAACAGGTGAGGAAGGCAGGGCCC	This study
EMX1#6	GCAACCACAAACCCACGAGGG	CACCGCAACCACAAACCCACGAGGG	AAACCCCTCGTGGGTTTGTGGTTGC	[1]
RUNX1#14	GTACTCACCTCTCATGAAGCACT	CACCGTACTCACCTCTCATGAAGCACT	AAACAGTGCTTCATGAGAGGTGAGTAC	[1]
FANCF#2 （Sasite6）	GATGTTCCAATCAGTACGCA	CACCGATGTTCCAATCAGTACGCA	AAACTGCGTACTGATTGGAACATC	[3]
HEK3-1 (Sasite5)	TCTGCTTCTCCAGCCCTGGC	CACCGTCTGCTTCTCCAGCCCTGGC	AAACGCCAGGGCTGGAGAAGCAGAC	[3]
HEK3-2	ACGTGCTCAGTCTGGGCCCC	CACCGACGTGCTCAGTCTGGGCCCC	AAACGGGGCCCAGACTGAGCACGTC	[3]
VEGFA11	GCTCCATTCACCCAGCTTCCC	CACCGCTCCATTCACCCAGCTTCCC	AAACGGGAAGCTGGGTGAATGGAGC	This study
HB5	CTAAGCCCAACACACATGCTG	CACCGCTAAGCCCAACACACATGCTG	AAACCAGCATGTGTGTTGGGCTTAGC	This study
HB2	GTTCTACCTTAACTATGCAGA	CACCGTTCTACCTTAACTATGCAGA	AAACTCTGCATAGTTAAGGTAGAAC	This study

HB3	TAGCCTGACAGGAGCACCCAT	CACCGTAGCCTGACAGGAGCACCCAT	AAACATGGGTGCTCCTGTCAGGCTAC	This study
AV3	ACCCAGCGAGTGAAGACGGCA	CACCGACCCAGCGAGTGAAGACGGCA	AAACTGCCGTCTTCACTCGCTGGGTC	This study
ZKSCAN1-On	CCAGACCTGGAGCTCCTTGGG	CACCGCCAGACCTGGAGCTCCTTGGG	AAACCCCAAGGAGCTCCAGGTCTGGC	[4]
Sa-SiteB	GacAGAGCCCCCCCTCAAAGA	CACCGacAGAGCCCCCCCTCAAAGA	CACCGacAGAGCCCCCCCTCAAAGA	This study
HBB-On	ACTCAAAGAACCTCTGGGTCC	CACCGACTCAAAGAACCTCTGGGTCC	AAACGGACCCAGAGGTTCTTTGAGTC	[4]
AC2	CGTAGGACTCTCTTCTCTGAC	CACCGCGTAGGACTCTCTTCTCTGAC	AAACGTCAGAGAAGAGAGTCCTACGC	This study
RS1-On	CATATCACAAGCCTCTGGGTT	CACCGCATATCACAAGCCTCTGGGTT	AAACAACCCAGAGGCTTGTGATATGC	[4]
AC3	ACCGGAGTCCATCACGATGCC	CACCGACCGGAGTCCATCACGATGCC	AAACGGCATCGTGATGGACTCCGGTC	This study
DNMT13B	TCCCCCATCCTGCCCCAGAGG	CACCGTCCCCCATCCTGCCCCAGAGG	AAACCCTCTGGGGCAGGATGGGGGAC	This study
V5	CCTGGACACTTCCCAAAGGAC	CACCGCCTGGACACTTCCCAAAGGAC	AAACGTCCTTTGGGAAGTGTCCAGGC	This study
PAH-1	GGGCACAGCGTTCAAGACTC	CACCGGGCACAGCGTTCAAGACTC	AAACGAGTCTTGAACGCTGTGCCC	This study
PAH-2	GGGCCGAGGTACTGTGGCAG	CACCGGGCCGAGGTACTGTGGCAG	AAACGGGCCGAGGTACTGTGGCAG	This study

Table S2.

SgRNAs and Oligos sequence used for ABE editing window

SgRNA	Target sequence	Oligo F	Oligo R	Reference
EMX1#6	GCAACCACAAACCCACGAGGG	CACCGCAACCACAAACCCACGAGGG	AAACCCCTCGTGGGTTTGTGGTTGC	[1]
RUNX1#14	GTACTCACCTCTCATGAAGCACT	CACCGTACTCACCTCTCATGAAGCACT	AAACAGTGCTTCATGAGAGGTGAGTAC	[1]
FANCF#2	GATGTTCCAATCAGTACGCA	CACCGATGTTCCAATCAGTACGCA	AAACTGCGTACTGATTGGAACATC	[3]
Site1	CTGAATAGCTGCAAACAAGTG	CACCGCTGAATAGCTGCAAACAAGTG	AAACCACTTGTTTGCAGCTATTCAGC	[5]
Site6	GGGAGCTAGACAAAATGGAGT	CACCGGGAGCTAGACAAAATGGAGT	AAACACTCCATTTTGTCTAGCTCCC	[5]
Site11	GCTGTTGCATGAGGAAAGGGAC	CACCGCTGTTGCATGAGGAAAGGGAC	AAACGTCCCTTTCCTCATGCAACAGC	[5]
AC2-2	AAGGAGACTCAGGTCAGAGAA	CACCGAAGGAGACTCAGGTCAGAGAA	AAACTTCTCTGACCTGAGTCTCCTTC	This study
AV4	GGGGCTCAACATCGGAAGAGG	CACCGGGGCTCAACATCGGAAGAGG	AAACCCTCTTCCGATGTTGAGCCCC	This study
HB5	CTAAGCCCAACACACATGCTG	CACCGCTAAGCCCAACACACATGCTG	AAACCAGCATGTGTGTTGGGCTTAGC	This study
AV3	ACCCAGCGAGTGAAGACGGCA	CACCGACCCAGCGAGTGAAGACGGCA	AAACTGCCGTCTTCACTCGCTGGGTC	This study
Sa-Site4	AAGAGAATAGACTGTAGGGAAACCAGT	CACCGAAGAGAATAGACTGTAGGGAA	AAACTTCCCTACAGTCTATTCTCTTC	This study
HB2	GTTCTACCTTAACTATGCAGA	CACCGTTCTACCTTAACTATGCAGA	AAACTCTGCATAGTTAAGGTAGAAC	This study
V1	AGAGGGAATGGGCTTTGGAAA	CACCGAGAGGGAATGGGCTTTGGAAA	AAACTTTCCAAAGCCCATTCCCTCTC	This study

ABE-S1	CTGAATAGCTGCAAAC AAGTG	CACCGCTGAATAGC TGCAAACAAGTG	AAACCACTTGTTTG CAGCTATTCAGC	[5]
ABE-S7	AAGAATACTAAGCAT AGACTC	CACCGAAGAATACT AAGCATAGACTC	AAACGAGTCTATGC TTAGTATTCTTC	[5]
ABE-S8	TGGGCTTTAGGAACAG GGGTA	CACCGTGGGCTTTAG GAACAGGGGTA	AAACTACCCCTGTT CCTAAAGCCCAC	[5]
ABE-S9	AACAACAAAACGGAC AGTGAA	CACCGAACAACAAA ACGGACAGTGAA	AAACTTCACTGTCC GTTTTGTTGTTC	[5]
ABE-S11	GGGAGCTAGACAAAA TGGAGT	CACCGGGAGCTAGA CAAAATGGAGT	AAACACTCCATTTT GTCTAGCTCCC	[5]
ABE-S19	GTGTCAGGTAATGTGC TAAACA	CACCGTGTCAGGTA ATGTGCTAAACA	AAACTGTTTAGCAC ATTACCTGACAC	[5]
ABE-S21	GCTGTTGCATGAGGAA AGGGAC	CACCGCTGTTGCATG AGGAAAGGGAC	AAACGTCCCTTTCC TCATGCAACAGC	[5]
ABE-S27	GGGAGCTCAAGCCTG ATTCCAA	CACCGGGAGCTCAA GCCTGATTCCAA	AAACTTGGAATCAG GCTTGAGCTCCC	[5]

Table S3.

SgRNAs and Oligos sequence used for testing DNA off-target editing

SgRNA	Target sequence	Oligo F	Oligo R	Reference
HEK4	GGCACTGCGGCTGGAGGTGG	CACCGGCACTGCGGCTGGAGGTGG	AAACCCACCTCCAGCCGCAGTGCC	[3]
SiteB	ACAGAGCCCCCCCTCAAAGAG	CACCGACAGAGCCCCCCCTCAAAGAG	AAACCTCTTTGAGGGGGGGCTCTGTC	[6]
PPP1R12C site5	GCTGACTCAGAGACCCTGAG	CACCGCTGACTCAGAGACCCTGAG	AAACCTCAGGGTCTCTGAGTCAGC	[7]
FANCF	GGAATCCCTTCTGCAGCACC	CACCGGAATCCCTTCTGCAGCACC	AAACGGTGCTGCAGAAGGGATTCC	[6]
FANCF#2	GATGTTCCAATCAGTACGCA	CACCGATGTTCCAATCAGTACGCA	AAACTGCGTACTGATTGGAACATC	[3]
HEK3	GGCCCAGACTGAGCACGTGA	CACCGGCCCAGACTGAGCACGTGA	AAACTCACGTGCTCAGTCTGGGCC	[8]
Site29	GTTCACACCCATGACGAACA	CACCGTTCACACCCATGACGAACA	AAACTGTTCGTCATGGGTGTGAAC	[9]
Site1	CTGAATAGCTGCAAACAAGTG	CACCGCTGAATAGCTGCAAACAAGTG	AAACCACTTGTTTGCAGCTATTCAGC	[5]

Table S4.

Primers used to amplify each target regions for Sanger sequencing

Target site	Chromosome	Forward primer	Reverse primer
DNAJC5-5	Chr20	CTGTCTGTGCACGTGGCAA	AGCTGTGACCAGTTCAACGC
HEK4	Chr20	CAGCGAGGTCAAAGTCACC	TCCTTTCAACCCGAACGGAG
FANCF#2	Chr11	ATGACTGGCATCATCTCGCA	GGTGCTGACGTAGGTAGTGC
HEK3-1	Chr9	AGAATGGGTCACAGTGGCA	TAGGAAAAGCTGTCCTGCGA
EMX1#1	Chr2	CCCTATGTAGCCTCAGTCTT	GATGTGATGGGAGCCCTTCT
EMX1#2	Chr2	CGAGGAGAAGGCCAAGTGG	CCAATGACTAGGGTGGGCAA
EMX1#6	Chr2	AGCAGAAGAAGAAGGGCTC	ACTCCAGGCCTCCCCAAA
HEK4#4	Chr20	GGCTCCTTTCAACCCGAACG	GGCTGGGTGGAAGGAA
RUNX1#14	Chr21	CATCTCTGCACCGAGGTGAA	AGAAATCATTGAGTCCC
VEGFA11	Chr5	GGAACAAGGGCCTCTGTCTG	GCCGTTCCCTCTTTGCTAGG
HB5	Chr9	AGCAAAGCACCCTATAAACA	TGCCCTGACTTTTATGCCACT
HB2	Chr9	AGCAAAGCACCCTATAAACA	TGCCCTGACTTTTATGCCACT
HB3	Chr9	AGCAAAGCACCCTATAAACA	TGCCCTGACTTTTATGCCACT
AV3	Chr19	ATCCTCTCTGGCTCCATCGT	GATGCTCTTTCCGGAGCACT
ZKSCAN1-On	Chr7	GTGAGCACAGACCCTGTTTG	TGGCTCCAGTTTCAACGTCT
Sa-SiteB	Chr20	GCCTGGAGGGAAATCTTAGG	GGGCTTCACTGAGTCTCCAC
HBB-On	Chr11	AAGGTGCCCTTGAGGTTGTC	AGGGTTGGCCAATCTACTCC
AC2	Chr7	GAGGCGTACAGGGATAGCAC	GCTCAGGGCTTCTTGTCCTT
RS1-On	ChrX	AGCCCACATACTGCTC	CGTTGAAGACACAGCTGTA
AC3	Chr7	GGGTAACCCTCATGTCAGGC	CCAAGACCCCAGCACACTTA
DNMT13B	Chr20	AGGCTTTAGCAGCTGGTGTC	TAGCTCCCTATTCCCATGCCTA
V5	Chr6	AGACTCCACAGTGCATACG	GAGCCGTTCCCTCTTTGCTA
Site1	Chr5	ACCTGGCTGAGCTAACTGTG	GAAGCCAGTGGAATACAAA
Site6	Chr15	TGCTTCCTCCATGGAAAAAT	ATCGCTGACTCCAACCACCT
Site11	Chr1	ATGAATAGTTTAAGCAAGGC	CAGTGCCCCAAATTATTCAC

AC2-2	Chr7	GAGGCGTACAGGGATAGCAC	GCTCAGGGCTTCTTGTCCTT
AV4	Chr19	AGCGTTAGAGGGCAGAGTTC	ACTCTCTTCCGCATTGGAGTC
Sa-Site4	Chr19	CACTGTGTTAGCCAGGATGG	GAGCTGTCCAGAGGTGTTAAG
V1	Chr6	CCAGATGAGGGCTCCAGATG	GAAAGTGAGGTTACGTGCGG
ABE-S1	Chr5	TTCCCACGTATTGCACTGCC	GGGGAAAAATTGTCCAGCCC
ABE-S7	Chr4	GTGAGGGGCTCATGAACAGG	GCCACCTGAGACACATA
ABE-S8	Chr1	CCGACTCCGAAGACAGTCAG	CCTTCTACGGCAGAAACCACA
ABE-S9	Chr22	CACCTTTGGCCCAATGACAC	AGGATGAGCCTAGGGATTGGA
ABE-S11	Chr15	TAGGCTTAGGCCAACACAGC	TGTTGTCGCTTCTGCTCCAT
ABE-S19	Chr19	TCTTTGCTCCAGATTTCCCTTCA	ATCCTTGCACTGAGACCGTG
ABE-S21	Chr1	CCGACTCCGAAGACAGTCAG	CCTTCTACGGCAGAAACCACA
ABE-S27	Chr6	AAAATTGTCCACCTGGCACG	CTTCCCTCCTCTGCGTGAAT
HEK3-2	Chr9	CAGTATCCCGGTGCAGGAGC	AACGCCCATGCAATTAGTCT
Sa-Site29	Chr12	AGCAGTTGGTGGTGCAGGA	GGGAGCCAAAAGGGTCATCA
Site29	Chr12	AGCAGTTGGTGGTGCAGGA	GGGAGCCAAAAGGGTCATCA
FANCF	Chr11	GGAGACGTTCATGACTGGCA	GGGCCTGGAAGTTCGCTAAT
Sa-SiteB	Chr20	CCTGGAGGGAAATCTTAGGCA	GGGCTTCACTGAGTCTCCAC
SiteB	Chr20	CCTGGAGGGAAATCTTAGGCA	GGGCTTCACTGAGTCTCCAC
PPP1R12C site5	Chr19	CCCGGCCATGGTTTTAT	GACTTGCCCAGAGCTCTT
HEK3	Chr2	GTCTATTTCTGCTGCAAGTAAGCA	AGCCCCTGTCTAGGAAAAGC
BCL11A	Chr2	GGCCAGAAAAGAGATATGGC	AAACGGCCACCGATGGA
PAH-1		TGTAAAACGACGGCCAGT	CAGGAAACAGCTATGAC
PAH-2		TGTAAAACGACGGCCAGT	CAGGAAACAGCTATGAC

Table S5.

Sa-CBE HTS Primers used to amplify each target

Sample name	Primer name	Sequence
Sa-CBE-HEK3-2-N	HEK3-2-1-Fwd	ACACAGTGCACATACTAGCCCCTGTCT
	HEK3-2-1-Rev	AGCATGCATTTGTAGGCTTGAT
Sa-CBE-HEK3-2-125	HEK3-2-2-Fwd	ACTGACTGCACATACTAGCCCCTGTCT
	HEK3-2-2-Rev	AGCATGCATTTGTAGGCTTGAT
Sa-CBE-HEK3-2-269	HEK3-2-3-Fwd	TCAGAGAGCACATACTAGCCCCTGTCT
	HEK3-2-3-Rev	AGCATGCATTTGTAGGCTTGAT
Sa-CBE-HEK3-2-593	HEK3-2-4-Fwd	TGTCAGTGCACATACTAGCCCCTGTCT
	HEK3-2-4-Rev	AGCATGCATTTGTAGGCTTGAT
Sa-CBE-HEK3-2-693	HEK3-2-5-Fwd	CATCAGAGCACATACTAGCCCCTGTCT
	HEK3-2-5-Rev	AGCATGCATTTGTAGGCTTGAT
Sa-CBE-FANCF#2-N	FANCF#2-1-Fwd	ACAGCATCCCAGAGTCAAGGAACACGG
	FANCF#2-1-Rev	CTCGGAAAAGCGATCCAGGT
Sa-CBE-FANCF#2-125	FANCF#2-2-Fwd	ACTTGCACCCAGAGTCAAGGAACACGG
	FANCF#2-2-Rev	CTCGGAAAAGCGATCCAGGT
Sa-CBE-FANCF#2-269	FANCF#2-3-Fwd	ACGTTCTCCCAGAGTCAAGGAACACGG
	FANCF#2-3-Rev	CTCGGAAAAGCGATCCAGGT
Sa-CBE-FANCF#2-593	FANCF#2-4-Fwd	AGATCACCCCAGAGTCAAGGAACACGG
	FANCF#2-4-Rev	CTCGGAAAAGCGATCCAGGT
Sa-CBE-FANCF#2-693	FANCF#2-5-Fwd	AGAGCTACCCAGAGTCAAGGAACACGG
	FANCF#2-5-Rev	CTCGGAAAAGCGATCCAGGT
Sa-CBE-EMX1#1-N	EMX1#1-1-Fwd	CAGATCATCAGTCTTCCCATCAGGCTC
	EMX1#1-1-Rev	CCCTTCTTCTTCTGCTCGGA
Sa-CBE-EMX1#1-125	EMX1#1-2-Fwd	CAGTACTTCAGTCTTCCCATCAGGCTC
	EMX1#1-2-Rev	CCCTTCTTCTTCTGCTCGGA
Sa-CBE-EMX1#1-269	EMX1#1-3-Fwd	CTTGAGTTCAGTCTTCCCATCAGGCTC
	EMX1#1-3-Rev	CCCTTCTTCTTCTGCTCGGA

Sa-CBE-EMX1#1-593	EMX1#1-4-Fwd	CTCTCAGTCAGTCTTCCCATCAGGCTC
	EMX1#1-4-Rev	CCCTTCTTCTTCTGCTCGGA
Sa-CBE-EMX1#1-693	EMX1#1-5-Fwd	CTGTAGATCAGTCTTCCCATCAGGCTC
	EMX1#1-5-Rev	CCCTTCTTCTTCTGCTCGGA
Sa-CBE-HEK4#4-N	HEK4#4-1-Fwd	GACTCACTTTCAACCCGAACGGAGACAC
	HEK4#4-1-Rev	GTCCAAAGCAGGATGACAGG
Sa-CBE-HEK4#4-125	HEK4#4-2-Fwd	GTAGTCTTTTCAACCCGAACGGAGACAC
	HEK4#4-2-Rev	GTCCAAAGCAGGATGACAGG
Sa-CBE-HEK4#4-269	HEK4#4-3-Fwd	GTCGTGATTTCAACCCGAACGGAGACAC
	HEK4#4-3-Rev	GTCCAAAGCAGGATGACAGG
Sa-CBE-HEK4#4-593	HEK4#4-4-Fwd	GTGTCAATTTCAACCCGAACGGAGACAC
	HEK4#4-4-Rev	GTCCAAAGCAGGATGACAGG
Sa-CBE-HEK4#4-693	HEK4#4-5-Fwd	AAGAGCTTTTCAACCCGAACGGAGACAC
	HEK4#4-5-Rev	GTCCAAAGCAGGATGACAGG
Sa-CBE-EMX1#6-N	EMX1#6-1-Fwd	GCCTCTACGAGCAGAAGAAGAAGGGCT
	EMX1#6-1-Rev	TTGTCCCTCTGTCAATGGCG
Sa-CBE-EMX1#6-125	EMX1#6-2-Fwd	AACCTAGCGAGCAGAAGAAGAAGGGCT
	EMX1#6-2-Rev	TTGTCCCTCTGTCAATGGCG
Sa-CBE-EMX1#6-269	EMX1#6-3-Fwd	ATTCTCGCGAGCAGAAGAAGAAGGGCT
	EMX1#6-3-Rev	TTGTCCCTCTGTCAATGGCG
Sa-CBE-EMX1#6-593	EMX1#6-4-Fwd	ATCCGGACGAGCAGAAGAAGAAGGGCT
	EMX1#6-4-Rev	TTGTCCCTCTGTCAATGGCG
Sa-CBE-EMX1#6-693	EMX1#6-5-Fwd	ATGCTTCCGAGCAGAAGAAGAAGGGCT
	EMX1#6-5-Rev	TTGTCCCTCTGTCAATGGCG
Sa-CBE-DNAJC-5-N	DNAJC-5-1-Fwd	ACAACTGAGCTCTGCCCTTGGTACTTTC
	DNAJC-5-1-Rev	CCACACTTGTCCACTTACCGA
Sa-CBE-DNAJC-5-125	DNAJC-5-2-Fwd	ACACGTAAGCTCTGCCCTTGGTACTTTC
	DNAJC-5-2-Rev	CCACACTTGTCCACTTACCGA

Sa-CBE-DNAJC-5-269	DNAJC-5-3-Fwd	ACTACGTAGCTCTGCCCTTGGTACTTTC
	DNAJC-5-3-Rev	CCACACTTGTCCACTTACCGA
Sa-CBE-DNAJC-5-593	DNAJC-5-4-Fwd	ACTCAAGAGCTCTGCCCTTGGTACTTTC
	DNAJC-5-4-Rev	CCACACTTGTCCACTTACCGA
Sa-CBE-DNAJC-5-693	DNAJC-5-5-Fwd	ACGTCTCAGCTCTGCCCTTGGTACTTTC
	DNAJC-5-5-Rev	CCACACTTGTCCACTTACCGA
Sa-CBE-Site29-N	Site29-1-Fwd	AGTACAGGCATTGCTGCAAAGAAAGAGGG
	Site29-1-Rev	TGATGCCCCCATGTTCGTCAT
Sa-CBE-Site29-125	Site29-2-Fwd	AGTCGTTGCATTGCTGCAAAGAAAGAGGG
	Site29-2-Rev	TGATGCCCCCATGTTCGTCAT
Sa-CBE-Site29-269	Site29-3-Fwd	AGCTCTGGCATTGCTGCAAAGAAAGAGGG
	Site29-3-Rev	TGATGCCCCCATGTTCGTCAT
Sa-CBE-Site29-593	Site29-4-Fwd	TACAGAGGCATTGCTGCAAAGAAAGAGGG
	Site29-4-Rev	TGATGCCCCCATGTTCGTCAT
Sa-CBE-Site29-693	Site29-5-Fwd	TAGCTCTGCATTGCTGCAAAGAAAGAGGG
	Site29-5-Rev	TGATGCCCCCATGTTCGTCAT
Sa-CBE-EMX1#2-N	EMX1#2-1-Fwd	GATTGCTAGCCCATTGCTTGTCCCTC
	EMX1#2-1-Rev	CTAGGGTGGGCAACCACAAA
Sa-CBE-EMX1#2-125	EMX1#2-2-Fwd	GACCATCAGCCCATTGCTTGTCCCTC
	EMX1#2-2-Rev	CTAGGGTGGGCAACCACAAA
Sa-CBE-EMX1#2-269	EMX1#2-3-Fwd	GAGTTCGAGCCCATTGCTTGTCCCTC
	EMX1#2-3-Rev	CTAGGGTGGGCAACCACAAA
Sa-CBE-EMX1#2-593	EMX1#2-4-Fwd	GTACTTGAGCCCATTGCTTGTCCCTC
	EMX1#2-4-Rev	CTAGGGTGGGCAACCACAAA
Sa-CBE-EMX1#2-693	EMX1#2-5-Fwd	GTTGCCAAGCCCATTGCTTGTCCCTC
	EMX1#2-5-Rev	CTAGGGTGGGCAACCACAAA
Sa-CBE-RUNX14-N	RUNX14-1-Fwd	TATGCGTCACCGAGGTGAAACAAGCTG
	RUNX14-1-Rev	CAGAAGAGGGTGCATTTTCAGG

Sa-CBE-RUNX14-125	RUNX14-2-Fwd	TACGACCCACCGAGGTGAAACAAGCTG
	RUNX14-2-Rev	CAGAAGAGGGTGCATTTTCAGG
Sa-CBE-RUNX14-269	RUNX14-3-Fwd	TAGGTTCCACCGAGGTGAAACAAGCTG
	RUNX14-3-Rev	CAGAAGAGGGTGCATTTTCAGG
Sa-CBE-RUNX14-593	RUNX14-4-Fwd	TTCGCAACACCGAGGTGAAACAAGCTG
	RUNX14-4-Rev	CAGAAGAGGGTGCATTTTCAGG
Sa-CBE-RUNX14-693	RUNX14-5-Fwd	TTGCCTACACCGAGGTGAAACAAGCTG
	RUNX14-5-Rev	CAGAAGAGGGTGCATTTTCAGG
Sa-CBE-HEK3-1-N	HEK3-1-1-Fwd	AGCATGAGGCATGGATGAGAGAAGCCT
	HEK3-1-1-Rev	TAGGAAAAGCTGTCCTGCGA
Sa-CBE-HEK3-1-125	HEK3-1-2-Fwd	AGGACTTGGCATGGATGAGAGAAGCCT
	HEK3-1-2-Rev	TAGGAAAAGCTGTCCTGCGA
Sa-CBE-HEK3-1-269	HEK3-1-3-Fwd	TAGTGCAGGCATGGATGAGAGAAGCCT
	HEK3-1-3-Rev	TAGGAAAAGCTGTCCTGCGA
Sa-CBE-HEK3-1-593	HEK3-1-4-Fwd	TTGACAGGGCATGGATGAGAGAAGCCT
	HEK3-1-4-Rev	TAGGAAAAGCTGTCCTGCGA
Sa-CBE-HEK3-1-693	HEK3-1-5-Fwd	TCACACGGGCATGGATGAGAGAAGCCT
	HEK3-1-5-Rev	TAGGAAAAGCTGTCCTGCGA
Sa-CBE-VEGFA11-N	VEGFA11-1-Fwd	TGCATACACCCCTGGCCTTCTCCC
	VEGFA11-1-Rev	GCCCATTCCCTCTTTAGCCA
Sa-CBE-VEGFA11-125	VEGFA11-2-Fwd	CATACGCACCCCTGGCCTTCTCCC
	VEGFA11-2-Rev	GCCCATTCCCTCTTTAGCCA
Sa-CBE-VEGFA11-269	VEGFA11-3-Fwd	CCGCATAACCCCTGGCCTTCTCCC
	VEGFA11-3-Rev	GCCCATTCCCTCTTTAGCCA
Sa-CBE-VEGFA11-593	VEGFA11-4-Fwd	CGTAACCACCCCTGGCCTTCTCCC
	VEGFA11-4-Rev	GCCCATTCCCTCTTTAGCCA
Sa-CBE-VEGFA11-693	VEGFA11-5-Fwd	GTAATGCACCCCTGGCCTTCTCCC
	VEGFA11-5-Rev	GCCCATTCCCTCTTTAGCCA
Sa-CBE-RS1-On-N	RS1-On-1-Fwd	CACACTGTACTGCTCCGGGTTAGAGCA

	RS1-On-1-Rev	CGCAAAGCAGATGGGTTTGT
Sa-CBE-RS1-On-125	RS1-On-2-Fwd	CAGATCATACTGCTCCGGGTTAGAGCA
	RS1-On-2-Rev	CGCAAAGCAGATGGGTTTGT
Sa-CBE-RS1-On-269	RS1-On-3-Fwd	CAGTACTTACTGCTCCGGGTTAGAGCA
	RS1-On-3-Rev	CGCAAAGCAGATGGGTTTGT
Sa-CBE-RS1-On-593	RS1-On-4-Fwd	CTTGAGTTACTGCTCCGGGTTAGAGCA
	RS1-On-4-Rev	CGCAAAGCAGATGGGTTTGT
Sa-CBE-RS1-On-693	RS1-On-5-Fwd	CTCTCAGTACTGCTCCGGGTTAGAGCA
	RS1-On-5-Rev	CGCAAAGCAGATGGGTTTGT
Sa- CBE-DNMT13B-N	DNMT13B-1-Fwd	TCAGTACACTCAGTGAAGCCCACTCAT
	DNMT13B-1-Rev	GGATACAGCTGCAGAACACAC
Sa- CBE-DNMT13B-125	DNMT13B-2-Fwd	TCTCGAAACTCAGTGAAGCCCACTCAT
	DNMT13B-2-Rev	GGATACAGCTGCAGAACACAC
Sa- CBE-DNMT13B-269	DNMT13B-3-Fwd	TCCTGGAACTCAGTGAAGCCCACTCAT
	DNMT13B-3-Rev	GGATACAGCTGCAGAACACAC
Sa- CBE-DNMT13B-593	DNMT13B-4-Fwd	TGATCGTACTCAGTGAAGCCCACTCAT
	DNMT13B-4-Rev	GGATACAGCTGCAGAACACAC
Sa- CBE-DNMT13B-693	DNMT13B-5-Fwd	TGTACTCACTCAGTGAAGCCCACTCAT
	DNMT13B-5-Rev	GGATACAGCTGCAGAACACAC
Sa- CBE-V5-N	V5-1-Fwd	AGTCGTTGAACCACACAGCTTCCCGTT
	V5-1-Rev	TCCCTCTGACAATGTGCCATC
Sa- CBE-V5-125	V5-2-Fwd	AGCTCTGGAACCACACAGCTTCCCGTT
	V5-2-Rev	TCCCTCTGACAATGTGCCATC
Sa- CBE-V5-269	V5-3-Fwd	TACAGAGGAACCACACAGCTTCCCGTT
	V5-3-Rev	TCCCTCTGACAATGTGCCATC
Sa- CBE-V5-593	V5-4-Fwd	TAGCTCTGAACCACACAGCTTCCCGTT
	V5-4-Rev	TCCCTCTGACAATGTGCCATC
Sa- CBE-V5-693	V5-5-Fwd	TCAAGCTGAACCACACAGCTTCCCGTT
	V5-5-Rev	TCCCTCTGACAATGTGCCATC

Table S6.

Sa-ABE HTS Primers used to amplify each target

Sample name	Primer name	Sequence
Sa-ABE-EMX1#6-N	EMX1#6-1-Fwd	GCCTCTACGAGCAGAAGAAGAAGGGCT
	EMX1#6-1-Rev	TTGTCCCTCTGTCAATGGCG
Sa-ABE-EMX1#6-125	EMX1#6-2-Fwd	AACCTAGCGAGCAGAAGAAGAAGGGCT
	EMX1#6-2-Rev	TTGTCCCTCTGTCAATGGCG
Sa-ABE-EMX1#6-269	EMX1#6-3-Fwd	ATTCTCGCGAGCAGAAGAAGAAGGGCT
	EMX1#6-3-Rev	TTGTCCCTCTGTCAATGGCG
Sa-ABE-EMX1#6-593	EMX1#6-4-Fwd	ATCCGGACGAGCAGAAGAAGAAGGGCT
	EMX1#6-4-Rev	TTGTCCCTCTGTCAATGGCG
Sa-ABE-EMX1#6-693	EMX1#6-5-Fwd	ATGCTTCCGAGCAGAAGAAGAAGGGCT
	EMX1#6-5-Rev	TTGTCCCTCTGTCAATGGCG
Sa-ABE-Site6-N	Site6-1-Fwd	CAGATCAAGATACAAGGACAGGCAGCATA
	Site6-1-Rev	AGAAATCGCTGACTCCAACCA
Sa-ABE-Site6-125	Site6-2-Fwd	CAGTACTAGATACAAGGACAGGCAGCATA
	Site6-2-Rev	AGAAATCGCTGACTCCAACCA
Sa-ABE-Site6-269	Site6-3-Fwd	CTTGAGTAGATACAAGGACAGGCAGCATA
	Site6-3-Rev	AGAAATCGCTGACTCCAACCA
Sa-ABE-Site6-593	Site6-4-Fwd	CTCTCAGAGATACAAGGACAGGCAGCATA
	Site6-4-Rev	AGAAATCGCTGACTCCAACCA
Sa-ABE-Site6-693	Site6-5-Fwd	CTGTAGAAGATACAAGGACAGGCAGCATA
	Site6-5-Rev	AGAAATCGCTGACTCCAACCA
Sa-ABE-Site1-N	Site1-1-Fwd	ACACAGTGCTAACTGTGACAGCATGTGG
	Site1-1-Rev	AAGAACACGTTTAAAGGGGGAA
Sa-ABE-Site1-125	Site1-2-Fwd	ACTGACTGCTAACTGTGACAGCATGTGG
	Site1-2-Rev	AAGAACACGTTTAAAGGGGGAA
Sa-ABE-Site1-269	Site1-3-Fwd	TCAGAGAGCTAACTGTGACAGCATGTGG
	Site1-3-Rev	AAGAACACGTTTAAAGGGGGAA

Sa-ABE-Site1-593	Site1-4-Fwd	TGTCAGTGCTAACTGTGACAGCATGTGG
	Site1-4-Rev	AAGAACACGTTTAAAGGGGGAA
Sa-ABE-Site1-693	Site1-5-Fwd	CATCAGAGCTAACTGTGACAGCATGTGG
	Site1-5-Rev	AAGAACACGTTTAAAGGGGGAA

Table S7.

Sa-CABE HTS Primers used to amplify each target

Sample name	Primer name	Sequence
Sa-CBE-HEK4#4-N	HEK4#4-1-Fwd	GACTCACTTTCAACCCGAACGGAGACAC
	HEK4#4-1-Rev	GTCCAAAGCAGGATGACAGG
Sa-CBE-HEK4#4-693	HEK4#4-2-Fwd	GTAGTCTTTTCAACCCGAACGGAGACAC
	HEK4#4-2-Rev	GTCCAAAGCAGGATGACAGG
Sa-ABE-HEK4#4-N	HEK4#4-3-Fwd	GTCGTGATTTCAACCCGAACGGAGACAC
	HEK4#4-3-Rev	GTCCAAAGCAGGATGACAGG
Sa-ABE-HEK4#4-693	HEK4#4-4-Fwd	GTGTCAATTTCAACCCGAACGGAGACAC
	HEK4#4-4-Rev	GTCCAAAGCAGGATGACAGG
Sa-CABE-HEK4#4-N	HEK4#4-5-Fwd	AAGAGCTTTTCAACCCGAACGGAGACAC
	HEK4#4-5-Rev	GTCCAAAGCAGGATGACAGG
Sa-CABE-HEK4#4-693	HEK4#4-6-Fwd	ATCTGCTTTTCAACCCGAACGGAGACAC
	HEK4#4-6-Rev	GTCCAAAGCAGGATGACAGG
Sa-CBE-DNAJC-5-N	DNAJC-5-1-Fwd	ACAACTGAGCTCTGCCCTTGGTACTTTC
	DNAJC-5-1-Rev	CCACACTTGTCCACTTACCGA
Sa-CBE-DNAJC-5-693	DNAJC-5-2-Fwd	ACACGTAAGCTCTGCCCTTGGTACTTTC
	DNAJC-5-2-Rev	CCACACTTGTCCACTTACCGA
Sa-ABE-DNAJC-5-N	DNAJC-5-3-Fwd	ACTACGTAGCTCTGCCCTTGGTACTTTC
	DNAJC-5-3-Rev	CCACACTTGTCCACTTACCGA
Sa-ABE-DNAJC-5-693	DNAJC-5-4-Fwd	ACTCAAGAGCTCTGCCCTTGGTACTTTC
	DNAJC-5-4-Rev	CCACACTTGTCCACTTACCGA
Sa-CABE-DNAJC-5-N	DNAJC-5-5-Fwd	ACGTCTCAGCTCTGCCCTTGGTACTTTC
	DNAJC-5-5-Rev	CCACACTTGTCCACTTACCGA
Sa-CABE-DNAJC-5-693	DNAJC-5-6-Fwd	AGACATGAGCTCTGCCCTTGGTACTTTC
	DNAJC-5-6-Rev	CCACACTTGTCCACTTACCGA
Sa-CBE-EMX1#2-N	EMX1#2-1-Fwd	GATTGCTAGCCCATTGCTTGTCCCTC
	EMX1#2-1-Rev	CTAGGGTGGGCAACCACAAA

Sa-CBE-EMX1#2-693	EMX1#2-2-Fwd	GACCATCAGCCCATTGCTTGTCCCTC
	EMX1#2-2-Rev	CTAGGGTGGGCAACCACAAA
Sa-ABE-EMX1#2-N	EMX1#2-3-Fwd	GAGTTCGAGCCCATTGCTTGTCCCTC
	EMX1#2-3-Rev	CTAGGGTGGGCAACCACAAA
Sa-ABE-EMX1#2-693	EMX1#2-4-Fwd	GTACTTGAGCCCATTGCTTGTCCCTC
	EMX1#2-4-Rev	CTAGGGTGGGCAACCACAAA
Sa-CABE-EMX1#2-N	EMX1#2-5-Fwd	GTTGCCAAGCCCATTGCTTGTCCCTC
	EMX1#2-5-Rev	CTAGGGTGGGCAACCACAAA
Sa-CABE-EMX1#2-693	EMX1#2-6-Fwd	GCTAAGAAGCCCATTGCTTGTCCCTC
	EMX1#2-6-Rev	CTAGGGTGGGCAACCACAAA
Sa-CBE-EMX1#6-N	EMX1#6-1-Fwd	GCCTCTACGAGCAGAAGAAGAAGGGCT
	EMX1#6-1-Rev	TTGTCCCTCTGTCAATGGCG
Sa-CBE-EMX1#6-693	EMX1#6-2-Fwd	AACCTAGCGAGCAGAAGAAGAAGGGCT
	EMX1#6-2-Rev	TTGTCCCTCTGTCAATGGCG
Sa-ABE-EMX1#6-N	EMX1#6-3-Fwd	ATTCTCGCGAGCAGAAGAAGAAGGGCT
	EMX1#6-3-Rev	TTGTCCCTCTGTCAATGGCG
Sa-ABE-EMX1#6-693	EMX1#6-4-Fwd	ATCCGGACGAGCAGAAGAAGAAGGGCT
	EMX1#6-4-Rev	TTGTCCCTCTGTCAATGGCG
Sa-CABE-EMX1#6-N	EMX1#6-5-Fwd	ATGCTTCCGAGCAGAAGAAGAAGGGCT
	EMX1#6-5-Rev	TTGTCCCTCTGTCAATGGCG
Sa-CABE-EMX1#6-693	EMX1#6-6-Fwd	ACCATCGCGAGCAGAAGAAGAAGGGCT
	EMX1#6-6-Rev	TTGTCCCTCTGTCAATGGCG

Table S8.

DNA off-target HTS Primers used to amplify each target

Sample name	Primer name	Sequence
R-LOOP-HEK4-N	HEK4-1-Fwd	GAGCGATAGATGGCTGACAAAGGCCG
	HEK4-1-Rev	AACCCGAACGGAGACACAC
R-LOOP-HEK4-125	HEK4-2-Fwd	GTTCAACAGATGGCTGACAAAGGCCG
	HEK4-2-Rev	AACCCGAACGGAGACACAC
R-LOOP-HEK4-269	HEK4-3-Fwd	GTGGTACAGATGGCTGACAAAGGCCG
	HEK4-3-Rev	AACCCGAACGGAGACACAC
R-LOOP-HEK4-593	HEK4-4-Fwd	GCTCATTAGATGGCTGACAAAGGCCG
	HEK4-4-Rev	AACCCGAACGGAGACACAC
R-LOOP-HEK4-693	HEK4-5-Fwd	GCGAGTTAGATGGCTGACAAAGGCCG
	HEK4-5-Rev	AACCCGAACGGAGACACAC
R-LOOP-SiteB-N	SiteB-1-Fwd	ATTGCTCGTCTCCACACAGGTGCTGTT
	SiteB-1-Rev	AACTGCCAAAAGCCACAACC
R-LOOP-SiteB-125	SiteB-2-Fwd	ATCAAGCGTCTCCACACAGGTGCTGTT
	SiteB-2-Rev	AACTGCCAAAAGCCACAACC
R-LOOP-SiteB-269	SiteB-3-Fwd	ATCCGGAGTCTCCACACAGGTGCTGTT
	SiteB-3-Rev	AACTGCCAAAAGCCACAACC
R-LOOP-SiteB-593	SiteB-4-Fwd	ATCGAAGGTCTCCACACAGGTGCTGTT
	SiteB-4-Rev	AACTGCCAAAAGCCACAACC
R-LOOP-SiteB-693	SiteB-5-Fwd	ATCGTCCGTCTCCACACAGGTGCTGTT
	SiteB-5-Rev	AACTGCCAAAAGCCACAACC
R-LOOP-PPP-N	PPP-1-Fwd	CAATGTGTGCACCCGGCCATGGTTT
	PPP-1-Rev	CTGAGACTCAGGAGGCCCAGA
R-LOOP-PPP-125	PPP-2-Fwd	CATCTTGTGCACCCGGCCATGGTTT
	PPP-2-Rev	CTGAGACTCAGGAGGCCCAGA
R-LOOP-PPP-269	PPP-3-Fwd	CATGCAATGCACCCGGCCATGGTTT

	PPP-3-Rev	CTGAGACTCAGGAGGCCCAGA
R-LOOP-PPP-593	PPP-4-Fwd	CTACAAGTGCACCCGGCCATGGTTT
	PPP-4-Rev	CTGAGACTCAGGAGGCCCAGA
R-LOOP-PPP-693	PPP-5-Fwd	CTTAGCTTGCACCCGGCCATGGTTT
	PPP-5-Rev	CTGAGACTCAGGAGGCCCAGA
R-LOOP-FANCF-N	FANCF-1-Fwd	CCAATGACATTGCAGAGAGGCGTATCAT
	FANCF-1-Rev	GTGCTGACGTAGGTAGTGCT
R-LOOP-FANCF-125	FANCF-2-Fwd	CGATTCACATTGCAGAGAGGCGTATCAT
	FANCF-2-Rev	GTGCTGACGTAGGTAGTGCT
R-LOOP-FANCF-269	FANCF-3-Fwd	CGTCCATCATTGCAGAGAGGCGTATCAT
	FANCF-3-Rev	GTGCTGACGTAGGTAGTGCT
R-LOOP-FANCF-593	FANCF-4-Fwd	GAAGGTCCATTGCAGAGAGGCGTATCAT
	FANCF-4-Rev	GTGCTGACGTAGGTAGTGCT
R-LOOP-FANCF-693	FANCF-5-Fwd	GATGACGCATTGCAGAGAGGCGTATCAT
	FANCF-5-Rev	GTGCTGACGTAGGTAGTGCT
R-LOOP-Site29-CTR	Site29-1-Fwd	ATTGCTCCAGGGGAGCGTGTCCATAGG
	Site29-1-Rev	CAGGACCCGGGTTCATAACT
R-LOOP-Site29-N	Site29-2-Fwd	ATCGAAGCAGGGGAGCGTGTCCATAGG
	Site29-2-Rev	CAGGACCCGGGTTCATAACT
R-LOOP-Site29-125	Site29-3-Fwd	ATGGCGACAGGGGAGCGTGTCCATAGG
	Site29-3-Rev	CAGGACCCGGGTTCATAACT
R-LOOP-Site29-269	Site29-4-Fwd	ACCGGTTCAGGGGAGCGTGTCCATAGG
	Site29-4-Rev	CAGGACCCGGGTTCATAACT
R-LOOP-Site29-593	Site29-5-Fwd	TACTCCGCAGGGGAGCGTGTCCATAGG
	Site29-5-Rev	CAGGACCCGGGTTCATAACT
R-LOOP-Site29-693	Site29-6-Fwd	TAGAACGCAGGGGAGCGTGTCCATAGG
	Site29-6-Rev	CAGGACCCGGGTTCATAACT

R-LOOP-HEK3-CTR	HEK3-1-Fwd	TTCCGATTGCATTTGTAGGCTTGATGC
	HEK3-1-Rev	AGGAGCTGCACATACTAGCC
R-LOOP-HEK3-N	HEK3-2-Fwd	TTGCAGCTGCATTTGTAGGCTTGATGC
	HEK3-2-Rev	AGGAGCTGCACATACTAGCC
R-LOOP-HEK3-125	HEK3-3-Fwd	TCGAATCTGCATTTGTAGGCTTGATGC
	HEK3-3-Rev	AGGAGCTGCACATACTAGCC
R-LOOP-HEK3-269	HEK3-4-Fwd	TGCAAGGTGCATTTGTAGGCTTGATGC
	HEK3-4-Rev	AGGAGCTGCACATACTAGCC
R-LOOP-HEK3-593	HEK3-5-Fwd	CAATAGCTGCATTTGTAGGCTTGATGC
	HEK3-5-Rev	AGGAGCTGCACATACTAGCC
R-LOOP-HEK3-693	HEK3-6-Fwd	CCTTATGTGCATTTGTAGGCTTGATGC
	HEK3-6-Rev	AGGAGCTGCACATACTAGCC

Table S9.

BCL11A and PAH HTS Primers used to amplify each target

Sample name	Primer name	Sequence
BCL11A-ABE-N	BCL11A-1-Fwd	TCAGTACCATAGGCCAGAAAAGAGATATGGCA
	BCL11A-1-Rev	AAACGGCCACCGATGGAG
BCL11A-CBE-N	BCL11A-2-Fwd	TCTCGAACATAGGCCAGAAAAGAGATATGGCA
	BCL11A-2-Rev	AAACGGCCACCGATGGAG
BCL11A-CABE-N	BCL11A-3-Fwd	TCCTGGACATAGGCCAGAAAAGAGATATGGCA
	BCL11A-3-Rev	AAACGGCCACCGATGGAG
BCL11A-ABE-693	BCL11A-4-Fwd	TGATCGTCATAGGCCAGAAAAGAGATATGGCA
	BCL11A-4-Rev	AAACGGCCACCGATGGAG
BCL11A-CBE-693	BCL11A-5-Fwd	TGTACTCCATAGGCCAGAAAAGAGATATGGCA
	BCL11A-5-Rev	AAACGGCCACCGATGGAG
BCL11A-CABE-693	BCL11A-6-Fwd	CAATGTGCATAGGCCAGAAAAGAGATATGGCA
	BCL11A-6-Rev	AAACGGCCACCGATGGAG
PAH-1-N	PAH-1-N-Fwd	GTAACCGTGTAAAACGACGGCCAGT
	PAH-1-N-Rev	ATGCCTTTATGGAGTATCA
PAH-1-125	PAH-1-125-Fwd	GCCAATGTGTAAAACGACGGCCAGT
	PAH-1-125-Rev	ATGCCTTTATGGAGTATCA
PAH-2-N	PAH-2-N-Fwd	GGCCTAAGCCACTGTCCGTGAGCT
	PAH-2-N-Rev	CAGGAAACAGCTATGAC
PAH-2-125	PAH-2-125-Fwd	ATACCGCGCCACTGTCCGTGAGCT
	PAH-2-125-Rev	CAGGAAACAGCTATGAC

Supplementary Sequences1.

Amino acid sequences of hA3A(130F), TadA-8e, Sa-CBE-N, SaCBE-125, SaCBE-269, SaCBE-593, SaCBE-693, Sa-ABE-N, SaABE-125, SaABE-269, SaABE-593, SaABE-693, Sa-CABE, Sa-CABE-693.

hA3A(130F)

MEASPASGPRHLMDPHIFTSNFNNGIGRHKTYLCYEVERLDNGTSVKMDQHRGFLHNQAKNL
LCGFYGRHAELRFLDLVPSLQLDPAQIYRVTWFISWSPCFSWGCAGEVRAFLQENTHVRLRIF
AARIFDYDPLYKEALQMLRDAGAQVSIMTYDEFKHCWDTFVDHQGCPFQPWDGLDEHSQAL
SGRLRAILQNQGN

TadA-8e

SEVEFSHEYWMRHALTLAKRARDEREVPVGAVLVLNNRVIGEGWNRAIGLHDPTAHAEIMA
LRQGGLVMQNYRLIDATLYVTFEPCVMCAGAMIHSRIGRVVFGVRNSKRGAAGSLMNVLNY
PGMNHRVEITEGILADECAALLCDFYRMPRQVFNAQKKAQSSIN

Sa-CBE-N

MEASPASGPRHLMDPHIFTSNFNNGIGRHKTYLCYEVERLDNGTSVKMDQHRGFLHNQAKNL
LCGFYGRHAELRFLDLVPSLQLDPAQIYRVTWFISWSPCFSWGCAGEVRAFLQENTHVRLRIF
AARIFDYDPLYKEALQMLRDAGAQVSIMTYDEFKHCWDTFVDHQGCPFQPWDGLDEHSQAL
SGRLRAILQNQGNSGSETPGTSESATPESGKRNYILGLAIGITSVGYGIIDYETRDVIDAGVRLFK
EANVENNEGRRSKRGARRLKRRRRHRIQRVKKLLFDYNLLTDHSELSGINPYEARVKGLSQKL
SEEEFSAALLHLAKRRGVHNVNEVEEDTGNELSTKEQISRNSKALEEKYVAELQLERLKKDGE
VRGSINRFKTSDYVKEAKQLLKVQKAYHQLDQSFIDTYIDLLETRRTYYEGPGEGSPFGWKDI
KEWYEMLMGHCTYFPEELRSVKYAYNADLYNALNDLNNLVITRDENEKLEYYEKFQIIENVF
KQKKKPTLKQIAKEILVNEEDIKGYRVTSTGKPEFTNLKVYHDIKDITARKEIIENAELLDQIAKI
LTIYQSSEDIQEELTNLNSELTQEEIEQISNLKGYTGTHNLSLKAINLILDELWHTNDNQIAIFNR
LKLVPKKVDLSQQKEIPTTLVDDFILSPVVKRSFIQSIKVINAIIKKYGLPNDIIIELAREKNSKDA
QKMINEMQKRNRQTNERIEEIIRTTGKENAKYLIEKIKLHDMQEGKCLYSLEAIPLEDLLNNPF
NYEVDHIIPRSVSFDNSFNNKVLVKQEENSKKGNRTPFQYLSSSDSKISYETFKKHILNLAKGK
GRISKTKKEYLLEERDINRFSVQKDFINRNLVDTRYATRGLMNLLRSYFRVNNLDVKVKSING
GFTSFLRRKWKFKKERNKGYKHHAEDALIIANADFIFKEWKKLDKAKKVMENQMFEEKQAE

SMPEIETEQEYKEIFITPHQIKHIKDFKDYKYSHRVDKKPNRKLINDTLYSTRKDDKGNTLIVNN

LNGLYDKDNDKLKKLINKSPEKLLMYHHDPQTYQKLKLIMEQYGDEKNPLYKYYEETGNYL

TKYSKKDNGPVIKKIKYYGNKLNAHLDITDDYPNSRNKVVKLSLKPYRFDVYLDNGVYKFVT

VKNLDVIKKENYYEVNSKCYEEAKKLKKISNQAEFIASFYKNDLIKINGELYRVIGVNNDLLN

RIEVNMIDITYREYLENMNDKRPPHIIKTIASKTQSIKKYSTDILGNLYEVKSKKHPQIIKKGGSS

GGSTNLSDIIEKETGKQLVIQESILMLPEEVEEVIGNKPESDILVHTAYDESTDENVMLLTSDAP

EYKPWALVIQDSNGENKIKMLSGGSPKKKRKV

Sa-CBE-125

KRNYILGLAIGITSVGYGIIDYETRDVIDAGVRLFKEANVENNEGRRSKRGARRLKRRRRHRIQ

RVKKLLFDYNLLTDHSELSGINPYEARVKGLSQKLSEEEFSAALLHLAKRRGVHNVNEVEESG

SETPGTSESATPESMEASPASGPRHLMDPHIFTSNFNNGIGRHKTYLCYEVERLDNGTSVKMD

QHRGFLHNQAKNLLCGFYGRHAELRFLDLVPSLQLDPAQIYRVTWFISWSPCFSWGCAGEVR

AFLQENTHVRLRIFAARIFDYDPLYKEALQMLRDAGAQVSIMTYDEFKHCWDTFVDHQGCPF

QPWDGLDEHSQALSGRLRAILQNQGNSGSETPGTSESATPESDTGNELSTKEQISRNSKALEEK

YVAELQLERLKKDGEVRGSINRFKTSDYVKEAKQLLKVQKAYHQLDQSFIDTYIDLLETRRTY

YEGPGEGSPFGWKDIKEWYEMLMGHCTYFPEELRSVKYAYNADLYNALNDLNNLVITRDEN

EKLEYYEKFQIIENVFKQKKKPTLKQIAKEILVNEEDIKGYRVTSTGKPEFTNLKVYHDIKDITA

RKEIIENAELLDQIAKILTIYQSSEDIQEELTNLNSELTQEEIEQISNLKGYTGTHNLSLKAINLILD

ELWHTNDNQIAIFNRLKLVPKKVDLSQQKEIPTTLVDDFILSPVVKRSFIQSIKVINAIIKKYGLP

NDIIIELAREKNSKDAQKMINEMQKRNRQTNERIEEIIRTTGKENAKYLIEKIKLHDMQEGKCL

YSLEAIPLEDLLNNPFNYEVDHIIPRSVSFDNSFNNKVLVKQEENSKKGNRTPFQYLSSSDSKIS

YETFKKHILNLAKGKGRISKTKKEYLLEERDINRFSVQKDFINRNLVDTRYATRGLMNLLRSYF

RVNNLDVKVKSINGGFTSFLRRKWKFKKERNKGYKHHAEDALIIANADFIFKEWKKLDKAKK

VMENQMFEEKQAESMPEIETEQEYKEIFITPHQIKHIKDFKDYKYSHRVDKKPNRKLINDTLYS

TRKDDKGNTLIVNNLNGLYDKDNDKLKKLINKSPEKLLMYHHDPQTYQKLKLIMEQYGDEK

NPLYKYYEETGNYLTKYSKKDNGPVIKKIKYYGNKLNAHLDITDDYPNSRNKVVKLSLKPYR

FDVYLDNGVYKFVTVKNLDVIKKENYYEVNSKCYEEAKKLKKISNQAEFIASFYKNDLIKING

ELYRVIGVNNDLLNRIEVNMIDITYREYLENMNDKRPPHIIKTIASKTQSIKKYSTDILGNLYEV

KSKKHPQIIKKGGSPKKKRKVSSDYKDHDGDYKDHDIDYKDDDDKSGGSTNLSDIIEKETGKQ

LVIQESILMLPEEVEEVIGNKPESDILVHTAYDESTDENVMLLTSDAPEYKPWALVIQDSNGEN

KIKMLSGGSPKKKRKV

Sa-CBE-269

KRNYILGLAIGITSVGYGIIDYETRDVIDAGVRLFKEANVENNEGRRSKRGARRLKRRRRHRIQ

RVKKLLFDYNLLTDHSELSGINPYEARVKGLSQKLSEEEFSAALLHLAKRRGVHNVNEVEEDT

GNELSTKEQISRNSKALEEKYVAELQLERLKKDGEVRGSINRFKTSDYVKEAKQLLKVQKAY

HQLDQSFIDTYIDLLETRRTYYEGPGEGSPFGWKDIKEWYEMLMGHCTYFPEELRSVKYAYN

ADLYNALNDLNNLVITRDSGSETPGTSESATPESMEASPASGPRHLMDPHIFTSNFNNGIGRHK

TYLCYEVERLDNGTSVKMDQHRGFLHNQAKNLLCGFYGRHAELRFLDLVPSLQLDPAQIYRV

TWFISWSPCFSWGCAGEVRAFLQENTHVRLRIFAARIFDYDPLYKEALQMLRDAGAQVSIMTY

DEFKHCWDTFVDHQGCPFQPWDGLDEHSQALSGRLRAILQNQGNSGSETPGTSESATPESENE

KLEYYEKFQIIENVFKQKKKPTLKQIAKEILVNEEDIKGYRVTSTGKPEFTNLKVYHDIKDITAR

KEIIENAELLDQIAKILTIYQSSEDIQEELTNLNSELTQEEIEQISNLKGYTGTHNLSLKAINLILDE

LWHTNDNQIAIFNRLKLVPKKVDLSQQKEIPTTLVDDFILSPVVKRSFIQSIKVINAIIKKYGLPN

DIIIELAREKNSKDAQKMINEMQKRNRQTNERIEEIIRTTGKENAKYLIEKIKLHDMQEGKCLYS

LEAIPLEDLLNNPFNYEVDHIIPRSVSFDNSFNNKVLVKQEENSKKGNRTPFQYLSSSDSKISYE

TFKKHILNLAKGKGRISKTKKEYLLEERDINRFSVQKDFINRNLVDTRYATRGLMNLLRSYFR

VNNLDVKVKSINGGFTSFLRRKWKFKKERNKGYKHHAEDALIIANADFIFKEWKKLDKAKKV

MENQMFEEKQAESMPEIETEQEYKEIFITPHQIKHIKDFKDYKYSHRVDKKPNRKLINDTLYST

RKDDKGNTLIVNNLNGLYDKDNDKLKKLINKSPEKLLMYHHDPQTYQKLKLIMEQYGDEKN

PLYKYYEETGNYLTKYSKKDNGPVIKKIKYYGNKLNAHLDITDDYPNSRNKVVKLSLKPYRF

DVYLDNGVYKFVTVKNLDVIKKENYYEVNSKCYEEAKKLKKISNQAEFIASFYKNDLIKINGE

LYRVIGVNNDLLNRIEVNMIDITYREYLENMNDKRPPHIIKTIASKTQSIKKYSTDILGNLYEVK

SKKHPQIIKKGGSPKKKRKVSSDYKDHDGDYKDHDIDYKDDDDKSGGSTNLSDIIEKETGKQL

VIQESILMLPEEVEEVIGNKPESDILVHTAYDESTDENVMLLTSDAPEYKPWALVIQDSNGENK

IKMLSGGSPKKKRKV

Sa-CBE-593

KRNYILGLAIGITSVGYGIIDYETRDVIDAGVRLFKEANVENNEGRRSKRGARRLKRRRRHRIQ

RVKKLLFDYNLLTDHSELSGINPYEARVKGLSQKLSEEEFSAALLHLAKRRGVHNVNEVEEDT

GNELSTKEQISRNSKALEEKYVAELQLERLKKDGEVRGSINRFKTSDYVKEAKQLLKVQKAY
HQLDQSFIDTYIDLLETRRTYYEGPGEGSPFGWKDIKEWYEMLMGHCTYFPEELRSVKYAYN
ADLYNALNDLNNLVITRDENEKLEYYEKFQIIENVFKQKKKPTLKQIAKEILVNEEDIKGYRVT
STGKPEFTNLKVYHDIKDITARKEIIENAELLDQIAKILTIYQSSEDIQEELTNLNSELTQEEIEQIS
NLKGYTGTHNLSLKAINLILDELWHTNDNQIAIFNRLKLVPKKVDLSQQKEIPTTLVDDFILSPV
VKRSFIQSIKVINAIIKKYGLPNDIIIELAREKNSKDAQKMINEMQKRNRQTNERIEEIIRTTGKE
NAKYLIEKIKLHDMQEGKCLYSLEAIPLEDLLNNPFNYEVDHIIPRSVSFDNSFNNKVLVKQEE
NSKKGNRTPFQYLSSSGSETPGTSESATPESMEASPASGPRHLMDPHIFTSNFNNGIGRHKTYLC
YEVERLDNGTSVKMDQHRGFLHNQAKNLLCGFYGRHAELRFLDLVPSLQLDPAQIYRVTWFI
SWSPCFSWGCAGEVRAFLQENTHVRLRIFAARIFDYDPLYKEALQMLRDAGAQVSIMTYDEF
KHCWDTFVDHQGCPFQPWDGLDEHSQALSGRLRAILQNQGNSGSETPGTSESATPESSDSKIS
YETFKKHILNLAKGKGRISKTKKEYLLEERDINRFSVQKDFINRNLVDTRYATRGLMNLLRSYF
RVNNLDVKVKSINGGFTSFLRRKWKFKKERNKGYKHHAEDALIIANADFIFKEWKKLDKAKK
VMENQMFEEKQAESMPEIETEQEYKEIFITPHQIKHIKDFKDYKYSHRVDKKPNRKLINDTLYS
TRKDDKGNTLIVNNLNGLYDKDNDKLKKLINKSPEKLLMYHHDPQTYQKLKLIMEQYGDEK
NPLYKYYEETGNYLTKYSKKDNGPVIKKIKYYGNKLNAHLDITDDYPNSRNKVVKLSLKPYR
FDVYLDNGVYKFVTVKNLDVIKKENYYEVNSKCYEEAKKLKKISNQAEFIASFYKNDLIKING
ELYRVIGVNNDLLNRIEVNMIDITYREYLENMNDKRPPHIIKTIASKTQSIKKYSTDILGNLYEV
KSKKHPQIIKKGGSPKKKRKVSSDYKDHDGDYKDHDIDYKDDDDKSGGSTNLSDIIEKETGKQ
LVIQESILMLPEEVEEVIGNKPESDILVHTAYDESTDENVMLLTSDAPEYKPWALVIQDSNGEN
KIKMLSGGSPKKKRKV

Sa-CBE-693

KRNYILGLAIGITSVGYGIIDYETRDVIDAGVRLFKEANVENNEGRRSKRGARRLKRRRRHRIQ
RVKKLLFDYNLLTDHSELSGINPYEARVKGLSQKLSEEEFSAALLHLAKRRGVHNVNEVEEDT
GNELSTKEQISRNSKALEEKYVAELQLERLKKDGEVRGSINRFKTSDYVKEAKQLLKVQKAY
HQLDQSFIDTYIDLLETRRTYYEGPGEGSPFGWKDIKEWYEMLMGHCTYFPEELRSVKYAYN
ADLYNALNDLNNLVITRDENEKLEYYEKFQIIENVFKQKKKPTLKQIAKEILVNEEDIKGYRVT
STGKPEFTNLKVYHDIKDITARKEIIENAELLDQIAKILTIYQSSEDIQEELTNLNSELTQEEIEQIS
NLKGYTGTHNLSLKAINLILDELWHTNDNQIAIFNRLKLVPKKVDLSQQKEIPTTLVDDFILSPV

VKRSFIQSIKVINAIIKKYGLPNDIIIELAREKNSKDAQKMINEMQKRNRQTNERIEEIIRTTGKE
NAKYLIEKIKLHDMQEGKCLYSLEAIPLEDLLNNPFNYEVDHIIPRSVSFDNSFNNKVLVKQEE
NSKKGNRTPFQYLSSSDSKISYETFKKHILNLAKGKGRISKTKKEYLLEERDINRFSVQKDFINR
NLVDTRYATRGLMNLLRSYFRVNNLDVKVKSINGGFTSFLRRKWKFKKERSGSETPGTSESAT
PESMEASPASGPRHLMDPHIFTSNFNNGIGRHKTYLCYEVERLDNGTSVKMDQHRGFLHNQA
KNLLCGFYGRHAELRFLDLVPSLQLDPAQIYRVTWFISWSPCFSWGCAGEVRAFLQENTHVRL
RIFAARIFDYDPLYKEALQMLRDAGAQVSIMTYDEFKHCWDTFVDHQGCPFQPWDGLDEHSQ
ALSGRLRAILQNQGNSGSETPGTSESATPESNKGYKHHAEDALIIANADFIFKEWKKLDKAKK
VMENQMFEEKQAESMPEIETEQEYKEIFITPHQIKHIKDFKDYKYSHRVDKKPNRKLINDTLYS
TRKDDKGNTLIVNNLNGLYDKDNDKLKKLINKSPEKLLMYHHDPQTYQKLKLIMEQYGDEK
NPLYKYYEETGNYLTKYSKKDNGPVIKKIKYYGNKLNAHLDITDDYPNSRNKVVKLSLKPYR
FDVYLDNGVYKFVTVKNLDVIKKENYYEVNSKCYEEAKKLKKISNQAEFIASFYKNDLIKING
ELYRVIGVNNDLLNRIEVNMIDITYREYLENMNDKRPPHIIKTIASKTQSIKKYSTDILGNLYEV
KSKKHPQIIKKGGSPKKKRKVSSDYKDHDGDYKDHDIDYKDDDDKSGGSTNLSDIIEKETGKQ
LVIQESILMLPEEVEEVIGNKPESDILVHTAYDESTDENVMLLTSDAPEYKPWALVIQDSNGEN
KIKMLSGGSPKKKRKV

Sa-ABE-N

SEVEFSHEYWMRHALTLAKRARDEREVPVGAVLVLNNRVIGEGWNRAIGLHDPTAHAEIMA
LRQGGLVMQNYRLIDATLYVTFEPCVMCAGAMIHSRIGRVVFGVRNSKRGAAGSLMNVLNY
PGMNHRVEITEGILADECAALLCDFYRMPRQVFNAQKKAQSSINSGSETPGTSESATPESGKRN
YILGLAIGITSVGYGIIDYETRDVIDAGVRLFKEANVENNEGRRSKRGARRLKRRRRHRIQRVK
KLLFDYNLLTDHSELSGINPYEARVKGLSQKLSEEEFSAALLHLAKRRGVHNVNEVEEDTGNE
LSTKEQISRNSKALEEKYVAELQLERLKKDGEVRGSINRFKTSDYVKEAKQLLKVQKAYHQL
DQSFIDTYIDLLETRRTYYEGPGEGSPFGWKDIKEWYEMLMGHCTYFPEELRSVKYAYNADL
YNALNDLNNLVITRDENEKLEYYEKFQIIENVFKQKKKPTLKQIAKEILVNEEDIKGYRVTSTG
KPEFTNLKVYHDIKDITARKEIIENAELLDQIAKILTIYQSSEDIQEELTNLNSELTQEEIEQISNLK
GYTGTHNLSLKAINLILDELWHTNDNQIAIFNRLKLVPKKVDLSQQKEIPTTLVDDFILSPVVKR
SFIQSIKVINAIIKKYGLPNDIIIELAREKNSKDAQKMINEMQKRNRQTNERIEEIIRTTGKENAK
YLIEKIKLHDMQEGKCLYSLEAIPLEDLLNNPFNYEVDHIIPRSVSFDNSFNNKVLVKQEENSK

KGNRTPFQYLSSSDSKISYETFKKHILNLAKGKGRISKTKKEYLLEERDINRFSVQKDFINRNLV
DTRYATRGLMNLLRSYFRVNNLDVKVKSINGGFTSFLRRKWKFKKERNKGYKHHAEDALIIA
NADFIFKEWKKLDKAKKVMENQMFEEKQAESMPEIETEQEYKEIFITPHQIKHIKDFKDYKYS
HRVDKKPNRKLINDTLYSTRKDDKGNTLIVNNLNGLYDKDNDKLKKLINKSPEKLLMYHHDP
QTYQKLKLIMEQYGDEKNPLYKYYEETGNYLTKYSKKDNGPVIKKIKYYGNKLNAHLDITDD
YPNSRNKVVKLSLKPYRFDVYLDNGVYKFVTVKNLDVIKKENYYEVNSKCYEEAKKLKKISN
QAEFIASFYKNDLIKINGELYRVIGVNNDLLNRIEVNMIDITYREYLENMNDKRPPHIIKTIASKT
QSIKKYSTDILGNLYEVKSKKHPQIIKKGGSSGGSTNLSDIIEKETGKQLVIQESILMLPEEVEEVI
GNKPESDILVHTAYDESTDENVMLLTSDAPEYKPWALVIQDSNGENKIKMLSGGSPKKKRKV

Sa-ABE-125

KRNYILGLAIGITSVGYGIIDYETRDVIDAGVRLFKEANVENNEGRRSKRGARRLKRRRRHRIQ
RVKKLLFDYNLLTDHSELSGINPYEARVKGLSQKLSEEEFSAALLHLAKRRGVHNVNEVEESG
SETPGTSESATPES
SEVEFSHEYWMRHALTLAKRARDEREVPVGAVLVLNNRVIGEGWNRAIGLHDPTAHAEIMA
LRQGGLVMQNYRLIDATLYVTFEPCVMCAGAMIHSRIGRVVFGVRNSKRGAAGSLMNVLNY
PGMNHRVEITEGILADECAALLCDFYRMPRQVFNAQKKAQSSINSGSETPGTSESATPESDTGN
ELSTKEQISRNSKALEEKYVAELQLERLKKDGEVRGSINRFKTSDYVKEAKQLLKVQKAYHQL
DQSFIDTYIDLLETRRTYYEGPGEGSPFGWKDIKEWYEMLMGHCTYFPEELRSVKYAYNADL
YNALNDLNNLVITRDENEKLEYYEKFQIIENVFKQKKKPTLKQIAKEILVNEEDIKGYRVTSTG
KPEFTNLKVYHDIKDITARKEIIENAELLDQIAKILTIYQSSEDIQEELTNLNSELTQEEIEQISNLK
GYTGTHNLSLKAINLILDELWHTNDNQIAIFNRLKLVPKKVDLSQQKEIPTTLVDDFILSPVVKR
SFIQSIKVINAIIKKYGLPNDIIIELAREKNSKDAQKMINEMQKRNRQTNERIEEIIRTTGKENAK
YLIEKIKLHDMQEGKCLYSLEAIPLEDLLNNPFNYEVDHIIPRSVSFDNSFNNKVLVKQEENSK
KGNRTPFQYLSSSDSKISYETFKKHILNLAKGKGRISKTKKEYLLEERDINRFSVQKDFINRNLV
DTRYATRGLMNLLRSYFRVNNLDVKVKSINGGFTSFLRRKWKFKKERNKGYKHHAEDALIIA
NADFIFKEWKKLDKAKKVMENQMFEEKQAESMPEIETEQEYKEIFITPHQIKHIKDFKDYKYS
HRVDKKPNRKLINDTLYSTRKDDKGNTLIVNNLNGLYDKDNDKLKKLINKSPEKLLMYHHDP
QTYQKLKLIMEQYGDEKNPLYKYYEETGNYLTKYSKKDNGPVIKKIKYYGNKLNAHLDITDD
YPNSRNKVVKLSLKPYRFDVYLDNGVYKFVTVKNLDVIKKENYYEVNSKCYEEAKKLKKISN

QAEFIASFYKNDLIKINGELYRVIGVNNDLLNRIEVNMIDITYREYLENMNDKRPPHIIKTIASKT
QSIKKYSTDILGNLYEVKSKKHPQIIKKGGSPKKKRKVSSDYKDHDGDYKDHDIDYKDDDDK
SGGSTNLSDIIEKETGKQLVIQESILMLPEEVEEVIGNKPESDILVHTAYDESTDENVMLLTSDA
PEYKPWALVIQDSNGENKIKMLSGGSPKKKRKV

Sa-ABE-269

KRNYILGLAIGITSVGYGIIDYETRDVIDAGVRLFKEANVENNEGRRSKRGARRLKRRRRHRIQ
RVKKLLFDYNLLTDHSELSGINPYEARVKGLSQKLSEEEFSAALLHLAKRRGVHNVNEVEEDT
GNELSTKEQISRNSKALEEKYVAELQLERLKKDGEVRGSINRFKTSDYVKEAKQLLKVQKAY
HQLDQSFIDTYIDLLETRRTYYEGPGEGSPFGWKDIKEWYEMLMGHCTYFPEELRSVKYAYN
ADLYNALNDLNNLVITRDSGSETPGTSESATPESSEVEFSHEYWMRHALTLAKRARDEREVPV
GAVLVLNNRVIGEGWNRAIGLHDPTAHAEIMALRQGGLVMQNYRLIDATLYVTFEPCVMCA
GAMIHSRIGRVVFGVRNSKRGAAGSLMNVLNYPGMNHRVEITEGILADECAALLCDFYRMPR
QVFNAQKKAQSSINSGSETPGTSESATPESENEKLEYYEKFQIIENVFKQKKKPTLKQIAKEILV
NEEDIKGYRVTSTGKPEFTNLKVYHDIKDITARKEIIENAELLDQIAKILTIYQSSEDIQEELTNL
NSELTQEEIEQISNLKGYTGTHNLSLKAINLILDELWHTNDNQIAIFNRLKLVPKKVDLSQQKEI
PTTLVDDFILSPVVKRSFIQSIKVINAIIKKYGLPNDIIIELAREKNSKDAQKMINEMQKRNRQTN
ERIEEIIRTTGKENAKYLIEKIKLHDMQEGKCLYSLEAIPLEDLLNNPFNYEVDHIIPRSVSFDNS
FNNKVLVKQEENSKKGNRTPFQYLSSSDSKISYETFKKHILNLAKGKGRISKTKKEYLLEERDI
NRFSVQKDFINRNLVDTRYATRGLMNLLRSYFRVNNLDVKVKSINGGFTSFLRRKWKFKKER
NKGYKHHAEDALIIANADFIFKEWKKLDKAKKVMENQMFEEKQAESMPEIETEQEYKEIFITP
HQIKHIKDFKDYKYSHRVDKKPNRKLINDTLYSTRKDDKGNTLIVNNLNGLYDKDNDKLKKL
INKSPEKLLMYHHDPQTYQKLKLIMEQYGDEKNPLYKYYEETGNYLTKYSKKDNGPVIKKIK
YYGNKLNAHLDITDDYPNSRNKVVKLSLKPYRFDVYLDNGVYKFVTVKNLDVIKKENYYEV
NSKCYEEAKKLKKISNQAEFIASFYKNDLIKINGELYRVIGVNNDLLNRIEVNMIDITYREYLEN
MNDKRPPHIIKTIASKTQSIKKYSTDILGNLYEVKSKKHPQIIKKGGSPKKKRKVSSDYKDHDG
DYKDHDIDYKDDDDKSGGSTNLSDIIEKETGKQLVIQESILMLPEEVEEVIGNKPESDILVHTAY
DESTDENVMLLTSDAPEYKPWALVIQDSNGENKIKMLSGGSPKKKRKV

Sa-ABE-593

KRNYILGLAIGITSVGYGIIDYETRDVIDAGVRLFKEANVENNEGRRSKRGARRLKRRRRHRIQ
RVKKLLFDYNLLTDHSELSGINPYEARVKGLSQKLSEEEFSAALLHLAKRRGVHNVNEVEEDT
GNELSTKEQISRNSKALEEKYVAELQLERLKKDGEVRGSINRFKTSDYVKEAKQLLKVQKAY
HQLDQSFIDTYIDLLETRRTYYEGPGEGSPFGWKDIKEWYEMLMGHCTYFPEELRSVKYAYN
ADLYNALNDLNNLVITRDENEKLEYYEKFQIIENVFKQKKKPTLKQIAKEILVNEEDIKGYRVT
STGKPEFTNLKVYHDIKDITARKEIIENAELLDQIAKILTIYQSSEDIQEELTNLNSELTQEEIEQIS
NLKGYTGTHNLSLKAINLILDELWHTNDNQIAIFNRLKLVPKKVDLSQQKEIPTTLVDDFILSPV
VKRSFIQSIKVINAIIKKYGLPNDIIIELAREKNSKDAQKMINEMQKRNRQTNERIEEIIRTTGKE
NAKYLIEKIKLHDMQEGKCLYSLEAIPLEDLLNNPFNYEVDHIIPRSVSFDNSFNNKVLVKQEE
NSKKGNRTPFQYLSSSGSETPGTSESATPESSEVEFSHEYWMRHALTLAKRARDEREVPVGAV
LVLNNRVIGEGWNRAIGLHDPTAHAEIMALRQGGLVMQNYRLIDATLYVTFEPCVMCAGAMI
HSRIGRVVFGVRNSKRGAAGSLMNVLNYPGMNHRVEITEGILADECAALLCDFYRMPRQVFN
AQKKAQSSINSGSETPGTSESATPESSDSKISYETFKKHILNLAKGKGRISKTKKEYLLEERDINR
FSVQKDFINRNLVDTRYATRGLMNLLRSYFRVNNLDVKVKSINGGFTSFLRRKWKFKKERNK
GYKHHAEDALIIANADFIFKEWKKLDKAKKVMENQMFEEKQAESMPEIETEQEYKEIFITPHQI
KHIKDFKDYKYSHRVDKKPNRKLINDTLYSTRKDDKGNTLIVNNLNGLYDKDNDKLKKLINK
SPEKLLMYHHDPQTYQKLKLIMEQYGDEKNPLYKYYEETGNYLTKYSKKDNGPVIKKIKYYG
NKLNAHLDITDDYPNSRNKVVKLSLKPYRFDVYLDNGVYKFVTVKNLDVIKKENYYEVNSK
CYEEAKKLKKISNQAEFIASFYKNDLIKINGELYRVIGVNNDLLNRIEVNMIDITYREYLENMN
DKRPPHIIKTIASKTQSIKKYSTDILGNLYEVKSKKHPQIIKKGGSPKKKRKVSSDYKDHDGDY
KDHDIDYKDDDDKSGGSTNLSDIIEKETGKQLVIQESILMLPEEVEEVIGNKPESDILVHTAYDE
STDENVMLLTSDAPEYKPWALVIQDSNGENKIKMLSGGSPKKKRKV

Sa-ABE-693

KRNYILGLAIGITSVGYGIIDYETRDVIDAGVRLFKEANVENNEGRRSKRGARRLKRRRRHRIQ
RVKKLLFDYNLLTDHSELSGINPYEARVKGLSQKLSEEEFSAALLHLAKRRGVHNVNEVEEDT
GNELSTKEQISRNSKALEEKYVAELQLERLKKDGEVRGSINRFKTSDYVKEAKQLLKVQKAY
HQLDQSFIDTYIDLLETRRTYYEGPGEGSPFGWKDIKEWYEMLMGHCTYFPEELRSVKYAYN
ADLYNALNDLNNLVITRDENEKLEYYEKFQIIENVFKQKKKPTLKQIAKEILVNEEDIKGYRVT
STGKPEFTNLKVYHDIKDITARKEIIENAELLDQIAKILTIYQSSEDIQEELTNLNSELTQEEIEQIS

NLKGYTGTHNLSLKAINLILDELWHTNDNQIAIFNRLKLVPKKVDLSQQKEIPTTLVDDFILSPV
VKRSFIQSIKVINAIIKKYGLPNDIIIELAREKNSKDAQKMINEMQKRNRQTNERIEEIIRTTGKE
NAKYLIEKIKLHDMQEGKCLYSLEAIPLEDLLNNPFNYEVDHIIPRSVSFDNSFNNKVLVKQEE
NSKKGNRTPFQYLSSSDSKISYETFKKHILNLAKGKGRISKTKKEYLLEERDINRFSVQKDFINR
NLVDTRYATRGLMNLLRSYFRVNNLDVKVKSINGGFTSFLRRKWKFKKERSGSETPGTSESAT
PESSEVEFSHEYWMRHALTLAKRARDEREVPVGAVLVLNNRVIGEGWNRAIGLHDPTAHAEI
MALRQGGLVMQNYRLIDATLYVTFEPCVMCAGAMIHSRIGRVVFGVRNSKRGAAGSLMNVL
NYPGMNHRVEITEGILADECAALLCDFYRMPRQVFNAQKKAQSSINSGSETPGTSESATPESNK
GYKHHAEDALIIANADFIFKEWKKLDKAKKVMENQMFEEKQAESMPEIETEQEYKEIFITPHQI
KHIKDFKDYKYSHRVDKKPNRKLINDTLYSTRKDDKGNTLIVNNLNGLYDKDNDKLKKLINK
SPEKLLMYHHDPQTYQKLKLIMEQYGDEKNPLYKYYEETGNYLTKYSKKDNGPVIKKIKYYG
NKLNAHLDITDDYPNSRNKVVKLSLKPYRFDVYLDNGVYKFVTVKNLDVIKKENYYEVNSK
CYEEAKKLKKISNQAEFIASFYKNDLIKINGELYRVIGVNNDLLNRIEVNMIDITYREYLENMN
DKRPPHIIKTIASKTQSIKKYSTDILGNLYEVKSKKHPQIIKKGGSPKKKRKVSSDYKDHDGDY
KDHDIDYKDDDDKSGGSTNLSDIIEKETGKQLVIQESILMLPEEVEEVIGNKPESDILVHTAYDE
STDENVMLLTSDAPEYKPWALVIQDSNGENKIKMLSGGSPKKKRKV

Sa-CABE-N

SEVEFSHEYWMRHALTLAKRARDEREVPVGAVLVLNNRVIGEGWNRAIGLHDPTAHAEIMA
LRQGGLVMQNYRLIDATLYVTFEPCVMCAGAMIHSRIGRVVFGVRNSKRGAAGSLMNVLNY
PGMNHRVEITEGILADECAALLCDFYRMPRQVFNAQKKAQSSINSGSETPGTSESATPESMEAS
PASGPRHLMDPHIFTSNFNNGIGRHKTYLCYEVERLDNGTSVKMDQHRGFLHNQAKNLLCGF
YGRHAELRFLDLVPSLQLDPAQIYRVTWFISWSPCFSWGCAGEVRAFLQENTHVRLRIFAARIF
DYDPLYKEALQMLRDAGAQVSIMTYDEFKHCWDTFVDHQGCPFQPWDGLDEHSQALSGRLR
AILQNQGNSGSETPGTSESATPESGKRNYILGLAIGITSVGYGIIDYETRDVIDAGVRLFKEANV
ENNEGRRSKRGARRLKRRRRHRIQRVKKLLFDYNLLTDHSELSGINPYEARVKGLSQKLSEEE
FSAALLHLAKRRGVHNVNEVEEDTGNELSTKEQISRNSKALEEKYVAELQLERLKKDGEVRG
SINRFKTSDYVKEAKQLLKVQKAYHQLDQSFIDTYIDLLETRRTYYEGPGEGSPFGWKDIKEW
YEMLMGHCTYFPEELRSVKYAYNADLYNALNDLNNLVITRDENEKLEYYEKFQIIENVFKQK
KKPTLKQIAKEILVNEEDIKGYRVTSTGKPEFTNLKVYHDIKDITARKEIIENAELLDQIAKILTI

YQSSEDIQEELTNLNSELTQEEIEQISNLKGYTGTHNLSLKAINLILDELWHTNDNQIAIFNRLKL
VPKKVDLSQQKEIPTTLVDDFILSPVVKRSFIQSIKVINAIIKKYGLPNDIIIELAREKNSKDAQK
MINEMQKRNRQTNERIEEIIRTTGKENAKYLIEKIKLHDMQEGKCLYSLEAIPLEDLLNNPFNY
EVDHIIPRSVSFDNSFNNKVLVKQEENSKKGNRTPFQYLSSSDSKISYETFKKHILNLAKGKGRI
SKTKKEYLLEERDINRFSVQKDFINRNLVDTRYATRGLMNLLRSYFRVNNLDVKVKSINGGFT
SFLRRKWKFKKERNKGYKHHAEDALIIANADFIFKEWKKLDKAKKVMENQMFEEKQAESMP
EIETEQEYKEIFITPHQIKHIKDFKDYKYSHRVDKKPNRKLINDTLYSTRKDDKGNTLIVNNLNG
LYDKDNDKLKKLINKSPEKLLMYHHDPQTYQKLKLIMEQYGDEKNPLYKYYEETGNYLTKY
SKKDNGPVIKKIKYYGNKLNAHLDITDDYPNSRNKVVKLSLKPYRFDVYLDNGVYKFVTVKN
LDVIKKENYYEVNSKCYEEAKKLKKISNQAEFIASFYKNDLIKINGELYRVIGVNNDLLNRIEV
NMIDITYREYLENMNDKRPPHIIKTIASKTQSIKKYSTDILGNLYEVKSKKHPQIIKKGGSSGGST
NLSDIIEKETGKQLVIQESILMLPEEVEEVIGNKPESDILVHTAYDESTDENVMLLTSDAPEYKP
WALVIQDSNGENKIKMLSGGSPKKKRKV

Sa-CABE-693

KRNYILGLAIGITSVGYGIIDYETRDVIDAGVRLFKEANVENNEGRRSKRGARRLKRRRRHRIQ
RVKKLLFDYNLLTDHSELSGINPYEARVKGLSQKLSEEEFSAALLHLAKRRGVHNVNEVEEDT
GNELSTKEQISRNSKALEEKYVAELQLERLKKDGEVRGSINRFKTSDYVKEAKQLLKVQKAY
HQLDQSFIDTYIDLLETRRTYYEGPGEGSPFGWKDIKEWYEMLMGHCTYFPEELRSVKYAYN
ADLYNALNDLNNLVITRDENEKLEYYEKFQIIENVFKQKKKPTLKQIAKEILVNEEDIKGYRVT
STGKPEFTNLKVYHDIKDITARKEIIENAELLDQIAKILTIYQSSEDIQEELTNLNSELTQEEIEQIS
NLKGYTGTHNLSLKAINLILDELWHTNDNQIAIFNRLKLVPKKVDLSQQKEIPTTLVDDFILSPV
VKRSFIQSIKVINAIIKKYGLPNDIIIELAREKNSKDAQKMINEMQKRNRQTNERIEEIIRTTGKE
NAKYLIEKIKLHDMQEGKCLYSLEAIPLEDLLNNPFNYEVDHIIPRSVSFDNSFNNKVLVKQEE
NSKKGNRTPFQYLSSSDSKISYETFKKHILNLAKGKGRISKTKKEYLLEERDINRFSVQKDFINR
NLVDTRYATRGLMNLLRSYFRVNNLDVKVKSINGGFTSFLRRKWKFKKERSGSETPGTSESAT
PESSEVEFSHEYWMRHALTLAKRARDEREVPVGAVLVLNNRVIGEGWNRAIGLHDPTAHAEI
MALRQGGLVMQNYRLIDATLYVTFEPCVMCAGAMIHSRIGRVVFGVRNSKRGAAGSLMNVL
NYPGMNHRVEITEGILADECAALLCDFYRMPRQVFNAQKKAQSSINSGSETPGTSESATPESM
EASPASGPRHLMDPHIFTSNFNNGIGRHKTYLCYEVERLDNGTSVKMDQHRGFLHNQAKNLL

CGFYGRHAELRFLDLVPSLQLDPAQIYRVTWFISWSPCFSWGCAGEVRAFLQENTHVRLRIFA

ARIFDYDPLYKEALQMLRDAGAQVSIMTYDEFKHCWDTFVDHQGCPFQPWDGLDEHSQALS

GRLRAILQNQGNSGSETPGTSESATPESNKGYKHHAEDALIIANADFIFKEWKKLDKAKKVME

NQMFEEKQAESMPEIETEQEYKEIFITPHQIKHIKDFKDYKYSHRVDKKPNRKLINDTLYSTRK

DDKGNTLIVNNLNGLYDKDNDKLKKLINKSPEKLLMYHHDPQTYQKLKLIMEQYGDEKNPL

YKYYEETGNYLTKYSKKDNGPVIKKIKYYGNKLNAHLDITDDYPNSRNKVVKLSLKPYRFDV

YLDNGVYKFVTVKNLDVIKKENYYEVNSKCYEEAKKLKKISNQAEFIASFYKNDLIKINGELY

RVIGVNNDLLNRIEVNMIDITYREYLENMNDKRPPHIIKTIASKTQSIKKYSTDILGNLYEVKSK

KHPQIIKKGGSPKKKRKVSSDYKDHDGDYKDHDIDYKDDDDKSGGSTNLSDIIEKETGKQLVI

QESILMLPEEVEEVIGNKPESDILVHTAYDESTDENVMLLTSDAPEYKPWALVIQDSNGENKIK

MLSGGSPKKKRKV

References

1. Kleinstiver BP, Prew MS, Tsai SQ, Nguyen NT, Topkar VV, Zheng Z, et al. Broadening the targeting range of Staphylococcus aureus CRISPR-Cas9 by modifying PAM recognition. Nature Biotechnol. 2015; 33: 1293-8.
2. Yao X, Liu X, Zhang Y, Li Y, Zhao C, Yao S, et al. Gene Therapy of Adult Neuronal Ceroid Lipofuscinoses with CRISPR/Cas9 in Zebrafish. Hum Gene Ther. 2017; 28: 588-97.
3. Komor AC, Zhao KT, Packer MS, Gaudelli NM, Waterbury AL, Koblan LW, et al. Improved base excision repair inhibition and bacteriophage Mu Gam protein yields C:G-to-T:A base editors with higher efficiency and product purity. Sci Adv. 2017; 3: eaao4774.
4. Xie H, Ge X, Yang F, Wang B, Li S, Duan J, et al. High-fidelity SaCas9 identified by directional screening in human cells. PLoS Biol. 2020; 18: e3000747.
5. Nguyen Tran MT, Mohd Khalid MKN, Wang Q, Walker JKR, Lidgerwood GE, Dilworth KL, et al. Engineering domain-inlaid SaCas9 adenine base editors with reduced RNA off-targets and increased on-target DNA editing. Nat Commun. 2020; 11: 4871.
6. Kim YB, Komor AC, Levy JM, Packer MS, Zhao KT, Liu DR. Increasing the genome-targeting scope and precision of base editing with engineered Cas9-cytidine deaminase fusions. Nature Biotechnol. 2017; 35: 371-6.
7. Gehrke JM, Cervantes O, Clement MK, Wu Y, Zeng J, Bauer DE, et al. An APOBEC3A-Cas9 base editor with minimized bystander and off-target activities.

Nature Biotechnol. 2018; 36: 977-82.
8. Komor AC, Kim YB, Packer MS, Zuris JA, Liu DR. Programmable editing of a target base in genomic DNA without double-stranded DNA cleavage. Nature. 2016; 533: 420-4.
9. Huang TP, Zhao KT, Miller SM, Gaudelli NM, Oakes BL, Fellmann C, et al. Circularly permuted and PAM-modified Cas9 variants broaden the targeting scope of base editors. Nature Biotechnol. 2019; 37: 626-31.
